# Device Stochasticity Directly Implements Bayesian Sampling in Spiking Neurons

**DOI:** 10.1002/advs.78508

**Published:** 2026-11-03

**Authors:** Huannan Zheng, Jingli Liu, Kezhou Yang

**Affiliations:** ^1^ MICS Thrust, Function Hub The Hong Kong University of Science and Technology (Guangzhou) Guangdong China

**Keywords:** device uncertainty, probabilistic computing, spiking neural network

## Abstract

Device stochasticity is considered a non‐ideality in neuromorphic hardware, although stochastic neural activity supports probabilistic computation in biological systems. We show that intrinsic stochastic switching of magnetic tunnel junctions (MTJs) can be mapped to stochastic‐threshold spiking neurons, providing a physical sampling primitive for Bayesian spiking inference. We develop a spiking Bayesian neural network (SBNN) with neuronal threshold distributions learned during training and sampled during inference. We also introduce a rate‐estimation method that propagates firing probabilities rather than explicit spike sequences, achieving up to a 22‐fold speedup over surrogate‐gradient training. Compared with representative Bayesian neural‐network baselines, SBNN achieves favorable accuracy‐calibration trade‐offs, reaching high accuracy across static and event datasets and low negative log likelihood in both MNIST and Fashion‐MNIST. On MNIST, SBNN retains 96.20% accuracy under a normalized weight‐noise standard deviation of 0.20 and 81.30% under an input‐noise standard deviation of 0.25, compared with 27.42% and 70.27%, respectively, for the spiking neural network (SNN). Hardware‐in‐the‐loop validation using physical MTJ switching for sampling and software for network arithmetic closely matches software inference in spiking behavior and classification performance. This suggests that intrinsic device stochasticity can be transformed from a hardware non‐ideality into a Bayesian computational resource for compact and noise‐tolerant neuromorphic systems.

## Graphical abstract

Brain‐inspired stochastic spiking turns device noise into Bayesian uncertainty for robust, low‐power neuromorphic computing.

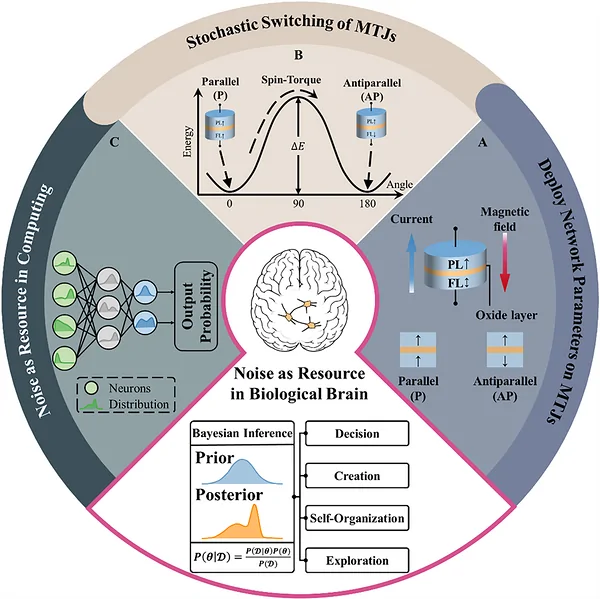

## 1 Introduction

Over the past decade, deep learning has achieved remarkable success across a wide range of artificial intelligence tasks [1]. However, the increasing model complexity and data scale have led to rapidly growing computational and energy costs. Neuromorphic computing aims to address this challenge by drawing inspiration from biological neural systems at both the algorithmic and hardware levels. At the algorithmic level, spiking neural networks (SNNs) emulate neural information processing through discrete spike‐based communication [2, 3]. Because information is transmitted only through sparse spike events, SNNs have shown strong potential for low‐power inference in event‐based vision, edge computing, and neuromorphic sensing [4, 5, 6]. At the hardware level, emerging devices such as resistive random access memory (RRAM) [7, 8], phase‐change memory (PCM) [9], and spintronic devices including magnetic tunnel junctions (MTJs) [10, 11] have been widely explored as artificial neurons and synapses because their intrinsic physical dynamics can emulate neural and synaptic behavior.

A central challenge in such device‐based neuromorphic systems is stochasticity. When neural networks trained in software are deployed on emerging devices, intrinsic fluctuations in resistance states, switching behavior, or neuronal outputs can distort corresponding values during computation and degrade inference reliability [12, 13, 14, 15]. Consequently, device stochasticity is commonly treated as a non‐ideality to be suppressed or compensated by stable device engineering and error‐correction strategies [16, 17, 18].

On the other hand, stochastic dynamics are pervasive in biological neural circuits, where they are increasingly understood as functionally relevant rather than merely detrimental [19, 20, 21, 22, 23]. In biological neural systems, trial‐to‐trial variability has been linked to Bayesian inference through neural sampling theories, in which spike patterns are interpreted as samples from posterior distributions [24, 25, 26, 27], Gaussian or non‐Gaussian, that explicitly represent uncertainty [28, 29, 30, 31, 32, 33]. Such sampling‐based representations have been proposed to underlie key brain functions, including uncertainty representation [28, 31], sensory perception [24, 25], perceptual decision‐making [19, 30, 32, 33], and confidence estimation [30, 33]. Taken together, these observations suggest that the intrinsic stochasticity of neuromorphic devices should be harnessed as a physical substrate for sampling‐based probabilistic inference, rather than treated solely as a non‐ideality to be suppressed [34, 35]. This view aligns with the broader concept of Bayesian electronics, which exploits the intrinsic randomness of emerging nanodevices to implement probabilistic computation and uncertainty estimation in hardware [36].

Based on the observation of stochastic behaviors in biological brains, hardware stochasticity is utilized to construct Bayesian neural network (BNN), which is a computing model proposed to conduct probabilistic inference [37, 38]. The required stochasticity can be obtained from dedicated random‐number generators (RNG) based on stochasticity in additional devices sources [39, 40, 41]. Dedicated RNGs and their distribution circuitry can introduce additional area and energy overhead, depending on the architecture, required numerical precision, and sampling throughput. Recently, researchers showed that inherent stochasticity in synapse devices can be directly utilized for sampling required by BNNs [42, 43], while Wang et al. demonstrated tunable Gaussian weight sampling using stochastic magnetic domain‐wall dynamics [44]. Such a method simplified the circuit design, but requires multi‐bit synapse devices, which makes it difficult to encode precise weight values and scale down [38, 42]. On the other hand, neuronal stochasticity, which is widely observed in biological circuits and represents various distributions, remains under‐explored. Recent device studies have started to expose neuronal‐level stochasticity at the hardware front‐end. However, in most cases the stochasticity is either induced by external noise injection [45] or used only for encoding [46], rather than being intrinsically generated at the neuron event level and explicitly realizing Bayesian inference as an in‐network sampling mechanism akin to biological neuronal computation.

Here, we propose a spiking Bayesian neural network (SBNN) that incorporates uncertainty through learnable neuronal thresholds and uses the stochastic switching of magnetic tunnel junctions (MTJs) as a physical source of stochastic neuronal outputs (Figure 1). This formulation allows neuronal sampling to coexist with deterministic, quantized synaptic weights. To improve training efficiency, we introduce a rate‐estimation (RE) method that propagates estimated firing probabilities without explicitly simulating temporal spike sequences. On the tested 784−1000−10 MNIST network, RE achieves classification accuracy comparable to surrogate‐gradient (SG) training while providing approximately a 22‐fold reduction in training time per epoch at T=64. The stochastic‐threshold formulation also extends to event‐based vision tasks through SG training.

**FIGURE 1:**
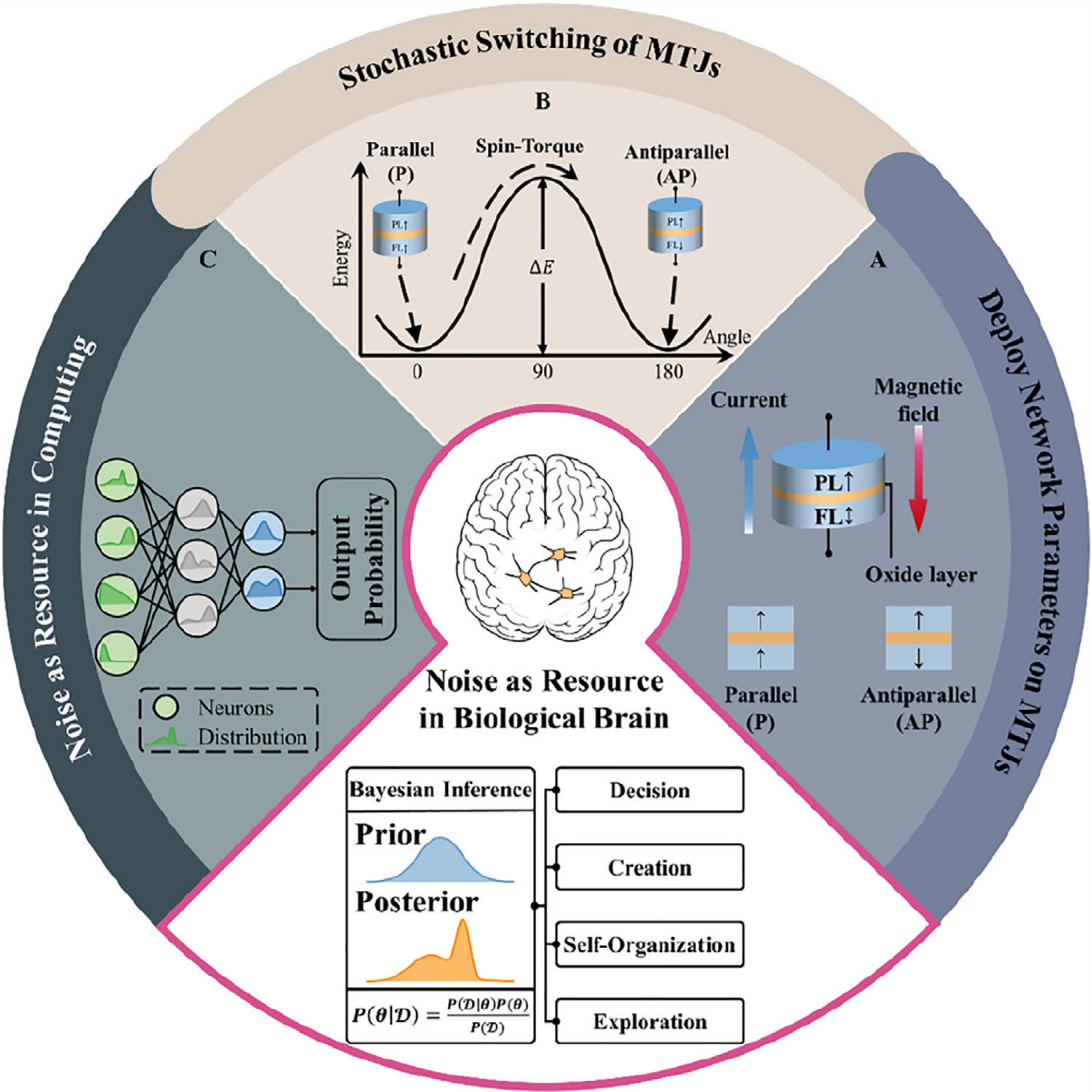
Biological inspiration and device basis of stochastic neuronal sampling. (A) Schematic of a magnetic tunnel junction (MTJ), comprising a pinned layer (PL) and a free layer (FL) separated by an oxide barrier. Parallel (P) and antiparallel (AP) magnetization configurations correspond to low‐ and high‐resistance states, respectively. (B) Schematic energy landscape of the P and AP states, separated by an energy barrier ΔE. Spin torque modifies the switching landscape, while thermal fluctuations contribute to probabilistic transitions between the two states. (C) Conceptual network illustrating neuronal stochasticity as a computational resource. Distributions associated with individual neurons represent threshold uncertainty, which produces stochastic neuronal responses and supports probabilistic prediction in the proposed SBNN. The central brain illustration and lower conceptual panel summarize the biological motivation for treating neuronal variability as a computational resource for probabilistic inference.

In software evaluations, SBNN achieves 99.16% accuracy on MNIST and 90.05% on Fashion‐MNIST with 8‐bit weights, and 94.84% on CIFAR‐10 using a mixed‐precision ResNet‐19. Beyond classification accuracy, the framework provides competitive probabilistic predictions: the MNIST model with 800−800 hidden units achieves a negative log‐likelihood (NLL) of 0.031 and an expected calibration error (ECE) of 0.002. SBNN also exhibits improved robustness under the tested perturbations. Under strong Gaussian synaptic‐weight noise, it retains 96.20% accuracy, compared with 27.42% for the deterministic SNN baseline. Under the tested input‐noise condition, the corresponding accuracies are 81.30% and 70.27%. These results demonstrate that stochastic‐threshold learning can support accurate and noise‐tolerant inference with low‐precision or mixed‐precision weights.

We further validate physical stochastic spike generation through hardware‐in‐the‐loop (HIL) inference using a 784−20−10 SBNN on a class‐balanced subset of 100 MNIST test images. A physical MTJ generates the stochastic neuronal outputs, while parameter storage and network arithmetic remain in software. Software and HIL inference both correctly classify 87 of the 100 images, with similar neuronal firing rates in the evaluated examples. Additional device measurements characterize switching repeatability and pulse‐width dependence, while variability simulations assess the sensitivity of inference to changes in switching characteristics. Together, these results support MTJ switching as a physical neuronal sampling primitive and motivate further integration of stochastic devices with probabilistic spiking networks.

## 2 Results and Discussion

The results comprise device characterization, software evaluations, and HIL validation. Section 2.1 presents the MTJ switching characteristics. The network performance, training‐efficiency, robustness, and uncertainty analyses in Sections 2.3, 2.3.1, 2.3.2, 2.3.3, 2.4, 2.5, 2.5.1, 2.5.2, 2.6 are evaluated in software. Section 2.7 reports HIL inference, in which physical MTJ switching events generate neuronal outputs while the remaining network computations are performed in software. Section 2.8 combines device‐repeatability measurements with numerical sensitivity analyses of device variability.

### 2.1 Stochastic Spiking Neuron Implemented with MTJ Devices

In this work, the stochastic spiking neuron is implemented using a MTJ, the fundamental building block of spintronic devices [47]. We established a mathematical relationship between the switching behavior of MTJ and the neuron dynamics of the stochastic leaky integrate‐and‐fire (LIF) model, which makes it possible for a device to work as a spiking neuron. The parallel (P) and anti‐parallel (AP) resistance states represent spike and silent events, respectively (Figure1A). Near the critical drive, MTJ switching is intrinsically stochastic due to thermal fluctuations, and the switching probability follows a sigmoid‐like cumulative distribution:

(1) $$P_{SW}\left(U\right)=\int_{-\infty }^{U}f_{MTJ}\left(v\right)dv.$$

Here, the $P_{SW}\left(U\right)$ and $f_{MTJ}\left(v\right)$ are the switching probability distribution and switching voltage probability density function of MTJ, respectively. A stochastic‐threshold LIF neuron admits an analogous probabilistic firing rule $P_{spike}\left(V\right)=\int_{-\infty }^{V}f_{th}\left(v\right)dv$, where the $P_{spike}\left(V\right)$ is the probability of neuron emitting a spike under the input V and the $f_{th}\left(V\right)$ indicates the probability density function of the spiking neuron threshold. By identifying $f_{th}\equiv f_{MTJ}$, a single MTJ directly realizes threshold sampling and stochastic spiking (see Methods 4.2 for derivation).

In our implementation, MTJs are reset to AP at the beginning of each timestep, leading to an effective memoryless spiking decision driven by the applied voltage (see Methods 4.9, 4.10, 4.11). Experimentally, the switching probability increases monotonically with pulse amplitude and is well captured by a sigmoid fit, yielding a characteristic voltage for 50% switching probability $U_{50}\approx 0.5V$ (Figure 2C, N=10000 trials per data point, Table S3). The extracted slope (or equivalent threshold spread) quantifies the effective stochastic‐threshold distribution. Under identical driving conditions, switching events exhibit inter‐experimental variability due to stochastic dynamics dominated by thermal noise (Figure 2D).

**FIGURE 2:**
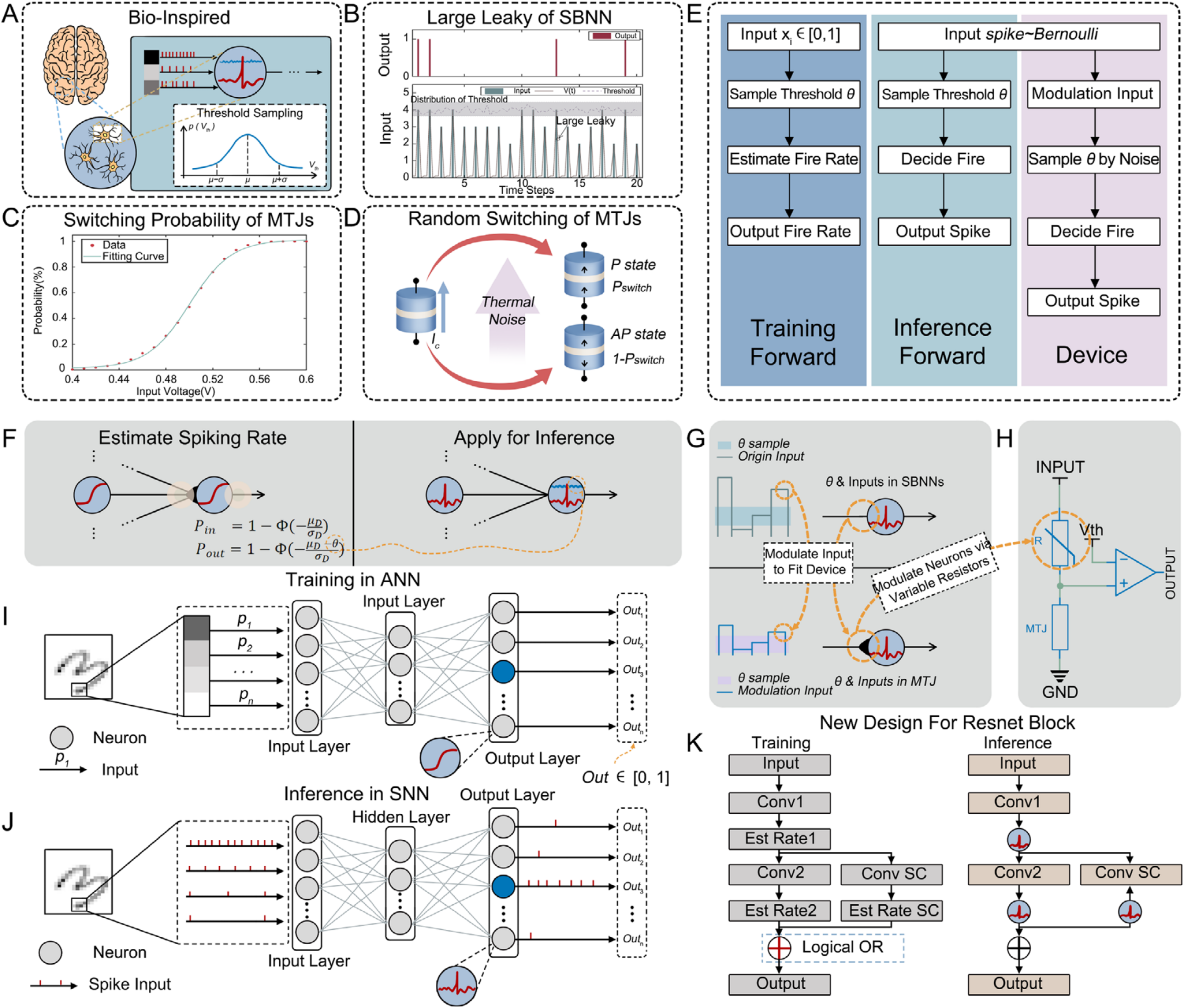
Spiking Bayesian Neural Network Implemented with MTJs. (A) Bio‐inspired Spiking Bayesian Neuron. Conceptual bio‐inspired spiking Bayesian neuron (SBN) with brain and neuron sketches, and stochastic threshold sampling from a distribution. (B) Membrane Dynamics. Time‐domain SBNN behavior showing that, identical inputs can produce different spike outputs. (C) MTJ Switching Probability. Experimentally measured probabilistic switching curve of a MTJ, showing a sigmoidal dependence of switching probability on the applied voltage, centered around 0.5V. (D) Stochastic MTJ State Transitions. Identical current inputs may yield different resistance states with characteristic probabilities. (E) Training, Inference, and Device‐Level Inference. The SBNN takes probability‐valued inputs during training and binary spikes during inference. The device branch illustrates input modulation for MTJ‐based neuronal evaluation. (F) Probabilistic SBN formulation. For a Gaussian pre‐activation with mean $\mu_{D}$ and standard deviation $\sigma_{D}$, the input and output spike probabilities are $P_{in}$ and $P_{out}$, where θ is the stochastic threshold, and Φ is the standard normal cumulative‐distribution function. (G) Input modulation. Algorithmic neuronal inputs are mapped to device drive voltages to align the neuronal firing midpoint with the MTJ switching midpoint. (H) Conceptual hardware neuron. A possible circuit uses a variable resistor to modulate the input to an MTJ‐based stochastic neuron. The HIL experiment implements this mapping in software using external measurement equipment. (I) Probability‐domain forward pass during training. Normalized pixel intensities $p_{n}\in \left[0,1\right]$ serve as input firing probabilities, and each neuron outputs an estimated firing probability. Here, $p_{n}$ denotes the normalized grayscale intensity of input pixel n. (J) Inference Forward Mode. The same parameters operate in an SBN‐based spiking model. Inputs are Bernoulli‐sampled spike events from pixel probabilities, producing binary spike outputs 0,1. (K) ResNet‐compatible block design. During training, branch fusion is performed using a logical‐OR operation (red 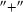), and during inference, it is replaced by linear residual addition (black 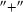).

### 2.2 SBNN Architecture and Rate‐Estimation Training

#### 2.2.1 Hardware‐Software Co‐Design for MTJ‐Based SBNN

Based on the proposed MTJ‐based stochastic neuron, we constructed a SBNN that realizes intrinsic threshold sampling, which works well even with low precision weights. In the proposed hardware–software co‐design framework, each neuron samples its threshold from a distribution parameterized by a mean and a variance (Figure 2A). Threshold sampling allows identical inputs to produce different spike outputs, as illustrated in Figure 2B. For the static‐image models, these parameters are optimized using the proposed RE method. By optimizing these parameters through variational inference, the network naturally encodes a Bayesian posterior distribution, enabling explainable probabilistic outputs. Figure 2E contrasts probability‐domain training, software spiking inference, and device‐based neuronal evaluation. Figure 2F illustrates the relationship between input statistics, neuronal thresholds, and firing probabilities. Figure 2G shows the input modulation used to map algorithmic neuronal inputs to MTJ drive signals, while Figure 2H presents a conceptual circuit for this modulation. In the HIL experiment, input mapping is performed in software, and the drive pulses are applied using the measurement system.

#### 2.2.2 Rate‐Estimation Training Method

Conventional SNNs are constructed either by direct SG learning or by converting pretrained ANNs [48, 49, 50]. However, SG methods rely on heuristic gradient approximations that often limit accuracy and convergence speed in deep architectures [51, 52], while conversion pipelines require extensive latency (hundreds of time steps) and post‐hoc calibration to match firing rates [2, 53]. To resolve these limitations, we developed a RE training framework (Figure 2I) that enables efficient, end‐to‐end optimization by treating neural dynamics as the propagation of probability distributions rather than discrete events. During training, RE computes layer‐wise firing probabilities directly in closed form by propagating spike distributions rather than point estimates (see “Rate‐Estimation Training Algorithm” in Methods), thereby avoiding explicit spike sampling and time‐step simulation. (Figure 2I). During software inference, the trained parameters are used to generate binary spike events (Figure 2J). To incorporate this formulation into residual architectures, we introduce the block in Figure 2K. It uses logical‐OR branch fusion during probability‐domain training and additive fusion during inference, enabling the proposed neuronal formulation to be evaluated within a residual backbone.

### 2.3 Accuracy and Calibration of SBNNs on Vision Recognition Tasks

To evaluate the performance of the proposed SBNN framework, we conducted supervised learning experiments on both static image benchmarks and event‐based dynamic vision benchmarks. The results are reported in two separate tables for clarity: Table 1 summarizes the results on static image datasets, including MNIST, Fashion‐MNIST, and CIFAR‐10, while Table 3 reports the results on event‐based datasets, including DVS Gesture, CIFAR10‐DVS, and N‐CALTECH101. Detailed preprocessing procedures, input encoding schemes, and training hyperparameters are provided in Notes S1 and S2 and Table S2.

**TABLE 1:** Performance of SBNN on Static Image Recognition Tasks. The proposed SBNN achieves competitive accuracy on MNIST, Fashion‐MNIST, and CIFAR‐10 under low‐precision settings and short inference latency.

Dataset	Type	Refs.	Hidden /Arch.	T ^a^	W / A (bits)^b^	Acc. (%)
MNIST	ANN	[54]	800	—	FP	98.66
			1200	—	FP	98.68
		[55]	1025×3	—	1/1	98.30
	SNN	[56]	1000	100	FP	97.91
		This work	1000	2	1/1	97.89
				4	1/1	98.32
				16	1/1	98.71
				2	8/1	98.80
				4	8/1	98.99
				16	8/1	99.16
F‐MNIST	ANN	[57]	1024×2	—	1/1	85.00
	SNN	[56]	1000	100	FP	89.05
		This work		2	1/1	83.48
				4	1/1	85.78
				16	1/1	87.31
				2	8/1	85.92
				4	8/1	88.38
				16	8/1	90.05
CIFAR‐10	ANN	[58]	ResNet‐20	—	1/32	91.20
	SNN	[59]	ResNet‐18	8	FP	94.82
		[60]	ResNet‐19	6	FP	95.40
		This work	ResNet‐19	2	Mixed^c^	94.64
				4	Mixed^c^	94.61
				8	Mixed^c^	94.84

a T denotes the number of inference time steps for SNN models.

b W/A denotes the bit‐width of weights and activations, respectively.

c In our implementation, ResNet‐19 consists of two basic ResNet blocks and one EstRate block. The basic blocks use full‐precision weights and activations, while the EstRate block and the final fully connected layer use 8‐bit weights and spiking neurons with 1‐bit activations.

**TABLE 2:** Threshold and Weight‐Precision Ablation on CIFAR‐10. Test accuracy (%) at the final checkpoint with T=8. Weight precision refers only to the final residual block of ResNet‐19; the remaining backbone configuration is unchanged.

Weight bits	Fixed^a^	Learned^b^	Random^c^	${\mathrm{SBNN}}_{\mu }$ ^d^	SBNN^e^ 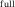
32	93.91	94.14	**94.17**	94.12	93.91
8	93.84	93.78	94.31	**94.48**	94.33
4	94.04	94.02	94.40	**94.41**	94.35
1	94.44	94.39	94.13	**94.53**	94.38

a Fixed: fixed deterministic thresholds.

b Learned: learnable deterministic thresholds.

c Random: thresholds are sampled from a fixed, non‐learned distribution at each time step during both training and inference, providing a threshold‐noise‐aware control.

d ${\mathrm{SBNN}}_{\mu }$: learns threshold means with fixed variances.

e ${\mathrm{SBNN}}_{\mathrm{full}}$: learns both means and variances. Reported weight bit widths apply only to the final residual block; the precision of the remaining network is unchanged.

**TABLE 3:** Performance of SBNN on Event‐Based Dynamic Vision Tasks. The proposed SBNN is further evaluated on event‐based datasets to verify its effectiveness on temporally sparse dynamic inputs.

Dataset	Refs.	Arch.	Type	T ^a^	$W_{\mathrm{params}}$ ^b^ (M)	Acc. (%)
DVS Gesture	[61]	ResNet	LIF	16	0.13	97.92
	[62]	GestureNet	PLIF	20	1.70	97.57
	Ours	GestureNet	SBN	20	1.70	97.22
CIFAR10 DVS	[63]	SpikFormer	LIF	16	2.57	80.9
	[64]	QKFormer	LIF	16	1.50	83.5
	Ours	SpikFormer	SBN	16	2.57	80.6
		QKFormer	SBN	16	1.50	82.8
N‐CALTECH 101	[65]	SWFormer	LIF	10	—	88.45
	[66]	QKFormer	LIF	16	1.53	87.24
	Ours	QKFormer	SBN	16	1.53	88.82

a T denotes the number of inference time steps for SNN models.

b $W_{\mathrm{params}}$ denotes the number of synaptic weight parameters, reported in millions.

For static image datasets, the SBNNs were trained using the proposed RE method, where the firing probabilities can be estimated from static input statistics. For event‐based dynamic datasets, however, accurate rate estimation becomes difficult due to the sparse and input‐dependent temporal spike streams. Therefore, we used SG training for these tasks to examine the applicability of the stochastic‐threshold SBNN model to dynamic inputs.

#### 2.3.1 Static Image Recognition

As shown in Table 1, the proposed SBNN achieves competitive performance on static image recognition tasks under both binary and low‐precision settings. Representative results are reported in the main table, while the complete accuracy results under different time steps and stochastic‐threshold parameters are provided in Table S1. On MNIST, the 8‐bit SBNN reaches 98.80%, 98.99%, and 99.16% accuracy at T=2, 4, and 16, respectively. Even under the fully binary configuration, the model achieves 98.71% accuracy at T=16. These results exceed the reported performance of Bayes‐by‐Backprop networks and a deeper binary neural network, while requiring only a small number of inference steps. Notably, the 8‐bit SBNN at T=4 already outperforms the full‐precision single‐spike SNN that requires 100 time steps, indicating that the proposed RE‐trained SBNN can achieve high accuracy with substantially reduced temporal latency.

On Fashion‐MNIST, SBNN also shows a clear accuracy improvement as the number of inference time steps increases. With binary weights and activations, the accuracy increases from 83.72% at T=2 to 87.31% at T=16. When the weight precision is increased to 8 bits, the accuracy further improves to 90.05% at T=16, surpassing the full‐precision single‐spike SNN baseline with 100 time steps. These results suggest that the proposed stochastic‐threshold mechanism effectively improves the expressive capability of low‐precision spiking neurons, especially when only a limited number of inference steps are available.

On CIFAR‐10, the mixed‐precision SBNN based on ResNet‐19 achieves 94.64%, 94.61%, and 94.84% accuracy at T=2, 4, and 8, respectively. The T=8 result is comparable to the full‐precision QCFS model and remains close to the IM‐Loss result, while using a mixed framework in which the Bayesian spiking block and the final classifier operate with low‐precision weights and binary activations. Compared with the binary ANN baseline BiPer, the proposed method provides a substantial accuracy improvement, demonstrating that SBNN can effectively narrow the performance gap between low‐precision SNNs and full‐precision models.

Table 2 isolates the effects of threshold configuration and local weight precision using the same ResNet−19 backbone. Five threshold configurations are compared at 32−, 8−, 4−, and 1−bit weight precision in the final residual block, with the remaining backbone configuration unchanged. Accuracy is evaluated at the final training checkpoint with T=8. At 8 bits, random thresholds achieve 94.31% accuracy, compared with 93.84% for fixed deterministic thresholds, while ${\mathrm{SBNN}}_{\mu }$ and ${\mathrm{SBNN}}_{\mathrm{full}}$ reach 94.48% and 94.33%, respectively. At 4 bits, the corresponding accuracies are 94.40%, 94.04%, 94.41%, and 94.35%. These comparisons show that threshold stochasticity can improve classification accuracy in the tested low‐precision block, with the largest observed gain over the fixed‐threshold baseline being 0.64 percentage points for ${\mathrm{SBNN}}_{\mu }$ at 8 bits.

The effect is precision‐dependent. At 1 bit, ${\mathrm{SBNN}}_{\mu }$ achieves 94.53%, whereas the fixed‐threshold, random‐threshold, and ${\mathrm{SBNN}}_{\mathrm{full}}$ configurations achieve 94.44%, 94.13%, and 94.38%, respectively. Across all four precisions, ${\mathrm{SBNN}}_{\mu }$ and ${\mathrm{SBNN}}_{\mathrm{full}}$ differ by at most 0.21 percentage points. Thus, learning the threshold mean while retaining a fixed variance preserves comparable classification accuracy in this configuration. Learning the variance does not provide a consistent additional accuracy gain. These results support stochastic‐threshold neurons as a locally quantized component within a mixed‐precision network.

#### 2.3.2 Event‐Based Dynamic Vision Recognition

Unlike static image datasets, event‐based dynamic inputs exhibit sparse and time‐varying spike distributions, making closed‐form rate estimation less straightforward across time. Therefore, for these dynamic vision tasks, we trained the SBNN models using SG learning rather than the proposed RE framework, in order to evaluate whether the stochastic‐threshold neuron can be effectively incorporated into temporally distributed event‐stream recognition. Table 3 further evaluates the proposed SBNN on event‐based dynamic vision datasets, including DVS Gesture, CIFAR10‐DVS, and N‐CALTECH101. Compared with static image inputs, event‐based inputs contain richer dynamic information and are naturally well matched to spiking computation. The proposed SBNN maintains competitive performance on these dynamic datasets, showing that the learned stochastic thresholds are not limited to static image classification but can also support event‐based recognition.

On DVS Gesture, the proposed SBNN achieves 97.22% accuracy with GestureNet at T=20. This result is close to the PLIF‐based GestureNet baseline (97.57%) and remains competitive with the ResNet‐based LIF model (97.92%), demonstrating that replacing deterministic spiking neurons with stochastic Bayesian neurons preserves strong gesture‐recognition capability on temporally sparse event streams. On CIFAR10‐DVS, we evaluated SBNN using both SpikFormer and QKFormer backbones. With SpikFormer, SBNN obtains 80.6% accuracy at T=16, which is comparable to the LIF‐based SpikFormer baseline (80.9%). With QKFormer, SBNN further improves the accuracy to 82.8% at T=16, approaching the LIF‐based QKFormer result (83.5%) and outperforming the original SpikFormer baseline. On N‐CALTECH101, the proposed SBNN achieves the strongest performance among the compared models, reaching 88.82% accuracy with QKFormer at T=16. This exceeds both the LIF‐based QKFormer baseline (87.24%) and the SWFormer result (88.45%).

Overall, the event‐based results show that the proposed stochastic‐threshold neuron is not limited to rate‐estimated static image recognition, but can also be incorporated into SG‐trained temporal SNN backbones for sparse event‐stream processing. In the proposed SBNN, intrinsic device stochasticity is transformed into a learnable neuronal‐threshold distribution. The learned threshold parameters encode neuron‐specific firing propensity and uncertainty, thereby acting as a compact neuron‐level memory that modulates spike generation during inference. This memory‐like stochastic threshold mechanism enhances the representation capability of spiking dynamics while remaining compatible with MTJ‐based physical sampling. Therefore, SBNNs provide a device‐aware route to robust and uncertainty‐aware neuromorphic computation across both frame‐based and event‐based recognition tasks. These results demonstrate compatibility with event‐based backbones.

#### 2.3.3 Accuracy and Calibration Against Bayesian Baselines

Table 4 compares classification accuracy, NLL, and ECE with reported Bayesian and ensemble baselines. On MNIST, the 8‐bit 128‐64 SBNN achieves 98.68% accuracy and an NLL of 0.044, compared with 98.00% and 0.062 for SUQ‐MFVI [67]. Its ECE is slightly higher, at 0.004 versus 0.003. The 400−400 and 800−800 SBNNs achieve accuracies of 99.07% and 99.17%, with NLLs of 0.032 and 0.031, respectively, improving both metrics relative to the listed baselines for those architectures. However, the floating‐point 128−64 SBNN has a higher NLL than SUQ‐MFVI.

**TABLE 4:** Performance comparison under different hidden‐unit settings on MNIST‐like dataset. Comparison of test accuracy (↑), negative log‐likelihood (NLL, ↓), and expected calibration error (ECE, ↓) for SBNN and Bayesian/ensemble baselines across multiple multilayer perceptron architectures. The compared baselines include SUQ‐MFVI [67], implicit generative‐prior BNNs [68], collapsed‐variational MF‐VI variants [69], and hyperparameter ensembles [70]. CM‐MF‐VI4k denotes CM‐MF‐VI trained with batch size 4000, while HDE and HBE denote hyper‐deep ensemble and hyper‐batch ensemble, respectively. Ours8bit and ${\mathrm{Ours}}_{float}$ denote SBNNs with 8‐bit synaptic weights and floating‐point weights, respectively.

Dataset	Hidden	Method	Acc.(%)↑	NLL↓	ECE↓
MNIST	128‐64	SUQ	98.00	0.062	**0.003**
		**Ours** 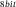	**98.68**	**0.044**	0.004
		${\mathrm{Ours}}_{float}$	98.01	0.069	0.007
	400‐400	VBNN	98.64	0.118	—
		SVBNN	98.60	0.144	—
		NA‐EB	98.76	0.057	—
		CM‐MF‐VI	98.66	0.047	—
		CV‐MF‐VI	97.87	0.068	—
		CMV‐MF‐VI	98.55	0.049	—
		CM‐MF‐${\mathrm{VI}}_{4k}$	98.69	0.041	—
		**Ours**	**99.07**	**0.032**	0.004
	800‐800	CM‐MF‐VI	98.58	0.048	—
		CV‐MF‐VI	97.91	0.066	—
		CMV‐MF‐VI	98.40	0.052	—
		CM‐MF‐${\mathrm{VI}}_{4k}$	98.80	0.042	—
		**Ours**	**99.17**	**0.031**	0.002
FMNIST	128‐64	SUQ	**89.10**	**0.313**	0.013
		${\mathrm{Ours}}_{8bit}$	88.66	0.322	**0.006**
		${\mathrm{Ours}}_{float}$	88.70	0.322	0.008
	200‐200	HDE	89.50	0.310	0.014
		HBE	89.20	**0.308**	0.016
		${\mathrm{Ours}}_{8bit}$	89.63	0.313	**0.007**
		${\mathrm{Ours}}_{float}$	**89.90**	0.313	0.009

On Fashion‐MNIST, the 128−64 SBNNs have lower ECE than SUQ‐MFVI but slightly lower accuracy and higher NLL. With 200‐200 hidden units, the floating‐point SBNN achieves 89.90% accuracy, while the 8‐bit variant achieves an ECE of 0.007. Both have an NLL of 0.313, slightly above the reported ensemble results. These comparisons show competitive probabilistic prediction with trade‐offs among accuracy, NLL, and ECE. The software evaluations assess the stochastic‐threshold model, while its physical sampling implementation is examined separately in Section 2.7.

Overall, Table 4 demonstrates that SBNN provides a device‐compatible route to Bayesian inference by modeling intrinsic stochasticity as learnable neuronal parameters. In contrast to conventional BNNs that usually introduce uncertainty through synaptic weights, our framework learns stochastic threshold distributions and samples them during inference, matching the physical behavior of stochastic MTJ neurons. The competitive accuracy, NLL, and ECE across MNIST and Fashion‐MNIST indicate that neuron‐level device randomness can serve as an effective Bayesian sampling source rather than an uncontrolled hardware non‐ideality. These results therefore reinforce the core motivation of SBNN by showing that intrinsic stochasticity in neuromorphic devices can support compact, low‐precision, and uncertainty‐aware probabilistic computation.

### 2.4 Efficient Training by Rate‐Estimation Method

We compared RE and SG training using the same 784−1000−10 SBNN architecture on MNIST. The upper panel of Figure 3A reports classification accuracy and training time per epoch for the tested time‐step settings. Both methods achieve closely matched accuracy, exceeding 99.0% from T=4 onward and reaching 99.22% at T=32. SG training time increases from 6.91 s per epoch at T=2 to 112.71 s at T=64, whereas RE training takes approximately 5.05 s per epoch. This yields roughly a 22‐fold speedup at 64 time steps while preserving accuracy, for the evaluated architecture and training setup. This speedup is an algorithmic benefit of propagating firing probabilities without explicitly unrolling temporal spike sequences.

**FIGURE 3:**
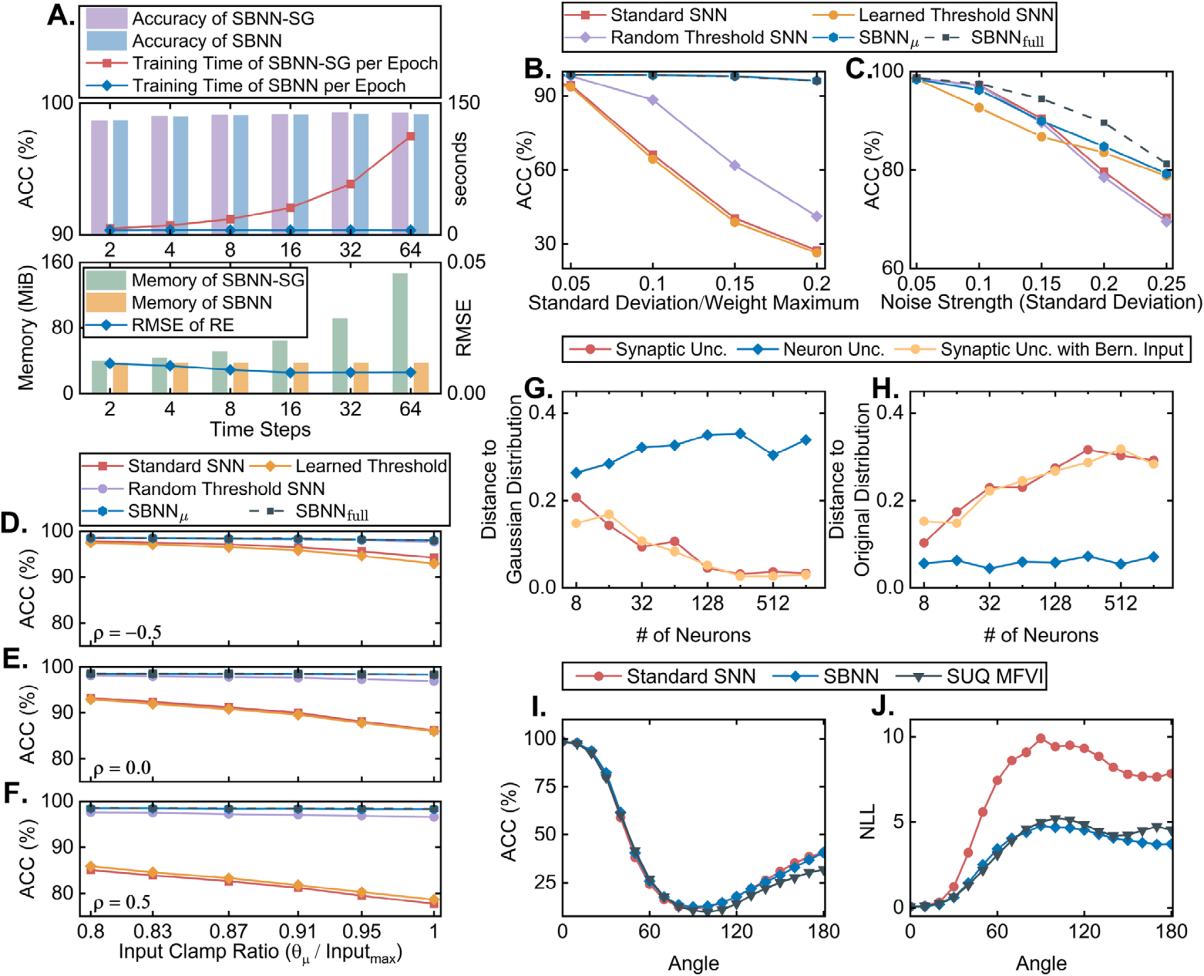
Efficient training, robustness, and distributional behavior of SBNNs. (A) Training efficiency and RE method error. Comparison of RE and SG training using a 784−1000−10 fully connected network on MNIST. Upper panel: bars indicate classification accuracy (left axis, %), and lines indicate wall‐clock training time per epoch (right axis, seconds). Lower panel: bars indicate peak memory usage for RE and SG training (left axis, MiB), and the blue line with diamond markers indicates the root‐mean‐square error (RMSE) between the rate‐based and spiking models (right axis). RE training time and peak memory usage remain approximately constant over the tested time steps, while the RMSE generally decreases as the number of time steps increases. (B–F) Robustness comparisons among the standard SNN, learnable deterministic‐threshold SNN, random‐threshold SNN, ${\mathrm{SBNN}}_{\mu }$, and ${\mathrm{SBNN}}_{\mathrm{full}}$, using the same network architecture, number of training epochs, and inference time steps. The random‐threshold control uses a fixed distribution with threshold resampling at each time step during training and inference, providing a threshold‐noise‐aware baseline. (B) Synthetic Gaussian weight perturbations applied during software inference. Noise strength is the perturbation standard deviation divided by the maximum absolute weight, and it is not a measured MTJ weight‐noise level. (C) Additive Gaussian input noise, with the horizontal axis indicating its standard deviation. (D–F) Robustness to threshold perturbations under input clipping, with σ=log(1+exp(ρ)) and ρ=−0.5, 0, and 0.5, respectively. The horizontal axis is $r_{s}=\mu /{\mathrm{Input}}_{\max}$, the reciprocal of the normalized input limit ${\mathrm{Input}}_{\max}/\mu$. Increasing $r_{s}$ reduces the maximum permitted input relative to μ. (G, H) Distributional comparison between synaptic and neuronal uncertainty. Wasserstein‐1 distances are measured between the standardized membrane‐potential distribution and the standard Gaussian distribution (G) or the corresponding standardized source distribution (H). “Unc.” and “Bern. Input” denote uncertainty and Bernoulli input, respectively. Representative membrane‐potential distributions are provided in Figures S1– S7. (I, J) Uncertainty behavior under rotated‐MNIST distribution shifts. Classification accuracy (I) and negative log‐likelihood (NLL, (J)) are reported as a function of rotation angle. SUQ‐LA and SUQ‐MFVI denote Bayesian baselines from ref. [67].

The lower panel of Figure 3A reports peak memory usage and the root‐mean‐square error (RMSE) between estimated and simulated firing rates. RE avoids storing an explicitly unrolled spike sequence during training, resulting in a weaker dependence of memory usage on the time‐step setting. The firing‐rate RMSE decreases overall as the simulation window increases, but a residual discrepancy remains. Increasing the number of time steps reduces temporal sampling error. But it does not eliminate errors introduced by the rate approximation. These results support RE as an efficient training approach for the evaluated static‐input setting. Event‐based models in this study use SG training.

### 2.5 Robustness to Noise and Hardware Constraints

We next evaluated whether the learned stochastic‐threshold formulation improves robustness under noisy and hardware‐constrained conditions. We considered three types of perturbations: synaptic‐weight noise, input noise, and neuronal‐threshold noise combined with input clipping. These settings emulate non‐idealities that may arise when SNNs are deployed on neuromorphic devices.

#### 2.5.1 SBNN Works Robustly in Noisy Environment

To distinguish the contributions of threshold learning and stochastic noise exposure, we compared five configurations, including standard SNN, SNN with learnable deterministic thresholds, random‐threshold SNN, ${\mathrm{SBNN}}_{\mu }$, and ${\mathrm{SBNN}}_{\mathrm{full}}$. All five robustness models used the same network architecture, number of training epochs, and inference time steps. In the random‐threshold SNN, the threshold distribution remains fixed and thresholds are resampled at every time step during both training and inference. This baseline therefore provides threshold‐noise‐aware training without learning the threshold distribution. ${\mathrm{SBNN}}_{\mu }$ learns the distribution means with fixed variances, whereas ${\mathrm{SBNN}}_{\mathrm{full}}$ learns both means and variances.

Under synaptic‐weight perturbations (Figure 3B), the two SBNN variants were substantially more robust than the deterministic and fixed‐distribution stochastic SNNs. At a normalized weight‐noise standard deviation of 0.20, accuracy was 27.42% for the standard SNN, 26.38% for the learnable deterministic‐threshold SNN, and 41.13% for the random‐threshold SNN. In comparison, ${\mathrm{SBNN}}_{\mu }$ and ${\mathrm{SBNN}}_{\mathrm{full}}$ retained 96.25% and 96.20% accuracy, corresponding to gains of 55.12 and 55.07 percentage points over the random‐threshold SNN. Their accuracies remained within 0.06 percentage points of each other over the tested noise levels. Thus, fixed‐distribution threshold noise provides partial protection, but does not reproduce the weight‐noise tolerance of the learned stochastic‐threshold models. Additional variance learning provides little further benefit for this perturbation.

For additive input noise (Figure 3C), the benefit of stochastic thresholds depended on the noise level. At a standard deviation of 0.10, the random‐threshold SNN achieved 92.69%, below the standard SNN at 97.20%, whereas ${\mathrm{SBNN}}_{\mathrm{full}}$ achieved 97.48%. At stronger noise, ${\mathrm{SBNN}}_{\mathrm{full}}$ retained 94.44%, 89.68%, and 81.30% accuracy at standard deviations of 0.15, 0.20, and 0.25, respectively, compared with 90.00%, 84.81%, and 79.27% for ${\mathrm{SBNN}}_{\mu }$. At 0.25, the standard, learnable deterministic‐threshold, and random‐threshold SNNs achieved 70.27%, 69.49%, and 78.76%, respectively. ${\mathrm{SBNN}}_{\mathrm{full}}$ therefore improved accuracy by 11.03 percentage points over the standard SNN and by 2.54 percentage points over the random‐threshold SNN. Unlike the weight‐noise test, these results show an additional benefit from learning the threshold variance under stronger input perturbations.

We also evaluated threshold perturbations under restricted neuronal input ranges (Figure 3D–F). We parameterize the maximum permitted neuronal input, (Inputmax), relative to the threshold mean (μ) using the dimensionless ratio ($r_{s}=\mu /\mathrm{Input}\max$). Thus, (${\mathrm{Input}}_{\max}=\mu /r_{s}$), and larger ($r_{s}$) corresponds to tighter input clipping. At the tightest clipping setting, $r_{s}$ = 1.00, and ρ = 0.50, the standard and learnable deterministic‐threshold SNNs achieved 77.73% and 78.67%, respectively. The random‐threshold SNN retained 96.59%, while ${\mathrm{SBNN}}_{\mu }$ and ${\mathrm{SBNN}}_{\mathrm{full}}$ achieved 98.34% and 98.36%. Across all tested clipping and ρ settings, accuracy ranged from 98.02% to 98.55% for ${\mathrm{SBNN}}_{\mu }$ and from 98.01% to 98.59% for ${\mathrm{SBNN}}_{\mathrm{full}}$. Fixed‐distribution noise exposure therefore accounts for a substantial part of this robustness, with learned stochastic thresholds providing a further improvement. The near‐identical performance of the two SBNN variants indicates that additional variance learning is not essential for this test.

Together, these ablation studies separate benefits already obtained from fixed‐distribution stochastic training from those associated with learning threshold distributions. The strongest additional benefit is observed under weight perturbations, whereas variance learning provides an extra advantage under stronger input noise. These accuracy results support the effectiveness of the learned stochastic‐threshold formulation under the tested perturbations.

#### 2.5.2 Performance of SBNNs in Compact Architectures

Here, we evaluated the performance of our SBNN in a parameter‐constrained environment. The trained tiny SBNN was evaluated on MNIST dataset. Table 5 reports the test accuracy for compact FCN with a single hidden layer of width x, all evaluated under the same 784−x−10 topology. We swept the hidden‐layer width over x∈{10,20,30,50,100} to examine the accuracy with different hidden layer width x in small networks, where performance is typically sensitive to both model size and weight precision. Importantly, all prior compact models in the Table 5 used 32‐bit weights, whereas our SBNN was evaluated with only 8‐bit weights. In other words, the robustness of our 10 time steps SBNN was tested under a tighter precision constraint. Despite this 4× reduction in weight precision, SBNN achieved consistently high accuracy across all hidden‐layer widths. In the most constrained setting (x=10), SBNN reaches 90.84%, essentially matching the 32‐bit Westby's model [71] at 91.67% and substantially outperforms Dariol's model [72] at 85.00%. As width increases, the accuracy of SBNN improved smoothly and monotonically, indicating that the model effectively benefits from increased capacity while remaining stable under low‐precision quantization. Compared to 32‐bit compact baselines, SBNN remains highly competitive even at larger widths. For example, at x=50, SBNN achieves 96.86%, which is close to SPDNN [73] (97.77%), and at x=100 SBNN attains 97.87% compared with SPDNN's 98.61%. While SPDNN yields the highest absolute accuracy at wider settings, SBNN achieves near the SOTA performance among compact models with significantly reduced numerical precision. This indicates that our model is suitable for edge computing hardware integration.

**TABLE 5:** SBNN Performance in Tiny Architecture. This table summarizes test accuracy on MNIST for networks with a single hidden layer of width x. We report results across hidden‐layer sizes from 10 to 100 neurons and compare SBNN with prior compact models under the same 784−x−10 topology. Hidden‐layer width modulates model capacity, allowing direct assessment of how accuracy scales with architectural size and numerical precision in small networks.

		# of Hidden Neurons				
Refs.	*W* ^a^	10	20	30	50	100
[71]	32	91.67%	93.66%	94.89%	95.68%	—
[72]		85.00%	—	—	—	—
[73]		89.87%	94.60%	96.41%	97.77%	98.61%
SNN		89.09%	93.78%	95.57%	97.05%	98.23%
This work	8	90.84%	93.49%	95.80%	96.86%	97.87%

a W denotes synaptic weight precision in bits.

### 2.6 Bayesian Uncertainty Estimation With SBNNs

To evaluate predictive calibration and sampling efficiency of SBNNs, we study uncertainty propagation in SBNNs and its implications for predictive reliability. On one hand, we separated neuronal from synaptic stochasticity and characterized the role of central limit theorem (CLT) behavior in shaping the distribution of membrane potentials. On the other hand, we measured how sampling efficiency trades off against predictive robustness and compared our SBNN to recent Bayesian baselines.

#### 2.6.1 Neuronal Uncertainty and Synaptic Uncertainty

To understand the differences in mechanisms between the threshold stochasticity of our SBNN and the weight stochasticity of standard BNNs, we analyzed the variations in the distributions of membrane potential at a single neuron. We first explored the variations induced by synaptic uncertainty. Under a standard Bayesian formulation, each synaptic weight is modeled as a deterministic mean plus an independent zero‐mean random perturbation. Consequently, the membrane potential, which is computed as the weighted sum of inputs, represents a summation of multiple independent random variables. By virtue of CLT, as the number of input connections (fan‐in) increases, the distribution of this membrane potential asymptotically converges to a Gaussian (see Methods 4.3 for derivation and the equivalent threshold‐noise representation). On the other hand, neuronal stochasticity can be viewed as directly imposing uncertainty on the membrane potential threshold, thereby avoiding this degeneration.

Using a truncated, skewed Lévy α‐stable distribution as a biologically motivated heavy‐tailed reference [26], we quantified the distributional deviation using the Wasserstein‐1 distance. Specifically, we computed $d_{N}=W_{1}\left(\mathrm{std}\left(x\right),N\left(0,1\right)\right)$ to measure the distance to a standard Gaussian distribution and $d_{L}=W_{1}\left(\mathrm{std}\left(x\right),r_{L}\right)$ to measure the distance to the reference Lévy α‐stable distribution, where std(x) denotes standardized membrane‐potential samples and $r_{L}$ denotes the standardized reference distribution.

As shown in Figure 3G–H, synaptic uncertainty progressively drives the membrane‐potential distribution toward Gaussian‐like behavior as the fan‐in increases. This effect remains visible even when the inputs follow Bernoulli spike statistics, indicating that input stochasticity does not fully preserve the target heavy‐tailed structure under synaptic uncertainty. By contrast, neuronal uncertainty maintains a larger deviation from the Gaussian reference and a smaller distance to the Lévy α‐stable reference across different fan‐in values. Representative membrane‐potential distributions for the various uncertainty‐injection cases are provided in the Supporting Information. These results suggest that neuronal stochasticity offers a more direct and scalable way to preserve non‐Gaussian uncertainty, which is consistent with the role of neuronal variability in biological probabilistic computation.

#### 2.6.2 Out‐of‐Distribution Performance of SBNN

We further evaluated predictive reliability under out‐of‐distribution shifts using rotated MNIST. As the rotation angle increases, the test distribution gradually deviates from the training distribution, providing a controlled setting to examine both classification accuracy and uncertainty calibration. As shown in Figure 3I–J, the proposed SBNN was compared with a deterministic SNN baseline and the MFVI baseline from SUQ [67]. Across rotated inputs, SBNN achieves higher accuracy and lower NLL than the SUQ‐MFVI baseline, indicating that neuronal threshold sampling improves both classification robustness and likelihood‐based calibration under distribution shifts. This result suggests that the proposed neuronal uncertainty is not only useful for in‐distribution predictive calibration but also provides a reliable Bayesian‐like uncertainty source for OOD inputs.

To extend the evaluation beyond rotated MNIST, we trained models (500 epochs) on Fashion‐MNIST and used MNIST as an out‐of‐distribution dataset, as is shown in Table 6. OOD detection was evaluated using predictive entropy, with higher entropy indicating a stronger OOD score. We report the native predictive probabilities of all models without validation‐based post‐hoc calibration. In particular, the validation‐based calibration step in the SUQ evaluation was omitted for this comparison. This protocol evaluates uncertainty before post‐processing and is not intended to reproduce the performance of the complete calibrated SUQ pipeline. SUQ‐MFVI serves as the primary variational‐inference reference.

**TABLE 6:** Native Predictive Performance and OOD Detection. Fashion‐MNIST and MNIST serve as the ID and OOD datasets, respectively. All predictions are evaluated without validation‐based post‐hoc calibration, including SUQ‐MFVI [67]. OOD AUROC uses predictive entropy as the detection score. Accuracy, ECE, and AUROC are expressed as percentages. Bold entries indicate the best observed value in each column.

		ID: Fashion‐MNIST			OOD: MNIST
Method	Type	Acc.↑	NLL↓	ECE↓	AUROC↑
SNN^a^	SNN	87.05	0.43	5.80	78.95
SNN^b^		86.92	0.44	6.02	78.02
SNN^c^		86.90	0.40	3.98	83.74
${\mathrm{SBNN}}_{\mu }$ ^d^		87.20	0.39	3.70	83.96
${\mathrm{SBNN}}_{\mathrm{full}}$ ^e^		87.25	0.39	3.67	**84.12**
SUQ‐MFVI	ANN	**89.06**	**0.31**	**1.21**	80.58

^a^ Fixed deterministic thresholds.

^b^ Learnable deterministic thresholds.

^c^ Fixed threshold distribution, with thresholds resampled at each time step during training and inference.

^d^ Learnable threshold means with fixed variances.

^e^ Learnable threshold means and variances.

Among the five threshold configurations, ${\mathrm{SBNN}}_{\mathrm{full}}$ achieved the highest observed entropy‐based OOD AUROC of 84.12%, compared with 78.95% for the standard SNN, 78.02% for the learnable deterministic‐threshold SNN, 83.74% for the random‐threshold SNN, and 83.96% for ${\mathrm{SBNN}}_{\mu }$. On the in‐distribution test set, ${\mathrm{SBNN}}_{\mathrm{full}}$ achieved 87.25% accuracy, an NLL of 0.39, and an ECE of 3.67%, compared with 87.05%, 0.43, and 5.80%, respectively, for the standard SNN. However, the random‐threshold control already achieved an OOD AUROC of 83.74%, and ${\mathrm{SBNN}}_{\mu }$ had slightly lower NLL than ${\mathrm{SBNN}}_{\mathrm{full}}$. Thus, fixed‐distribution threshold‐noise training accounts for much of the improvement, while learning the threshold distribution provides a modest further gain in OOD discrimination in this experiment. The comparison with SUQ also reveals different trade‐offs between in‐distribution predictive quality and OOD discrimination. ${\mathrm{SBNN}}_{\mathrm{full}}$ exceeded the OOD AUROC of SUQ‐MFVI by 3.54 percentage points, whereas SUQ‐MFVI achieved higher in‐distribution accuracy and lower NLL and ECE. These results therefore support improved native uncertainty behavior relative to the deterministic SNN controls under the evaluated dataset shift, rather than a uniform advantage over alternative Bayesian inference methods. The small differences among the stochastic‐threshold models should also be interpreted as observed differences rather than statistically established improvements.

#### 2.6.3 Calibration Performance of SBNNs Across Network Architectures

Here, we assess the calibration performance of our SBNN on MNIST classification tasks. All models were trained with 16 time steps. Because the proposed SBNN is able to produce one independent Monte Carlo sample per time step, in the inference stage, the number of time steps and the number of Monte Carlo (MC) runs can be balanced under a fixed sampling budget.

In Figure 4A we fixed the total sampling budget (time steps multiplies MC runs) to 320. Varying the number of time steps revealed a best performance trade‐off operating regime in which both predictive uncertainty and likelihood‐based calibration improved. As the number of time steps was reduced from 320 to 8, the negative log likelihood (NLL) decreased monotonically and reached its minimum of 0.03179 at 8 time steps, while the mean predictive entropy remained low at 0.07509. A similarly stable behavior was observed at 16 time steps, with an NLL of 0.04223 and entropy of 0.01497. In contrast, further reducing the time steps led to a sharp degradation in both NLL and entropy, while increasing the time steps caused a degrading NLL alone. These results indicate that, under a fixed sampling budget, excessively few time steps or few MC runs fail to capture the posterior induced predictive distribution, whereas an intermediate range yields lower NLL together with reduced classification entropy, consistent with improved calibration.

**FIGURE 4:**
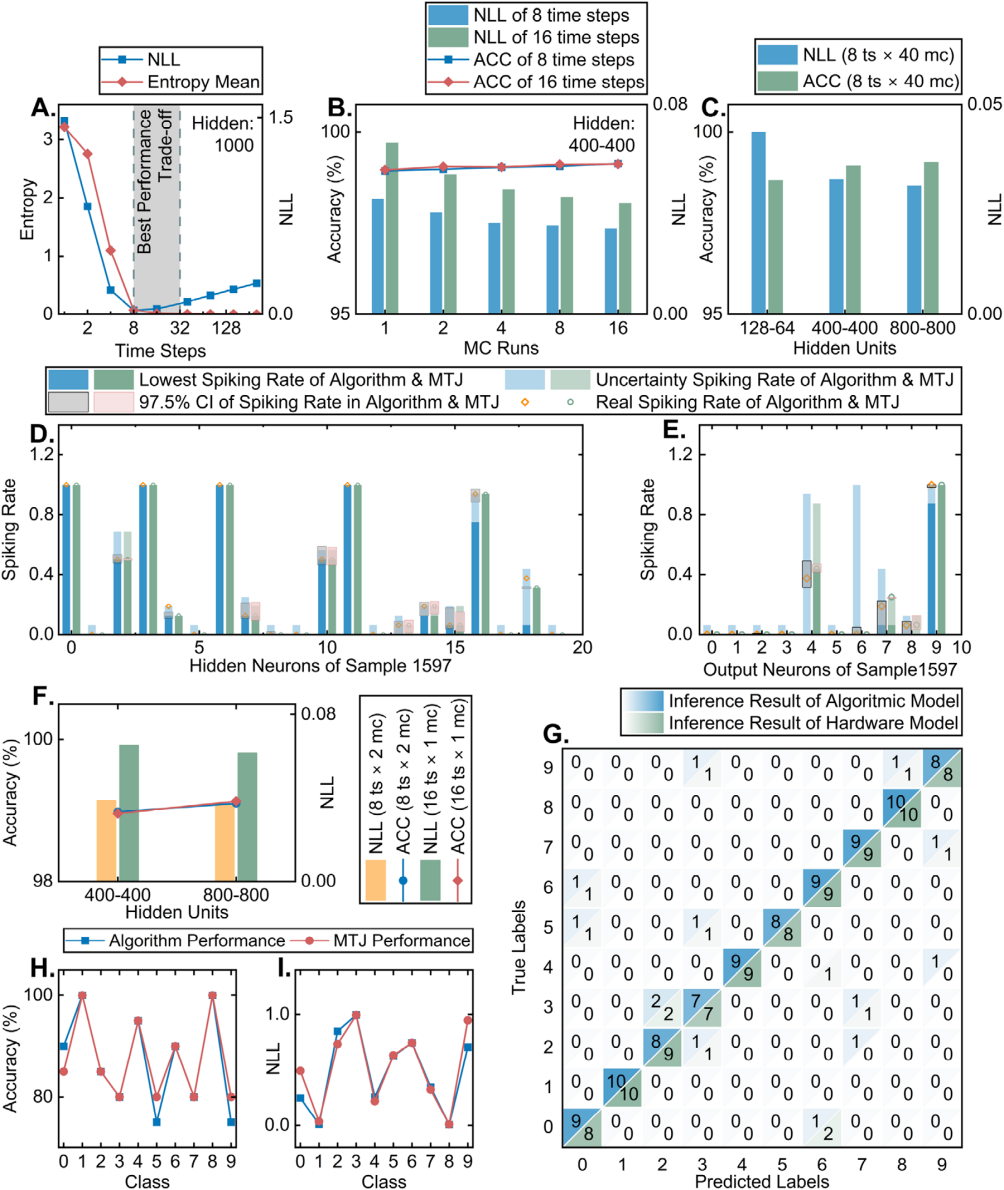
Calibration and Device Validation of Our SBNN on MNIST. (A) Under a fixed sampling budget of 320 total steps, varying the number of time steps reveals an optimal regime in which both NLL and mean predictive entropy are minimized, indicating improved calibration. Predictive entropy and NLL are plotted against the left and right axes, respectively. (B) On a 784−400−400−10 architecture, inference with 8 time steps matches the accuracy of 16 time steps while yielding lower NLL across various MC runs. Lines indicate accuracy on the left axis and bars indicate NLL on the right axis. (C) At 8 time steps, increasing hidden‐layer width improves accuracy and reduces NLL, and comparable calibration is obtained with as few as two MC runs relative to 40‐run averaging, consistent with efficient uncertainty estimation from independent samples per step. Green bars indicate accuracy on the left axis while blue bars indicate NLL on the right axis. (D) Hidden layer spiking rates for MNIST sample 1597 predicted by the algorithm and measured on MTJ hardware. Device rates were obtained by sweeping the input voltage. For each voltage, we pulsed the MTJ, inferred the post pulse state from the resistance, and counted switching events as spikes. The minimum spiking rate was estimated from voltages with switching probability above 99%. Uncertainty was quantified from all voltages with switching probability above 1%. Error bars denote 97.5% confidence intervals. (E) Output layer spiking rates for MNIST sample 1597 predicted by the algorithm and measured on MTJ hardware. Hidden layer spike activity was projected to the output layer using the learned weights to obtain each neuron input. After amplitude scaling, the corresponding value was applied as the MTJ input voltage and the device state was read out to estimate spiking rates. (F) Accuracy and NLL for two network architectures under a fixed total sampling budget of 16 steps, defined as the number of time steps per Monte Carlo run multiplied by the number of runs. Lines indicate accuracy on the left axis, and bars indicate NLL on the right axis. (G) Confusion matrix comparing the predictions of the software SBNN and the MTJ HIL implementation on the same test images. The trained parameters remained in software, and the mapped drive voltages were applied to the MTJ. The same test set was evaluated by the algorithm and by the device. (H) Per‐class accuracy of the software SBNN and MTJ HIL implementation using eight time steps per Monte Carlo run and two runs. (I) Per‐class NLL under the same sampling configuration. Panels (A–C) and (F) report software simulations, whereas panels (D), (E), and (G–I) compare software inference with HIL measurements. The HIL setup and inference procedure are described in Sections 4.10 and 4.11.

Figure 4B evaluated classification performance and calibration on a 784−400−400−10 architecture for models using 8 or 16 time steps in the best performance trade‐off operating regime. Across different MC runs, eight time steps achieved essentially the same accuracy as 16 time steps while consistently improving NLL. With 16 Monte Carlo runs, the 8‐time step model reached an accuracy of 99.13% and an NLL of 0.03274, while the 16‐time step model achieved an accuracy of 99.12% and an NLL of 0.04245. The same trend held at low sampling budgets. With a single Monte Carlo run, the 8‐time step model achieved an NLL of 0.04413 whereas the 16‐time step model yielded 0.0653. This comparison shows that reducing inference to 8 time steps preserves classification accuracy and improves likelihood‐based calibration.

In Figure 4C we studied the role of model size at 8 time steps and reported results averaging over 40 MC runs. Models with smaller hidden layers underperformed, as the 128−64 network achieved an accuracy of 98.68% with an NLL of 0.04354. Increasing capacity to 400−400 improved accuracy to 99.07% and reduced NLL to 0.0322, and the 800‐800 model further improved to 99.17% accuracy with an NLL of 0.0307. Notably, the 400−400 model retained comparable calibration even when the number of Monte Carlo runs was reduced from 40 to 2, achieving 99.01% accuracy with an NLL of 0.038. Given that each time step corresponds to an independent sample, this suggests that a compute budget comparable to roughly 16 samples can already yield high quality uncertainty estimates, enabling efficient inference without sacrificing probabilistic calibration.

### 2.7 Hardware‐in‐the‐Loop Validation of the SBNN Using MTJs

We evaluated the proposed SBNN using a HIL setup in which physical MTJ switching generated stochastic neuronal outputs, while network arithmetic and control were performed in software. An SBNN with a 784‐20‐10 multilayer perceptron architecture was trained entirely in software on the full MNIST training set. The HIL evaluation used a randomly selected, class‐balanced subset of 100 MNIST test images, with 10 images per class. The software and HIL evaluations used the same trained parameters and test images.

During HIL inference, the synaptic weights and neuronal threshold parameters remained stored in software. For each neuronal evaluation, software computed the weighted input and mapped it to an MTJ drive voltage according to Equation (2), where μ denotes the learned threshold mean and 0.4997 V is the measured reference voltage corresponding to 50% switching probability. The measurement system applied the corresponding pulse to the MTJ and read its post‐pulse resistance state to obtain a binary neuronal output. For this HIL experiment, the algorithmic threshold‐scale parameter was fixed at ρ=−2, corresponding to σ=log(1+exp(−2)), rather than optimized independently for each neuron. Equation (2) aligns switching midpoint of each neuron with the learned threshold mean using a common measured MTJ switching curve. It does not independently reproduce an arbitrary neuron‐specific threshold variance. The pulse‐width measurements in Figures S18– S20 demonstrate that the device switching distribution can be tuned, but both its center and width change with pulse duration. These measurements establish a possible tuning mechanism rather than a demonstrated procedure for independently programming all learned neuronal variances.

(2) $$x_{mod}\;=\;\frac{0.4997\;\cdot \;x_{in}}{\mu }$$

The HIL firing response is the measured device switching curve composed with the input modulation in Equation (2), and midpoint alignment does not independently match the algorithmic variance (Section 4.10). The measured hidden‐layer outputs were returned to software and combined with the learned weights to calculate the output‐layer inputs. Output‐layer stochastic events were obtained using the same MTJ measurement procedure. Input encoding, weighted summation, voltage mapping, output aggregation, and classification were performed in software. The experiment therefore validates physical stochastic spike generation within the network inference procedure, rather than a fully integrated hardware implementation of the SBNN. The measurement setup and inference procedure are detailed in Sections 4.10 and 4.11.

Figure 4D,E analyzes inference for an example sample (the 1597^th^ sample in MNIST test set which belongs to class 9). We plot the spike rate of each neuron in the hidden and output layers for both the algorithmic model and the MTJ based implementation. Individual spike times are not perfectly matched between the two realizations. In contrast the mean spike rates of corresponding neurons differ only slightly, and the minimum attainable rate of each neuron is identical in the two cases. The main visible difference is the width of the uncertainty bands for some neurons. These differences may reflect a mismatch between the learned threshold distributions and the effective distributions realized by the MTJ mapping. These results show similar mean firing behavior in the evaluated examples, despite differences in the probability‐curve widths. To further verify this behavior across different digit categories, additional representative samples from classes 0–9, representative error samples, and the complete hardware‐evaluated sample set are provided in Figures S8– S11. The corresponding neuron‐wise output spike rates for all hardware‐evaluated samples are listed in Tables S4– S8, enabling a direct quantitative comparison between the algorithmic model and the MTJ‐based implementation across all ten classes.

In previous sections, we found that although the models are trained in 16 time steps, their NLL performs better in 8 time steps. This discovery prompted us to investigate the splitting the inference of 16 independent spikes into two distinct 8‐time‐step MC samples, as is shown in Figure 4F. Across two different architectures, the accuracy of the 16‐time‐step model was very similar to that of the 8‐time‐step model, but the calibration performance (NLL) of the 8‐time‐step model was significantly better. We attributed this improvement in calibration performance to the benefits of twice mc samples. As a result, in the hardware implementation, we used 16 spikes for inference and separated them into two samples for calibration analysis.

Figure 4G compares the confusion matrices obtained from the algorithmic SBNN and from the MTJ hardware. Entries from the algorithm appear in the upper left half of each cell and entries from the hardware appear in the lower right half. These numbers represented the count of each possible prediction case (the predicted label in horizontal axis versus true label in vertical axis). The overall classification accuracy is identical for the two implementations. Most errors occur between visually similar classes which suggest that deployment on hardware introduces minimal additional degradation.

Figure 4H reports the class‐wise classification accuracy for the algorithmic SBNN and the MTJ‐based deployment, evaluated on 100 randomly selected MNIST test samples. The MTJ implementation closely tracks the algorithm across all classes. Small discrepancies are observed mainly for classes 0, 5, and 9, while the remaining classes show identical or nearly identical accuracy, indicating that mapping trained parameters to the device introduces minimal class‐dependent degradation.

Figure 4I compares the class‐wise NLL between the algorithmic model and the MTJ hardware, providing a calibration‐sensitive metric that reflects both prediction correctness and confidence. The overall NLL profile is largely preserved across classes, demonstrating similar uncertainty calibration after deployment. Modest NLL increases are visible for some classes (notably 0 and 9), consistent with the wider uncertainty bands observed for a subset of neurons in Figure 4D,E. The agreement on the misclassified samples suggests that the observed errors are largely associated with the trained model rather than additional errors introduced by the HIL mapping (as is shown in the Figure S11). Figures S21 and S22 directly compare the algorithmic firing‐probability curves with the common MTJ response after expressing them in the mapped voltage coordinate, for all 20 hidden neurons and 10 output neurons. The switching midpoints align, while the transition widths differ for some neurons, as expected from the mean‐only mapping. Figure S23 summarizes the corresponding neuron‐wise probability‐curve RMSE where the plotted values are below 0.15 for hidden neurons and below 0.20 for output neurons. These residual errors quantify mismatch in the firing responses rather than equivalence between a Gaussian threshold distribution and the fitted device distribution. Nevertheless, the software and HIL evaluations show similar task‐level behavior on the tested 100‐image MNIST subset. This supports tolerance to the measured mapping variation in this experiment, without establishing exact posterior sampling or equivalence over the full MNIST test set.

The practical consequence of this width mismatch is that midpoint alignment does not preserve all neuronal firing probabilities. Differences are particularly relevant near the switching transition, where a change in response width can alter spike probabilities and predictive confidence. Consequently, a preserved predicted class does not necessarily imply preserved likelihood or uncertainty calibration. Independent control of the threshold mean and variance would require additional circuit or operating parameters, or training that constrains the neuronal response to distributions physically available from the devices.

Taken together, Figure 4D,E,G–I shows close agreement between software and serial device‐in‐the‐loop inference on the evaluated 100‐image MNIST subset. A single physical MTJ was sequentially reused to generate stochastic neuronal outputs, while parameter storage, weighted summation, input mapping, and output processing remained in software. These results validate physical stochastic spike generation within a network inference procedure.

### 2.8 Robustness to Device‐to‐Device and Cycle‐to‐Cycle Variability

The HIL experiment validates stochastic neuronal operation using a single physical MTJ. To examine the sensitivity of the input‐mapping scheme to variability beyond this single‐device demonstration, we evaluated static device‐to‐device (D2D) differences and dynamic cycle‐to‐cycle (C2C) fluctuations in software. MTJ switching responses are represented by sigmoid probability curves, with variations in their centers and slopes modeling device variability. Stochastic outputs are then sampled from these probabilities, whereas HIL obtains the outputs from physical switching events. This framework allows us to assess the robustness of shared mapping and the potential benefit of additional device‐specific calibration.

For the static D2D analysis, we reconstructed normalized switching‐center and slope distributions from published switching‐curve histograms for 250 MTJs [74] and 144 MTJs [75], denoted as distributions 1 and 2, respectively (Figure 5A). These literature‐derived distributions provide experimentally informed variability ranges for the simulated device population. Because the source histograms report switching centers and slopes separately, their reconstruction uses an independent pairing approximation. The resulting normalized slope factors have geometric standard deviations (GSDs) of approximately 1.208 and 1.493 for distributions 1 and 2, respectively.

**FIGURE 5:**
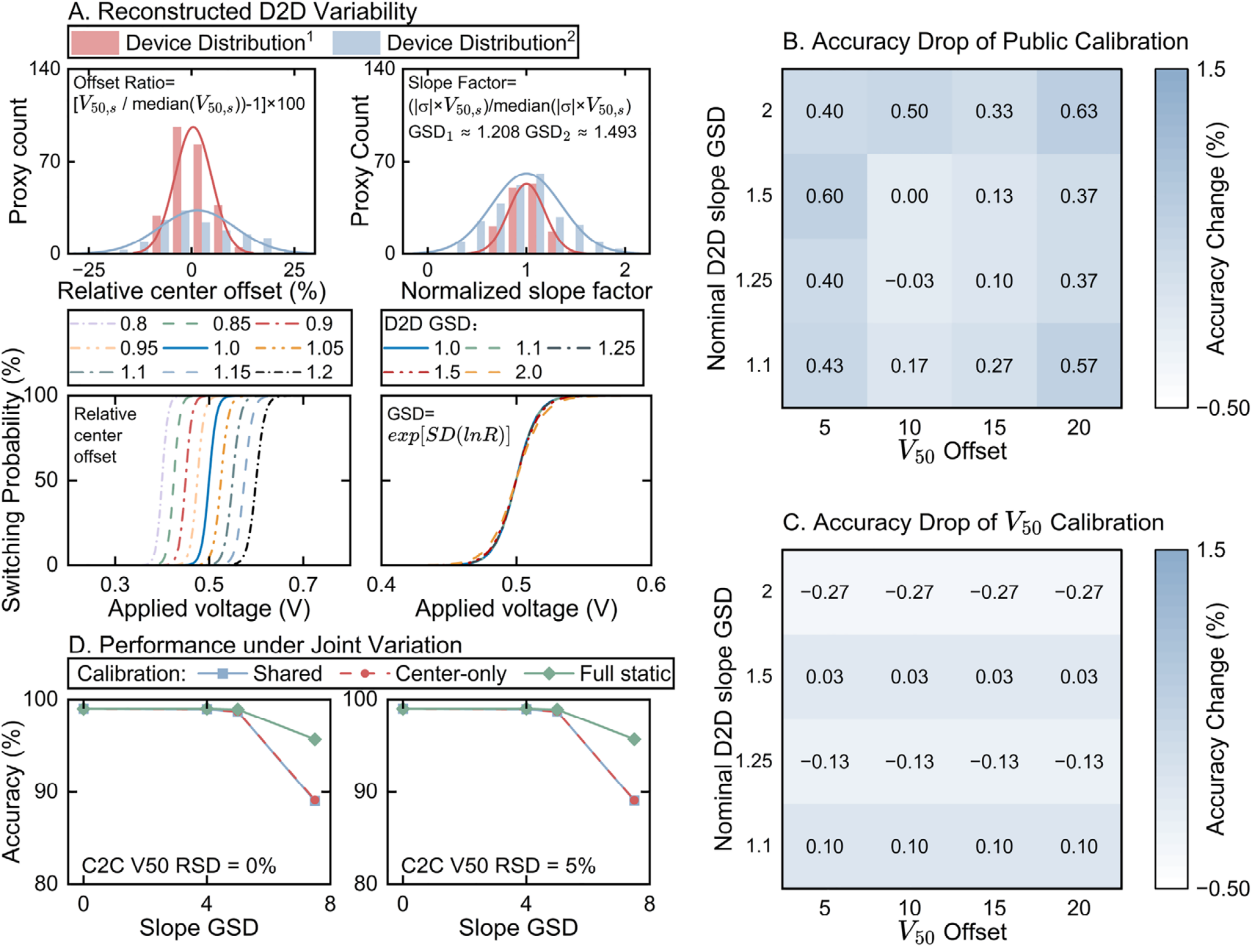
Sensitivity of SBNN inference to static and temporal MTJ variability. (A) Reconstructed relative switching‐center offsets and normalized slope factors obtained from published parameter histograms for 250 MTJs (distribution 1) [74] and 144 MTJs (distribution 2) [75]. The corresponding slope GSDs are approximately 1.208 and 1.493. Histogram counts represent reconstructed proxy records. The lower panels illustrate switching‐center shifts and representative switching curves for the indicated D2D slope dispersions. Corresponding illustrations of temporal slope variability on a D2D background are provided in Figure S17. GSD is defined as exp[SD(lnR)], where R is a positive slope multiplier. The representative curves use median slope multipliers rather than probabilities averaged over the multiplier distributions. (B,C) Mean accuracy change, in percentage points, relative to the no‐variation reference under shared mapping and center‐only calibration, respectively. The horizontal axis specifies the half‐range of the uniform relative D2D switching‐center offset, and the vertical axis specifies the nominal D2D slope GSD before clipping. Negative values indicate increased accuracy. (D) Accuracy under joint D2D and C2C variability for shared mapping, center‐only calibration, and full static calibration. Static D2D variability has a switching‐center offset range of 20% and a nominal slope GSD of 2.0. The C2C switching‐center relative standard deviation is 0% and 5% in the left and right panels, respectively, while the C2C slope GSD is varied. Full static calibration compensates static center and slope differences without tracking temporal fluctuations. Results are means over 30 paired realizations evaluated on the full MNIST test set with a fixed pretrained network at T=16.

We evaluated a fixed, pretrained 784–1000–10 SBNN with 8‐bit weights on the full MNIST test set at T=16, using 30 paired realizations of device variability and stochastic inference without retraining. Three mapping strategies were compared: a shared mapping based on the nominal switching curve, center‐only calibration using individual static switching centers, and full static calibration using both individual switching centers and slopes. Under the two reconstructed D2D distributions, different calibration strategy shared similar accuracies at around 99.01(±0.01, S.D.)%. These results indicate that, for the evaluated network and reconstructed variability ranges, using a shared mapping maintained classification accuracy without additional device‐specific center or slope corrections.

D2D switching center offset and slope sweeps further assessed sensitivity to simultaneous center and slope variations (Figure 5B,C). Switching‐center offsets were sampled within a relative range of 5%−20%, while the nominal slope GSD was varied from 1.1 to 2.0. Across these conditions, the magnitude of the mean accuracy change under the shared mapping remained below 0.7 percentage points relative to the no‐variation reference. Center‐only calibration removed the dependence on static center offsets in the implemented mapping model, while the remaining slope variation produced similarly small changes in accuracy. Here, nominal GSD denotes the dispersion specified before clipping the synthetic D2D slope factors and the reconstruction and sampling procedures are detailed in Experiments Setting and Methods (4.12).

To provide an experimental reference for the C2C variability analysis, we characterized the repeatability of the switching response in eight MTJs under fixed pulse‐width and temperature conditions. For each device, the measurements were divided into 100 consecutive windows, with 100 switching trials per voltage point in each window. The switching center, $V_{50}$, and the dimensionless slope factor, k, were extracted separately for each window. Across the eight devices, the within‐device coefficient of variation of $V_{50}$ ranged from 0.089% to 0.169%, while the geometric standard deviation of k ranged from 1.063 to 1.091 (Figure S16). These results indicate reproducible switching‐probability characteristics over the measured windows. The extracted dispersion includes finite‐trial fitting variability and therefore does not directly quantify switching‐parameter fluctuations at individual events. The observed repeatability is consistent with the stable switching‐curve parameters reported after more than $10^{10}$ switching pulses in a recent MTJ‐based probabilistic Ising machine [74], although our measurements do not constitute a comparable endurance test.

To assess inference sensitivity to temporal variability, we introduced switching‐center and slope fluctuations in separate single‐factor simulations. Under shared mapping without additional device‐specific correction, the mean MNIST accuracy was 99.018% for a relative switching‐center perturbation of $\sigma_{V}=5\%$, and 99.024% for a slope‐factor GSD of 1.20, compared with the nominal accuracy of 99.014%. The network therefore retained its classification performance under these simulated fluctuations. Although their numerical amplitudes exceed the measured window‐to‐window dispersion, the event‐wise simulation parameters and window‐wise experimental statistics describe different temporal scales. These simulations are thus sensitivity tests rather than direct reproductions of the measured temporal variability.

We next introduced C2C fluctuations on top of static D2D variability with a switching‐center offset half‐range of 20% and a nominal slope GSD of 2.0. Figure S17 illustrates representative switching curves for the temporal slope‐variability settings, while Figure 5D reports their impact on classification accuracy. With no C2C center fluctuations, increasing the C2C slope GSD from 4.0 to 7.5 reduced the mean accuracy of shared mapping from 98.97% to 89.07%, whereas full static calibration retained 95.70% accuracy at a C2C slope GSD of 7.5. Introducing a 5% relative standard deviation in the C2C switching center yielded similar accuracies under the tested conditions. Thus, the model tolerates the evaluated static variability, but sufficiently strong temporal fluctuations in the switching slope degrade inference accuracy. Full static calibration mitigates this degradation by compensating for static device differences; it does not track or compensate for individual C2C fluctuations.

Beyond variability at fixed operating conditions, we examined how pulse duration affects the switching response of MTJs with device widths of 200, 150, and 100nm (Figures S18– S20). Switching‐probability curves were acquired at pulse durations of 50ns, 100ns, 500ns, 1μs, 5μs, and 10μs, and fitted using binomial maximum likelihood estimation. The effective switching‐threshold voltage densities were obtained by differentiating the fitted probability curves with respect to voltage. Over the investigated conditions, longer pulses shifted the switching transition toward lower voltages and generally narrowed the effective threshold distribution. These results demonstrate pulse‐width‐dependent tuning of the switching response, with coupled changes in its center and width.

The variability simulations support shared mapping for the evaluated network and variability ranges. This mapping uses the learned neuronal threshold means and a common reference switching curve, without additional device‐specific correction coefficients. Center‐only and full static calibration require one and two additional static parameters per device, respectively (Section 4.12.3). The comparisons assume known static parameters, no drive‐voltage clipping and excludes resistance‐state readout errors.

Training for an actual device population would require measured switching‐probability curves under the intended pulse and temperature conditions. These responses could be incorporated into the training model, allowing weights and mapping parameters to adapt to the available device characteristics. Practical challenges include finite‐trial estimation uncertainty, correlations between switching centers and slopes, atypical devices, limited drive‐voltage ranges, readout errors, and drift. Characterization and correction also incur measurement and parameter‐storage costs. The independently paired literature‐derived distributions used here do not capture all of these effects.

For the simulated 784–1000–10 MNIST network, stochastic‐threshold training and distributed representations may contribute to the observed tolerance that moderate neuronal response changes need not reverse the output class ranking. Averaging across stochastic samples reduces sampling fluctuations, but does not by itself eliminate fixed D2D offsets. These explanations remain to be quantified separately. The approximately 99% accuracy therefore supports task‐level tolerance within the evaluated model and variability ranges, while measured‐array validation and assessment of predictive calibration remain necessary.

## 3 Conclusion

In this work, we reframed the stochastic switching in MTJs as a computational resource for neuromorphic computing, rather than an implementation error. We presented a co‐design architecture across hardware and software where intrinsic device randomness was modeled as neuronal uncertainty. In this framework, stochastic‐threshold neuron models are optimized through variational inference in software, allowing threshold distributions to be learned as part of the network. During inference, probabilistic computation emerges through sampling at the neuronal threshold. This formulation connects variationally learned stochastic thresholds in software with an approximate physical sampling primitive based on MTJ switching.

In software evaluations, SBNN achieves competitive performance across static and event‐based vision tasks. The reported configurations reach 99.16% accuracy on MNIST, 90.05% on Fashion‐MNIST, and 94.84% on CIFAR‐10, with the CIFAR‐10 result obtained using a mixed‐precision network. The same‐backbone CIFAR‐10 ablation further supports the use of stochastic thresholds in a locally quantized residual block, although the benefits depend on weight precision and do not establish a consistent advantage from learning threshold variance. For the evaluated MNIST fully connected network, RE training requires approximately 5.05 s per epoch, compared with 112.71 s for SG at T=64, corresponding to a speedup of about 22×. The event‐based models use SG training, and this speedup is not attributed to those event tasks.

The five‐model robustness comparison distinguishes fixed‐distribution threshold‐noise training from learned stochastic thresholds. At a normalized weight‐noise standard deviation of 0.20, SBNN retains 96.20% accuracy, compared with 41.13% for the random‐threshold SNN and 27.42% for the standard SNN. Additional variance learning provides a further benefit under stronger input perturbations, whereas its effect is small in the weight‐noise and clipping tests. These findings support the robustness of the learned stochastic‐threshold formulation under the evaluated perturbations without attributing all improvements exclusively to Bayesian inference.

Without post‐hoc calibration, SBNN achieves an entropy‐based OOD AUROC of 84.12% when Fashion‐MNIST is used as the ID dataset and MNIST as the OOD dataset, compared with 78.95% for the standard SNN and 83.74% for the random‐threshold SNN. It also achieves higher OOD AUROC than SUQ‐MFVI, while SUQ‐MFVI provides better ID predictive metrics. These results demonstrate useful native predictive uncertainty under the tested shift, with a modest additional benefit over fixed‐distribution stochastic training.

This design was inspired by evidence that biological circuits exhibit trial‐to‐trial variability, which relies on the neuronal intrinsic noise [24, 25]. Current neuroscience theories suggest that such variability supports probabilistic computation, even Bayesian inference, by enabling sampling‐based representations of latent uncertainty [29]. By assigning uncertainty to neuronal thresholds, our framework supports the exploration of diverse membrane‐potential distributions, including the non‐Gaussian distributions examined in this study. This provides a computational setting for investigating how neuronal variability contributes to probabilistic representations.

Implementing Bayesian inference/BNNs on conventional deterministic hardware often incurs substantial overhead due to sampling [76], while the neuromorphic hardware is implemented on the low precision systems to avoid errors [38], such as weight value drift [77]. The proposed SBNN achieves competitive accuracy against selected full‐precision baselines using low‐precision or mixed‐precision configurations. SBNN also retains competitive performance in compact networks with limited numbers of neurons and parameters. These results support the use of compact architectures and quantized weights to reduce parameter‐storage requirements, motivating deployment in resource‐constrained systems.

Beyond classification accuracy, the framework shows robustness to the tested perturbations in synaptic weights, input signals, and neuronal thresholds. Optimizing stochastic thresholds also supports stable inference under the tested input‐range constraints. This robustness extends to strong perturbations in weight values and neural activities, indicating that embracing noise during optimization can improve tolerance to the modeled perturbations that are otherwise difficult to control in emerging hardware. The additional D2D and C2C analyses show that shared mapping preserves classification accuracy within the evaluated variability ranges, while stronger temporal slope fluctuations identify a limit to this tolerance.

A central feature of this framework is the separation of neuronal sampling from synaptic‐weight precision. Many hardware‐oriented BNN implementations place stochasticity in synaptic weights, requiring the weight representation to support both storage and sampling. In a binary representation, the two available synaptic states constrain the direct encoding of a continuously distributed weight within a single synapse. Our SBNN shifts the stochastic source to the neuronal threshold, allowing probabilistic spike generation to coexist with deterministic, quantized synaptic weights. In a future integrated implementation, parameter storage, weighted summation, input encoding, voltage mapping, and output accumulation could all be implemented on chip. Training and initial device characterization could remain offline. The mapping in Equation (2) can be calculated during configuration and realized using a programmable gain stage or digital scaling followed by a digital‐to‐analog converter and pulse driver. MTJs would then generate local stochastic outputs, with reset and sensing circuits. The anticipated advantage is reduced random‐number generation and distribution overhead for neuronal sampling, while retaining deterministic quantized synaptic weights. Stochastic input encoding would still require a source of randomness.

The serial HIL experiment demonstrates that a single physical MTJ can generate stochastic neuronal events within an SBNN inference procedure, with classification accuracy and negative log likelihood (NLL) close to the software results on the evaluated 100‐image MNIST subset. Midpoint‐based input modulation aligns the neuronal threshold mean with the device switching midpoint, while the probability‐curve comparisons reveal residual distribution mismatch. The D2D simulations retain the same common input‐mapping principle and represent device‐specific switching responses using sigmoid probability curves. They provide complementary evidence that shared mapping can preserve classification accuracy within the evaluated variability ranges without additional device‐specific correction. This simulated tolerance supports the SBNNs further investigation of larger implementations.

The broader implication is that probabilistic neuromorphic systems can be built by embracing intrinsic device noise at the neuronal level. This architecture provides a novel method for injecting randomness that complements existing synaptic approaches. While current experiments focus on small to medium networks, the observed robustness motivates further evaluation in larger networks and integrated implementations. The mapping principle may also accommodate other stochastic devices whose switching statistics can be characterized and incorporated into the neuronal model. This flexibility implies that the approach is not limited to MTJs but can be generalized to other emerging technologies. Future work will explore combined sources of uncertainty in neurons and synapses, incorporate measured device responses into training, and evaluate integrated implementations in terms of energy, area, and latency.

## 4 Experiments Settings and Methods

### 4.1 Device Fabrication

The MTJ devices studied here were patterned from a (thermal silicon oxide substrate) $/Ta\left(5\right)$ $/CuN\left(20\right)$ $/Ta\left(5\right)$ $/{\left[Pt\left(2.5\right)/Co\left(1\right)/Ta\left(0.5\right)\right]}_{9}$ $/Pt\left(2.5\right)/Co\left(1\right)/Ta\left(1\right)$ $/{\mathrm{Co}}_{40}{\mathrm{Fe}}_{40}B_{20}\left(0.9\right)$ $/MgO\left(0.85\right)$ $/{\mathrm{Co}}_{20}{\mathrm{Fe}}_{60}B_{20}\left(1.1\right)$ $/Ta\left(0.5\right)$ $/Co\left(0.3\right)$ $/{\left[Pt\left(1.5\right)/Co\left(0.4\right)\right]}_{2}$ $/Ru\left(0.85\right)$ $/{\left[Co\left(0.5\right)/Pt\left(1.5\right)\right]}_{3}$ $/Ru\left(5\right)$ multilayer, deposited by using a Singulus ROTARlS physical vapor deposition system (thickness in nm). The 0.5nm thick Ta dusting layer was inserted between ${\mathrm{Co}}_{20}{\mathrm{Fe}}_{60}B_{20}andCo\left(0.3\right)/{\left[Pt\left(1.5\right)/Co\left(0.4\right)\right]}_{2}$, layers to enhance the perpendicular magnetic anisotropy (PMA) of the top reference layers. Here, a CuN layer was used as a buffer layer for the growth of the MTJ stack. The patterned electrodes, $Ti\left(10nm\right)$ and $Au\left(100nm\right)$ were fabricated using a lift‐off process.

### 4.2 MTJ‐based Stochastic Neuron Model and Bayesian Threshold Dynamics

In this work, the stochastic spiking neuron is implemented using a magnetic tunnel junction (MTJ), the fundamental building block of spintronic devices [11]. We established a mathematical relationship between the switching behavior of MTJ and the neuron dynamic of a stochastic leaky integrate‐and‐fire (LIF) model, which makes it possible for a device to work as a spiking neuron. As illustrated in Figure 1A, the MTJ comprises a pinned layer and a free layer separated by an oxide spacer, where the relative magnetic orientation determines the resistance state. When the magnetic moments of the two layers are in the same (opposite) direction, the MTJ is in the parallel (anti‐parallel) state and has a low (high) electrical resistance. Based on this correspondence, the parallel state can be associated with a spike event of the LIF spiking neuron model, and the anti‐parallel state can be regarded as the silent state where no spikes fire. The standard LIF model is defined by,

(3) $$\begin{array}{c} \tau_{m}\frac{dV\left(t\right)}{dt}=-\left(V\left(t\right)-V_{reset}\right)+R_{m}I\left(t\right)\\ S=H\left(V\left(t\right)-V_{th}\right)=1\left[V\left(t\right)\geq V_{th}\right] \end{array}$$

where $\tau_{m}$ is the membrane time constant, I(t) is the input current, V(t) is the membrane potential, and $V_{th}\mathrm{is}$ the threshold.

As shown in Figure 1B, switching between parallel and anti‐parallel states is driven by the spin‐torque and is inherently stochastic due to thermal fluctuations near the critical voltage $U_{C}$. Consequently, the switching probability $P_{sw}$ is governed by the cumulative distribution function (CDF) of the threshold voltage,

(4) $$P_{sw}\left(U(t)\right)=\int_{-\infty }^{U\left(t\right)}f_{MTJ}\left(v\right)dv=F_{MTJ}\left(U(t)\right)$$

where $f_{MTJ}\left(\cdot \right)\mathrm{and}$ $F_{MTJ}\left(\cdot \right)$ denote the probability density function (PDf) and CDF. In a deterministic LIF model, the firing probability $P_{det}$ is a step function, which can be written in the same integral form as Equation 4,

(5) $$P_{det}\left(V(t)\right)=\int_{-\infty }^{V\left(t\right)}\delta \left(v-V_{th}\right)dv=H\left(V\left(t\right)-V_{th}\right)$$

Here, δ(·)is the Dirac delta function and H(·)is the Heaviside step function. When introducing uncertainty to the threshold, the firing PDf changes from the Dirac function to a smooth PDf, and the firing probability evolves to a smooth CDF, consistent with the MTJ physics in Equation (4),

(6) $$P_{spike}\left(V(t)\right)=\int_{-\infty }^{V\left(t\right)}f_{th}\left(v\right)dv=F_{th}\left(V(t)\right)$$

where $f_{th}\left(\cdot \right)$ and $F_{th}\left(\cdot \right)$ are the PDf and CDF of the stochastic neuronal threshold. If we map the PDf of MTJ stochastic switching behavior, $f_{MTJ}\left(v\right)$, to the LIF threshold PDf, $f_{th}\left(v\right)$, a single MTJ can be viewed as a stochastic LIF neuron. Equivalently, the stochastic critical voltage for MTJ switching can be viewed as the stochastic membrane threshold of stochastic LIF neurons.

In our hardware implementation, the MTJs are set to the anti‐parallel state at the beginning of each time step, waiting for input from the previous layer (Figure 2B), which can be viewed as a large effective leakage so that the membrane potential does not retain memory of previous states ($\tau_{m}\to 0$). In this limit, the LIF state reduces to an instantaneous mapping from the applied drive to V(t). Using the applied MTJ voltage U(t) as the input drive (with proportionality absorbed into $R_{m}$) and the stochastic threshold ${\tilde{V}}_{th}$ from Equation (4), we obtain,

(7) $$V\left(t\right)\approx R_{m}U\left(t\right),\;\;S\left(t\right)=H\left(V\left(t\right)-{\tilde{V}}_{th}\right)$$

where ${\tilde{V}}_{th}$ follows the random distribution defined by $f_{MTJ}$. Experimental and simulation results show that the switching probability increases with the input current or voltage pulse amplitude with a sigmoid relation [78], as shown in Figure 2C, indicating a random threshold value $V_{C}$. In each switching attempt, $V_{C}$ is sampled from its underlying distribution. If the applied pulse amplitude surpasses the sampled $V_{C}$, the device switches state. Otherwise it remains in its original state. After each attempt, the device state is reset to the original state. The observed sigmoid switching curve therefore corresponds to the CDF of the threshold distribution, and its derivative gives the associated PDf.

### 4.3 Network Architecture and Noise Models

We consider a feedforward layer with input activations $S^{\left(l-1\right)}\in R^{n}$ and pre‐activations

(8) $$x_{j}^{l}=\sum_{i=1}^{n}w_{ij}^{l}S_{i}^{l-1}$$

Synaptic weights follow

(9) $$w_{ij}^{l}=\mu_{ij}^{l}+\varepsilon_{ij}^{l},\;E\left[\varepsilon_{ij}^{l}\right]=0,\;\mathrm{Var}\left(\varepsilon_{ij}^{l}\right)={\left(\sigma_{ij}^{l}\right)}^{2}$$

where the $\varepsilon_{ij}^{l}$ are mutually independent and independent of $S^{\left(l-1\right)}$. Substitution into Equation (8) yields

(10) $$x_{j}^{l}=\sum_{i=1}^{n}\mu_{ij}^{l}S_{i}^{l-1}+\sum_{i=1}^{n}\varepsilon_{ij}^{l}S_{i}^{l-1}$$

We define the mean and noise contributions

(11) $$M_{j}\left(x^{l}\right)\overset{def}{=}\sum_{i=1}^{n}\mu_{ij}^{l}S_{i}^{l-1},\;N_{j}\left(x^{l}\right)\overset{def}{=}\sum_{i=1}^{n}\varepsilon_{ij}^{l}S_{i}^{l-1}$$

By construction,

(12) $$E\left[N_{j}\left(x^{l}\right)|S^{l-1}\right]=0,\;E\left[x_{j}^{l}|S^{l-1}\right]=M_{j}\left(x^{l}\right)$$

and averaging over $S^{l-1}$ defines the neuron‐specific mean $m_{j}\overset{def}{=}E\left[M_{j}\left(x^{l}\right)\right]$.

Conditional on $S^{\left(l-1\right)}$, the terms

(13) $$X_{i}\overset{def}{=}\varepsilon_{ij}^{l}S_{i}^{l-1}$$

are independent, zero‐mean random variables with conditional variances

(14) $$\mathrm{Var}\left(X_{i}|S^{l-1}\right)={\left(S_{i}^{l-1}\right)}^{2}{\left(\sigma_{ij}^{l}\right)}^{2}$$

Assuming finite second moments and that no single term dominates the total variance, the Lindeberg–Feller central limit theorem implies that

(15) $$\frac{N_{j}\left(x^{l}\right)}{\sqrt{\sum_{i=1}^{n}{\left(S_{i}^{l-1}\right)}^{2}{\left(\sigma_{ij}^{l}\right)}^{2}}}\overset{d}{\to }N\left(0,1\right)\;\text{as}\;n\to \infty$$

Equivalently, for large n,

(16) $$N_{j}\left(x^{l}\right)|S^{l-1}\approx N\;\left(0,\;\sum_{i=1}^{n}{\left(S_{i}^{l-1}\right)}^{2}{\left(\sigma_{ij}^{l}\right)}^{2}\right)$$

To obtain a width‐independent effective variance, we adopt the usual wide‐network scaling

(17) $${\left(\sigma_{ij}^{l}\right)}^{2}=\frac{{\left({\tilde{\sigma }}_{ij}^{l}\right)}^{2}}{n}$$

with ${\tilde{\sigma }}_{ij}^{l}=O\left(1\right)$ and $E[{\left(S_{i}^{l-1}\right)}^{2}]$ bounded. Under these conditions, the sum in Equation (16) concentrates, by the law of large numbers, around a finite constant

(18) $$\sigma_{j,\mathrm{eff}}^{2}\overset{def}{=}\lim_{n\to \infty }\sum_{i=1}^{n}{\left(S_{i}^{l-1}\right)}^{2}{\left(\sigma_{ij}^{l}\right)}^{2}$$

and its relative fluctuations vanish as n→∞. Thus, in the wide‐network limit,

(19) $$x_{j}^{l}\approx m_{j}+\eta_{j},\;\eta_{j}∼N\left(0,\sigma_{j,\mathrm{eff}}^{2}\right)$$

providing a neuron‐wise Gaussian approximation to the aggregated synaptic noise.

We let the neuron output be

(20) $$S_{j}^{l}=\phi \left(x_{j}^{l}\right)$$

for a fixed activation function ϕ. Using Equation (19) into Equation (20) and defining ${\bar{x}}_{j}^{l}\overset{def}{=}m_{j}$ and $\theta_{j}\overset{def}{=}-\eta_{j}$, we obtain the equivalent representation

(21) $$S_{j}^{l}\approx \phi \left({\bar{x}}_{j}^{l}+\eta_{j}\right)=\phi \left({\bar{x}}_{j}^{l}-\theta_{j}\right)$$

Thus, in the wide‐network regime under the scaling of Equation (17), a model with microscopic synaptic weight noise is distributionally equivalent, at the level of neuron outputs, to a model with deterministic weights and a Gaussian prior on neuron‐specific thresholds $\theta_{j}$. Because $\theta_{j}$ is a single random variable per neuron and is not scaled with n, its variance does not diminish with increasing network width, and the resulting output stochasticity remains finite in the limit.

#### 4.3.1 Shape Metrics and Noise Families

Using a truncated, skewed Lévy α‐stable distribution as a biologically motivated heavy‐tailed reference [26], we quantify shape preservation by

(22) $$d_{ori}=W_{1}\left(std\left(x\right),r_{ori}\right)$$

and Gaussian by

(23) $$d_{N}=W_{1}\left(std\left(x\right),N\left(0,1\right)\right)$$

where $W_{1}\left(\cdot ,\cdot \right)$ denotes the Wasserstein‐1 distance, std(x) denotes standardized membrane‐potential samples, and $r_{ori}$ is the standard original distribution. We repeat the same analysis across various noise families, including Gaussian, exponential, gamma, symmetric Pareto, and Student‐t (Figures S1– S7).

### 4.4 Limited‐Precision Synaptic Weights

In the spiking‐Bayesian (SB) neuron layer, we employ low‐precision quantized synaptic weights, using either 1‐bit (binary) or 8‐bit representations depending on the experiment. The forward and backward procedures for the 8‐bit quantized weight module, QTsize8Weight, are illustrated in Figure S12. This quantization module is used not only for weight quantization, but also for calculating the mean and standard deviation of input spikes in the linear layer. For the quantized weights, 1‐bit and 8‐bit representations require 1/32 and 1/4 of the raw storage bits of 32‐bit representations, respectively, excluding auxiliary parameters and implementation overhead. These ratios describe weight‐storage requirements rather than measured energy savings. Binary and multi‐bit storage could be implemented using binary cells or bit‐sliced arrays in a future system. In the present HIL experiment, weights are stored and processed in software.

### 4.5 Rate‐Estimation Training Algorithm

During training, we operate purely in the rate domain, where the firing probabilities P at each layer are given by the outputs $\hat{p}$ of the previous layer, and no spike sampling is performed. For neuron i in layer l, the empirical output spiking rate over T discrete time steps is

(24) $$P_{out}=\frac{1}{T}\sum_{t=1}^{T}\vartheta \left(H_{t},\theta_{t}\right)$$

where $H_{t}$ is the total synaptic drive, θ is the (possibly time‐varying) threshold, and ϑ(·) is the Heaviside step function. Because the step is non‐differentiable, direct numerical optimisation of Equation (24) is intractable

In SBNNs, synaptic weights $w_{k}$ are 8‐bit valued and constrained to the interval [−1,1]. The pre‐activation value at each time step, D, to a neuron is modeled as a sum of the independent Bernoulli trials from each neuron in the previous layer multiplied by the corresponding weight value. Let $p_{k}$ be the Bernoulli probability that synapse k emits a spike in the current window. By assuming pre‐synaptic spikes are independent across synapses, the Lyapunov condition is satisfied and the net input D is well approximated by a normal random variable with mean $\mu_{D}$ and variance $\sigma_{D}^{2}$, derived analytically from the input probability P and synaptic weights W:

(25) $$\mu_{D}=\sum_{k}w_{k}p_{k},\;\;\sigma_{D}^{2}=\sum_{k}w_{k}^{2}p_{k}\left(1-p_{k}\right)$$

The Gaussian cumulative distribution function appearing in the definition of $P_{pos}$ has no elementary closed form, so we approximate it with a scaled logistic function Φ(z)≈σ(kz), where σ(·) is the logistic sigmoid and k≈1.716. This yields

(26) $$P_{pos}\approx \sigma \left(1.716\cdot \frac{\mu_{D}}{\sigma_{D}}\right)$$

and, more generally, for a neuron with mean threshold θ,

(27) $$P_{out}=Pr\left(D>\theta \right)\approx \sigma \left(1.716\cdot \frac{\mu_{D}-\theta }{\sigma_{D}+\varepsilon }\right)$$

where ε>0 is a small constant for numerical stability. These expressions provide closed‐form, differentiable rate estimates for every neuron without requiring a temporal simulation loop during training. The corresponding forward and backward implementations, including the encapsulated EstRateLinearFunction, the SigmoidProb function, and the pseudocode for RE, are provided in Figure S13. This formulation replaces the non‐differentiable spike‐generation step with a differentiable approximation to the firing probability, allowing the gradients to be computed analytically:

(28) $$\frac{\partial P_{out}}{\partial \mu_{D}}\approx \frac{kP_{out}\left(1-P_{out}\right)}{\sigma_{D}+\varepsilon },\;\;\frac{\partial P_{out}}{\partial \theta }=-\frac{\partial P_{out}}{\partial \mu_{D}}$$

These gradients are propagated to the weights W via the chain rule applied to Equations (24) and (25). As a result, SNNs with low‐precision synapses in $\left[-1,1\right]$ can be optimized end‐to‐end using standard stochastic gradient descent by replacing the discontinuous spike nonlinearity with the logistic surrogate above. After training, the learned parameters are used in software spiking inference. The input‐voltage mapping for physical MTJ evaluation is described separately in Section 4.10.

#### 4.5.1 Rate‐Based Convolutional Estimator for Spiking Neurons

We model the pre‐threshold membrane current at each output location as a weighted sum of independent Bernoulli inputs. Let $P\in R^{B\times C_{in}\times H\times W}$ denote the input firing probabilities, $W\in R^{C_{out}\times C_{in}\times k_{H}\times k_{W}}$ the convolution kernels, and $\theta \in R^{C_{out}\times H_{out}\times W_{out}}$ a (possibly) spatially varying threshold. Under the independence assumption, the total current Dover a receptive field has mean and variance,

(29) $$\mu_{D}=P*W,\;\;\sigma_{D}^{2}=\left(P⊙\left(1-P\right)\right)*\left(W⊙W\right)$$

where * denotes convolution and ⊙ denotes elementwise (Hadamard) multiplication. For sufficiently many inputs, the central limit theorem implies that the standardized current is approximately Gaussian, and the firing probability can be written as

(30) $$\mathrm{Pr}\left(D>\theta \right)=\Phi \left(\frac{\mu_{D}-\theta }{\sigma_{D}+\varepsilon }\right)$$

where Φ is the Gaussian cumulative distribution function.

In practice, we approximate this probit nonlinearity with a scaled logistic (probit‐logit approximation) and use

(31) $$\hat{p}=\sigma \left(k\cdot \frac{\mu_{D}-\theta }{\sigma_{D}+\varepsilon }\right)$$

where σ(·) is the logistic sigmoid, k≈1.716 is a fixed scaling factor, and ε>0 is a small constant for numerical stability. For numerical stability, we treat $\sigma_{D}$ as a non‐trained value, which leads to the following approximate derivatives:

(32) $$\frac{\partial \hat{p}}{\partial \mu_{D}}\approx \frac{k}{\sigma_{D}+\varepsilon }\hat{p}\left(1-\hat{p}\right),\;\;\frac{\partial \hat{p}}{\partial \theta }\approx -\frac{k}{\sigma_{D}+\varepsilon }\hat{p}\left(1-\hat{p}\right)$$

At test time, the learned parameters (W,θ) are transferred to a multi‐timestep SNN with the same convolutional structure. As the number of simulation steps T and the number of input spikes S increase, the empirical firing rate of the spiking network converges to $\hat{p}$, ensuring consistency between the trained rate model and the deployed spiking dynamics.

### 4.6 Bayesian Interpretation of the SBNN Forward

For a spiking Bayesian (SB) neuron, we consider the instantaneous input current at a single time step

(33) $$D=\sum_{i=1}^{n}w_{i}X_{i},\;\;X_{i}∼\mathrm{Bernoulli}\left(x_{i}\right)$$

where $w_{i}\in \left[-1,1\right]$ are quantized synaptic weights and $x_{i}\in \left[0,1\right]$ denotes the presynaptic firing probability. In our implementation, $w_{i}$ is represented with b‐bit precision (we use 1‐bit, i.e. binary $w_{i}\in \pm 1$, and 8‐bit settings in the experiments, respectively).

Conditioned on the input probability vector $x=\left(x_{1},\text{…},x_{n}\right)$, the mean and variance of D are

(34) $$\mu_{D}=E\left[D|x\right]=\sum_{i}w_{i}x_{i},\;\;\sigma_{D}^{2}=\mathrm{Var}\left(D\;|\;x\right)=\sum_{i}w_{i}^{2}x_{i}\left(1-x_{i}\right)$$

A spike is emitted when the current exceeds a stochastic firing threshold θ,

(35) $$S=1\left[D\geq \theta \right]$$

We denote by $q_{\phi }\left(\theta \right)$ a variational distribution over thresholds, with cumulative distribution function $F_{q_{\phi }}$. Approximating the input by its conditional mean $D\left(x\right)=\mu_{D}$, the single step firing probability is:

(36) $$\begin{array}{c} \begin{array}{cc} p_{\mathrm{out}}\left(x\right) & =\mathrm{Pr}\left(S=1|x\right)\\ & =E_{\theta ∼q_{\phi }}\left[1\left[D\left(x\right)\geq \theta \right]\right]\\ & =\int_{-\infty }^{D\left(x\right)}q_{\phi }\left(\theta \right)d\theta \\ & =F_{q_{\phi }}\left(D\left(x\right)\right) \end{array} \end{array}$$

This expression shows that the neuron's firing rate is equal to the probability mass assigned by $q_{\phi }$ to thresholds less than or equal to the effective input current $D\left(x\right)$. In other words, the neuron implements a probabilistic threshold test whose uncertainty is governed by $q_{\phi }\left(\theta \right)$.

#### 4.6.1 Bayesian Firing Probability

Assume the threshold distribution $q_{\phi }\left(\theta \right)$ is trained variationally to approximate the Bayesian posterior p(θ∣D), $\mathrm{where}D={\left(x_{i},y_{i}\right)}_{i=1}^{N}$ is the training dataset and

(37) p(θ∣D)∝p(D∣θ)p(θ)

In the ideal variational limit $q_{\phi }\left(\theta \right)\to p\left(\theta |D\right)$, the firing probability for a new input x converges to the Bayesian posterior predictive probability,

(38) $$\begin{array}{c} p_{\mathrm{out}}\left(x\right)⟶\mathrm{Pr}\left(S=1|x,D\right)=\int \mathrm{Pr}\left(S=1|x,\theta \right)p\left(\theta |D\right)d\theta \end{array}$$

Since $\mathrm{Pr}\left(S=1|x,\theta \right)=\mathrm{Pr}\left(D\left(x\right)\geq \theta \right)$, the spike emission event can be viewed as a Bayesian decision under parameter uncertainty encoded by the posterior over θ.

#### 4.6.2 A Time Step as A Monte Carlo Sample

The membrane dynamics of a leaky integrate‐and‐fire (LIF) neuron can be written as:

(39) $$\begin{array}{c} \left\{\begin{array}{cc} Charge: & H_{t}={\left[V_{t-1}-\lambda \right]}^{+}+X_{in}\left(t\right)\\ Fire: & S_{t}=\vartheta \left(H_{t},\theta_{t}\right)\\ Reset: & V_{t}=V_{t-1}\left(1-S_{t}\right) \end{array}\right. \end{array}$$

where ${\left[z\right]}^{+}=\max\left(0,z\right)$, λ≥0 is the leak, $X_{\mathrm{in}}\left(t\right)$ is the synaptic input at time t, and $\theta_{t}$ is the firing threshold at time t.

Inspired by the dynamics of LIF neurons and by the reset behavior of MTJs, we instantiate SB neurons with a memoryless membrane potential. Concretely, we choose a very strong leak such that $\lambda \gg V_{max}$, where $V_{max}$ bounds the membrane potential in the operating regime. In this setting, the contribution from the previous state is negligible, so that

(40) $${\left[V_{t-1}-\lambda \right]}^{+}=0$$

and the pre‐threshold potential simplifies to

(41) $$H_{t}=X_{\mathrm{in}}\left(t\right),\;\;\theta_{t}\;\overset{i.i.d.}{∼}q\phi$$

Under rate encoding, the input $X_{\mathrm{in}}\left(t\right)$ is independent across time steps, with statistics determined by x. Together with the independent and identically distributed (i.i.d.) thresholds $\theta_{t}$, this implies that the tuples $\left(X_{\mathrm{in}}(t\right),\theta_{t})$ and the resulting spikes $S_{t}$ are independent and identically distributed, with

(42) $$E\left[S_{t}\right]=p_{\mathrm{out}}\left(x\right)$$

The empirical firing rate over T time steps,

(43) $${\hat{p}}_{T}\left(x\right)=\frac{1}{T}\sum_{t=1}^{T}S_{t}$$

is therefore an unbiased Monte Carlo (MC) estimator of $p_{\mathrm{out}}\left(x\right)$, with variance

(44) $$\mathrm{Var}\left[{\hat{p}}_{T}\left(x\right)\right]=\frac{p_{\mathrm{out}}\left(x\right)\cdot \left(1-p_{\mathrm{out}}\left(x\right)\right)}{T}$$

By the strong law of large numbers,

(45) $${\hat{p}}_{T}\left(x\right)\underset{T\to \infty }{\overset{a.s.}{⟶}}p_{out}\left(x\right)$$

Each simulation step can thus be interpreted as a MC sample from the Spiking Bayesian neuron, and averaging over T steps reduce the estimation variance at the usual $O\left(T^{-1}\right)$ rate.

### 4.7 Backpropagation and Bayesian Parameter Updates in SBNNs

Consider a three‐layer multilayer perceptron (MLP) implemented as a Spiking Bayesian Neural Network (SBNN). Using the RE approximation, the deterministic forward propagation is written as

(46) $$\left\{\begin{array}{cc} a_{0}=X & \\ z_{\ell }=k\frac{\mu_{a_{\ell -1}}-\theta_{\ell }}{\sigma_{a_{\ell -1}}} & \\ a_{\ell }=\sigma \left(z_{\ell }\right), & \ell \in \{1,2,3\} \end{array}\right.$$

where k≈1.716 is the slope of the logistic approximation, $\sigma \left(\cdot \right)$ denotes the logistic sigmoid, and $\mu_{a_{\ell -1}}$ and $\sigma_{a_{\ell -1}}$ are the mean and standard deviation of the input of each neuron at layer ℓ. These statistics are differentiable functions of the underlying real‐valued synaptic parameters and the inputs, obtained after binarisation of the synapses and masking of inactive inputs. The real‐valued parameters are projected to quantized weights (1‐bit or 8‐bit) via a straight‐through estimator, so that gradients can be propagated through the binarisation step.

Let $L=L(a_{3},Y)$ be the task loss. The local sensitivity at layer ℓ is defined as

(47) $$\delta_{\ell }=\frac{\partial L}{\partial z_{\ell }}$$

Since

(48) $$a_{\ell }=\sigma \left(z_{\ell }\right)$$

the chain rule gives

(49) $$\delta_{\ell }=\frac{\partial L}{\partial a_{\ell }}⊙a_{\ell }\left(1-a_{\ell }\right)$$

where ⊙ denotes element‐wise multiplication.

Using the definition of $z_{\ell }$, the partial derivatives of the loss with respect to the mean, standard deviation, and threshold terms are

(50) $$\left\{\begin{array}{c} \begin{array}{cc} \frac{\partial L}{\partial \mu_{a_{\ell -1}}} & =\delta_{\ell }⊙\frac{k}{\sigma_{a_{\ell -1}}}\\ \frac{\partial L}{\partial \sigma_{a_{\ell -1}}} & =\delta_{\ell }⊙\left(-k\frac{\mu_{a_{\ell -1}}-\theta_{\ell }}{\sigma_{a_{\ell -1}}^{2}}\right)\\ \frac{\partial L}{\partial \theta_{\ell }} & =-\sum_{b}\delta_{\ell ,:,b}⊙\frac{k}{\sigma_{a_{\ell -1},:,b}} \end{array} \end{array}\right.$$

where the index b runs over the batch, and : denotes all units in the layer. For numerical stability, elements with very small $\sigma_{a_{\ell -1}}$ can be treated as effectively deterministic, which are reduced to a hard threshold in the forward path, and the corresponding gradients are suppressed.

The quantities $\mu_{a_{\ell -1}}$ and $\sigma_{a_{\ell -1}}$ are themselves differentiable functions of the synaptic weights and the inputs, typically obtained through linear combinations of masked inputs and simple nonlinear transformations. Gradients with respect to the underlying real‐valued weights are then computed automatically by backpropagation through these operations, together with the straight‐through approximation used in the binarization step.

#### 4.7.1 Threshold Parameterization and Sampling

Each neuronal threshold is assigned a Gaussian variational posterior. For a given threshold with parameters μ and ρ, the corresponding standard deviation is defined as

(51) $$\sigma_{\theta }=\log\left(1+e^{\rho }\right)$$

and a sample from the posterior is obtained through the reparameterization

(52) $$\tilde{\theta }=\mu +\sigma_{\theta }\cdot \varepsilon ,\;\;\;\varepsilon ∼N\left(0,1\right)$$

To prevent collapse of thresholds toward zero and to avoid excessively excitable neurons, a positive lower bound $\theta_{min}>0$ is enforced by clamping,

(53) $$\theta =\max\left(\tilde{\theta },\theta_{min}\right)$$

This truncated sampling procedure is used both in the forward pass, where the threshold sample determines the firing rate, and in the backward pass, where gradients are propagated from θ back to μ and ρ via the reparameterization. The corresponding forward and backward procedures for SampleThreshold are illustrated in Figure S14.

In practice, several independent samples of $\tilde{\theta }$ are drawn for each neuron. After clamping, the mean of these samples is used as the effective threshold in the forward computation, while the same samples are reused to construct a MC estimate of the Kullback–Leibler (KL) divergence between the variational posterior and the prior. The encapsulated VariationalThreshold module, which performs threshold sampling and KL‐divergence calculation during each forward pass, is summarized in Figure S15.

### 4.8 Bayesian Treatment of Threshold Uncertainty

Threshold uncertainty is treated in a Bayesian manner using variational inference in the spirit of Bayes‐by‐Backprop. Let

(54) $$q\left(\theta |\mu ,\rho \right)$$

denote the approximate posterior over thresholds, with standard deviation Equation (51). For a dataset $D={\left(x_{i},y_{i}\right)}_{i=1}^{N}$, the variational free energy (negative evidence lower bound) is

(55) $$\begin{array}{c} F\left(D|\mu ,\rho \right)=E_{q}\left[\log q(\theta |\mu ,\rho )\right]-E_{q}\left[\log p(\theta )\right]-E_{q}\left[\log p(D|\theta )\right]\;\;\;\; \end{array}$$

Threshold samples are generated using the reparameterization in Equation (52). The lower‐bound clamping operation applied to these samples in the forward computation is defined in Equation (53).

(56) $$\begin{array}{c} \hat{F}\left(D|\mu ,\rho \right)=\frac{1}{n}\sum_{i=1}^{n}[\log q(\theta^{\left(i\right)}|\mu ,\rho )-\log p\left(\theta^{\left(i\right)}\right)-\log p\left(D|\theta^{\left(i\right)}\right)]\;\;\; \end{array}$$

The prior over thresholds is chosen as a two‐component Gaussian scale mixture,

(57) $$p\left(\theta \right)=\pi N\left(\theta ;\mu_{0},\sigma_{1}^{2}\right)+\left(1-\pi \right)N\left(\theta ;\mu_{0},\sigma_{2}^{2}\right),\;\;\sigma_{1}>\sigma_{2}$$

where $\mu_{0}$, $\sigma_{1}$, $\sigma_{2}$, and π are hyperparameters. The prior mean $\mu_{0}$ is neuron‐specific and is initialized by sampling from a normal distribution with prescribed mean and standard deviation. The scale parameters $\sigma_{1}$ and $\sigma_{2}$, as well as the mixture weight π, are kept constant. None of these prior parameters are updated during training, only the variational parameters μ and ρ are learned.

For a fixed sample of θ, the sample‐wise gradient of the MC objective with respect to θ can be written as

(58) $$\frac{\partial \hat{F}}{\partial \theta }=\frac{\partial }{\partial \theta }\log q\left(\theta |\mu ,\rho \right)-\frac{\partial }{\partial \theta }\log p\left(\theta \right)-\frac{\partial }{\partial \theta }\log p\left(D|\theta \right)$$

The likelihood term factorizes over data points,

(59) $$\log p\left(D|\theta \right)=\sum_{j=1}^{N}\log p(y^{\left(j\right)}|x^{\left(j\right)},\theta )$$

and its gradient with respect to θ is obtained by backpropagating through the SBNN with the sampled thresholds treated as deterministic parameters for each Monte Carlo draw.

In practice, the KL divergence between $q\left(\theta |\mu ,\rho \right)$ and $p\left(\theta \right)$ is evaluated by averaging the difference $\log q\left(\theta \right)-\log p\left(\theta \right)$ over a fixed number of samples per neuron. This KL term is added to the task loss, and gradients with respect to μ and ρ are computed by automatic differentiation through the reparameterization and the truncated sampling procedure. The resulting gradients are then used by a standard stochastic optimizer to update μ and ρ.

#### 4.8.1 Probabilistic Interpretation

We interpret the spiking process as a Bernoulli random variable obtained by marginalizing over the random threshold, and we assume conditional independence of neurons within each layer given their inputs. The stochastic behavior of the SBNN arises at the level of individual neurons. For a neuron j in layer ℓ, receiving input $x_{j}^{\ell }$, the event of emitting a spike $S_{j}^{\ell }=1$ is random. Its probability is determined by the Cumulative Distribution Function (CDF) $F_{j}^{\ell }$ of the threshold distribution $p_{j}^{\ell }\left(\theta \right)$,

(60) $$P\left(S_{j}^{\ell }=1|S^{\ell -1}\right)=F_{j}^{\ell }\left(x_{j}^{\ell }\right)=\int_{-\infty }^{x_{j}^{\ell }}p_{j}^{\ell }\left(\theta \right)d\theta$$

where $S^{\ell -1}$ is the spike vector in the preceding layer. Assuming conditional independence of thresholds within a layer, the transition probability from layer ℓ−1 to layer ℓ factorizes as

(61) $$P\left(S^{\ell }|S^{\ell -1}\right)=\prod_{j}P(S_{j}^{\ell }|S^{\ell -1})$$

with

(62) $$S^{\ell }=\left(S_{1}^{\ell },S_{2}^{\ell },\text{…},S_{n}^{\ell }\right)$$

The full network defines a joint distribution over all latent and output spike states, conditioned on the input $S^{0}$, that factorizes along the network topology. The conditional distribution of the output layer is obtained by marginalizing over the hidden layers $S^{1},\text{…},S^{L-1}$,

(63) $$P\left(S^{L}|S^{0}\right)=\sum_{S^{1},\text{…},S^{L-1}}\prod_{\ell =1}^{L}P(S^{\ell }|S^{\ell -1})$$

Hence, the probability of a particular output spike pattern is the sum of the probabilities over all admissible spike configurations in the hidden layers that can give rise to it.

### 4.9 Device Characterization and Extraction of Switching Parameters

The switching curve in Figure 2C was obtained from a single MTJ. Separately, eight MTJs were characterized to assess the repeatability of their switching responses under fixed measurement conditions. The HIL inference experiment used a single physical MTJ device, with the device assignment and evaluation procedure described in Section 4.11. Figure 2C provides the reference switching characteristic used for input mapping. The eight‐device measurements assess repeatability under the specified measurement conditions.

For the eight‐device repeatability assessment, measurements for each device were divided into 100 consecutive windows, with 100 switching trials per voltage point in each window. The switching center and slope factor were extracted separately for each window. The within‐device statistics reported in Section 2.8 quantify window‐to‐window repeatability and are distinguished from the variation of mean switching parameters across devices.

To characterize device‐level stochastic switching, we measured the antiparallel‐to‐parallel switching probability of the MTJ as a function of the applied pulse voltage (Figure 2C). For each voltage in the range 0.37−0.60V with a step size of 0.01V, 10000 identical pulses were applied. The MTJ state after each pulse was determined from the post‐pulse resistance readout, and the switching probability was computed as the fraction of pulses that induced a successful transition. The resulting probability‐voltage relation was fit using a sigmoid function, yielding a characteristic switching voltage $V_{50}=0.4997\pm 0.014V$ (mean±s.d.), defined as the voltage corresponding to 50% switching probability.

### 4.10 Hardware‐in‐the‐Loop Setup and Input Mapping

The device‐based evaluation was conducted using a HIL setup combining software network computation with physical MTJ switching measurements. Network training was performed entirely in software. During HIL inference, the trained synaptic weights and neuronal threshold parameters remained stored in software. Input encoding, weighted summation, input‐to‐voltage mapping, and output processing were performed in software, whereas stochastic neuronal outputs were generated from the measured switching events of physical MTJ devices. The setup therefore evaluated MTJ‐based stochastic neuronal operation within a network inference procedure, rather than a fully integrated hardware neural network. For a neuron with learned threshold mean μ and algorithmic input $x_{in}$, the modulated input was defined as

(64) $$x_{mod}=\frac{V_{50}\cdot x_{in}}{\mu }$$

where $V_{50}=0.4997V$ is obtained from the sigmoid fit described above. The modulated inputs were subsequently mapped to MTJ drive voltages according to this expression, enabling neuron‐specific threshold alignment while using a common device switching curve.

All electrical measurements were performed using a Keithley 4200A‐SCS semiconductor parameter analyzer equipped with a 4225‐PMU pulse measure unit and a 4225‐RPM remote amplifier/switch. The measured switching curve is used for post‐training input mapping rather than in the training forward model. This mapping aligns the 50% switching point but does not independently control each neuron's effective threshold‐distribution width.

Let P(V) denote the experimentally measured switching‐probability response under the HIL operating conditions, and let $V_{5}0$ denote its switching midpoint. Under Equation (2), the effective neuronal firing probability is $P_{HIL,i}\left(x\right)=P\left(V_{50}x/\mu_{i}\right)$. Therefore, $P_{HIL,i}\left(\mu_{i}\right)=0.5$, but the remaining shape of the response is inherited from the measured device characteristic through input scaling, as is shown in Figures S21– S23.

### 4.11 Hardware‐in‐the‐Loop Inference Procedure and Evaluation

An SBNN with a 784‐20‐10 multilayer perceptron architecture was trained on the full MNIST training set in software. HIL inference was evaluated on a randomly selected, class‐balanced subset of 100 MNIST test images, comprising 10 images per class. The software and HIL evaluations used the same trained parameters and test images. A single physical MTJ was sequentially reused to evaluate the logical neurons in the hidden and output layers.

Each image was encoded into a binary input spike sequence in software. For each hidden‐layer neuronal evaluation, software computed the weighted sum of the input spikes and converted it into a drive voltage using the mapping in Section 4.10. The MTJ was initialized to the antiparallel state before the corresponding drive pulse was applied. Its post‐pulse resistance was then measured to determine the neuronal output: a transition to the parallel state represented a spike, whereas retention of the antiparallel state represented no spike.

The measured hidden‐layer spike outputs were returned to software and combined with the learned output‐layer weights to calculate the output‐layer drive voltages. Output‐layer spike events were obtained using the same MTJ initialization, pulse application, and resistance‐readout procedure. Thus, the measured device outputs participated in the subsequent network computation.

A total sampling budget of 16 steps per image was used. For the eight‐step, two‐Monte‐Carlo‐sample evaluation reported in Figure 4H and I, these steps were organized into two groups of eight. Output aggregation, predicted‐label determination, and the calculation of accuracy and negative log likelihood were performed in software.

Figure 4D,E,G–I compares the software and HIL results. Figure 4A–C,F reports software simulations and do not involve physical MTJ operation.

### 4.12 Modeling and Evaluation of MTJ Variability

#### 4.12.1 Literature‐Based Reconstruction

Static device‐to‐device (D2D) parameter distributions were reconstructed from published switching‐parameter histograms for 250 MTJs (distribution 1; Figure S3 of Ref. [74]) and 144 MTJs (distribution 2; Figure S1 of Ref. [75]). Histogram bin boundaries and counts were digitized, and each bin was populated with equally spaced subinterval midpoints while preserving its count. Because the source histograms report separate center and slope distributions, the reconstructed arrays were independently permuted and paired using seeds 72020 and 71128 for distributions 1 and 2, respectively.

For each reconstructed record, the relative switching‐center offset $\delta_{j}$ and normalized slope factor $r_{j}$ were calculated as

(65) $$\delta_{j}=\frac{V_{50,j}^{\mathrm{src}}}{{\mathrm{median}}_{\ell }\left(V_{50,\ell }^{\mathrm{src}}\right)}-1,\;r_{j}=\frac{|\alpha_{j}|V_{50,j}^{\mathrm{src}}}{{\mathrm{median}}_{\ell }\left(\right|\alpha_{\ell }\left|V_{50,\ell }^{\mathrm{src}}\right)}$$

where $V_{50,j}^{\mathrm{src}}$ is the source switching center and $\alpha_{j}$ is the sigmoid coefficient in inverse voltage units. The geometric standard deviation was calculated as GSD(r)=exp[SD(lnr)], using population standard deviation with denominator N. For each network realization, 1010 parameter pairs $\left(\delta_{j},r_{j}\right)$ were sampled with replacement, assigned to the hidden and output neurons, and held fixed throughout inference without further clipping or recentering.

#### 4.12.2 Static and Temporal Variability

Static switching parameters were defined as $V_{50,i}^{D}=V_{0}\left(1+\delta_{i}\right)$ and $k_{i}^{D}=k_{0}r_{i}$, with nominal parameters $V_{0}=0.4997\;V$ and $k_{0}=64.7$. For synthetic D2D sweeps, center offsets and slope factors were sampled independently:

(66) $$\begin{array}{cc} \delta_{i}∼\; & U(-a,a)\\ r_{i}=\; & \mathrm{clip}\left[\exp\left(-\frac{s_{D}^{2}}{2}+s_{D}Z_{i}\right),0.25,2.25\right]\\ Z_{i}∼\; & N(0,1) \end{array}$$

where $s_{D}=\ln g_{D}$ and clip(x,l,u)=min[u,max(l,x)]. The generating lognormal distribution has arithmetic mean 1 and GSD $g_{D}$ before clipping, and $g_{D}$ is therefore termed the nominal D2D slope GSD. The joint D2D sweeps in Figure 5B,C used offset half‐ranges a∈{0.05,0.10,0.15,0.20} and nominal GSDs $g_{D}\in \left\{1.1,1.25,1.5,2.0\right\}$.

Cycle‐to‐cycle (C2C) variability was introduced through temporal perturbations of the static parameters:

(67) $$\begin{array}{c} V_{50,i,t}=\max\left[V_{50,i}^{D}\left(1+\varepsilon_{i,t}\right),v_{\min}\right],\;k_{i,t}=k_{i}^{D}\exp\left(-\frac{s_{C}^{2}}{2}+s_{C}Z_{i,t}\right)\;\;\; \end{array}$$

where $\varepsilon_{i,t}∼N\left(0,s_{V}^{2}\right)$, $Z_{i,t}∼N\left(0,1\right)$, $s_{C}=\ln g_{C}$, and $v_{\min}$ is a numerical positivity bound. Here, $s_{V}$ specifies the relative center standard deviation before bounding, and $g_{C}$ specifies the GSD of the unclipped C2C slope multiplier. Perturbations were sampled independently for each neuron, input sample, and time step. The C2C stress tests in Figure 5D used $s_{V}\in \left\{0,0.05\right\}$ and $g_{C}\in \left\{4.0,5.0,7.5\right\}$ on a D2D background with a=0.20 and $g_{D}=2.0$. The D2D‐only reference used $s_{V}=0$ and $g_{C}=1$.

#### 4.12.3 Switching Model and Calibration

For an applied voltage V, the instantaneous switching probability was

(68) $$P_{i,t}\left(V\right)={\left[1+\exp\left(-k_{i,t}\left(\frac{V}{V_{50,i,t}}-1\right)\right)\right]}^{-1}$$

from which a Bernoulli spike was sampled. Defining $z_{i}=x_{i}/\mu_{i}$, where $x_{i}$ is the neuronal signal and $\mu_{i}$ is its learned threshold mean, the three voltage mappings were

(69) $$\begin{array}{cc} V_{i}^{\mathrm{shared}} & =V_{0}z_{i},\\ V_{i}^{\mathrm{center}} & =V_{50,i}^{D}z_{i},\\ V_{i}^{\mathrm{full}} & =V_{50,i}^{D}\left[1+\frac{k_{0}}{k_{i}^{D}}\left(z_{i}-1\right)\right] \end{array}$$

These mappings implement shared, center‐only, and full static calibration, respectively. Calibration used the assigned static parameters and remained fixed during inference, with no drive‐voltage bounds imposed. The learned neuronal parameters were identical across the three calibration strategies. Only the compensation for static device variability differed. Device‐specific calibration used the assigned static parameters and therefore represents an idealized correction benchmark rather than a measured calibration circuit. Here, full static calibration refers to compensation of static device‐specific center and slope deviations relative to the nominal sigmoid response; it does not denote independent realization of each learned Gaussian threshold variance or exact reproduction of the algorithmic threshold distribution.

#### 4.12.4 Evaluation and Statistics

All conditions were evaluated using the same pretrained 784–1000–10 network with 8‐bit weights on the full 10000‐image MNIST test set at T=16, without retraining. Thirty paired realizations used device‐population seeds 1000–1029 and inference seeds 2000–2029. Means and 95% confidence intervals were calculated across realizations as $\bar{Y}\pm t_{0.975,29}s_{Y}/\sqrt{30}$, where $s_{Y}$ is the sample standard deviation. For paired comparisons, Y denotes the within‐realization accuracy difference.

Accuracy changes were expressed in percentage points as $\Delta A=A_{\mathrm{reference}}-A_{\mathrm{variation}}$. Figure 5B–C used the no‐variation reference, whereas C2C accuracy changes used the corresponding mapping's D2D‐only reference.

### 4.13 Computational Benchmarking and RE Method Error

RE and SG were benchmarked on an NVIDIA A800 GPU with a batch size of 64. Timing excluded data loading and warm‐up. The benchmarks used the same optimizer, precision, and epoch protocol. Wall‐clock time was measured over 20 epochs after 10 warm‐up epochs. The reported timing excludes validation and checkpoint writing. Peak memory was measured using allocated and reported in MiB. For approximation‐error evaluation, RE firing probabilities and explicit spike averages were compared for output layers using final check points. RMSE was computed over layers at each T. This evaluation separates agreement of the firing‐rate approximation from the classification accuracy obtained after separately training the two models.

### 4.14 Energy Estimation

For the prospective neuron circuit in Figure 2H, a first‐order estimate gives 1.00−8.04nJ per neuronal spike event under the assumptions detailed in Note S3. This estimate includes the MTJ, the fixed modulation resistor, and a nominal comparator contribution, but excludes reset, additional sensing circuitry, drivers, control, and network computation. The estimate uses the measured MTJ characteristics under the 1 μs pulses employed in the HIL experiment, whose operating conditions were not optimized for energy efficiency. Switching has also been observed with 20ns pulses in devices from the same fabrication batch, indicating scope for reducing the sampling energy through shorter‐pulse operation. For context, MTJ‐based neuron studies have reported approximately 4pJ per signed‐spike operation [79], 4−16pJ per spike at the device level [80], and 0.190pJ per device switching event [81], under different operating conditions and energy‐accounting boundaries.

The architectural advantage of the proposed integrated implementation is that intrinsic MTJ stochasticity performs sampling directly during neuronal spike generation. This fusion removes the need for a separate random‐number generator and an explicit random‐number‐generation‐and‐transfer stage before firing. It therefore offers a route to reducing additional peripheral circuitry, intermediate random‐sample readout and buffering, and access and synchronization delays associated with a dedicated sampling module.

## Author Contributions

Conceptualization: H. Zheng and K. Yang. Methodology: H. Zheng, J. Liu and K. Yang Formal analysis: H. Zheng and J. Liu. Writing: H. Zheng, J. Liu and K. Yang.

## Conflicts of Interest

The authors declare no conflicts of interest.

## Supporting information


**Supporting File**: advs78508‐sup‐0001‐SuppMat.pdf.

## Data Availability

All data needed to evaluate the conclusions in the paper are present in the paper or the Supporting Information.

## References

[1] Y. LeCun , Y. Bengio , and G. Hinton , “Deep Learning,” Nature 521, no. 7553 (2015): 436–444. 10.1038/nature14539 26017442

[2] A. Stanojevic , S. Woźniak , G. Bellec , G. Cherubini , A. Pantazi , and W. Gerstner , “High‐Performance Deep Spiking Neural Networks With 0.3 Spikes per Neuron,” Nature Communications 15, no. 1 (2024): 6793. 10.1038/s41467-024-51110-5 PMC11315905 39122775

[3] W. Fang , Y. Chen , J. Ding , et al., “Spikingjelly: An Open‐Source Machine Learning Infrastructure Platform for Spike‐Based Intelligence,” Science Advances 9, no. 40 (2023): eadi1480. 10.1126/sciadv.adi1480 PMC10558124 37801497

[4] D. Gehrig and D. Scaramuzza , “Low‐Latency Automotive Vision With Event Cameras,” Nature 629, no. 8014 (2024): 1034–1040. 10.1038/s41586-024-07409-w PMC11136662 38811712

[5] L. Chen , S. Karilanova , S. Chaki , et al., “Spike Timing–Based Coding in Neuromimetic Tactile System Enables Dynamic Object Classification,” Science 384, no. 6696 (2024): 660–665. 10.1126/science.adf3708 38723082

[6] W. Wang , Y. Jiang , D. Zhong , et al., “Neuromorphic Sensorimotor Loop Embodied by Monolithically Integrated, Low‐Voltage, Soft e‐Skin,” Science 380, no. 6646 (2023): 735–742. 10.1126/science.ade0086 37200416

[7] R. Waser and M. Aono , “Nanoionics‐Based Resistive Switching Memories,” Nature Materials 6, no. 11 (2007): 833–840. 10.1038/nmat2023 17972938

[8] H.‐S. P. Wong , H.‐Y. Lee , S. Yu , et al., “Metal–Oxide RRAM,” Proceedings of the IEEE 100, no. 6 (2012): 1951–1970.

[9] J. Feldmann , N. Youngblood , C. D. Wright , H. Bhaskaran , and W. H. Pernice , “All‐Optical Spiking Neurosynaptic Networks With Self‐Learning Capabilities,” Nature 569, no. 7755 (2019): 208–214. 10.1038/s41586-019-1157-8 PMC6522354 31068721

[10] K. Y. Camsari , B. M. Sutton , and S. Datta , “p‐Bits for Probabilistic Spin Logic,” Applied Physics Reviews 6, no. 1 (2019): 011305, 10.1063/1.5055860.

[11] K. Roy , C. Wang , S. Roy , A. Raghunathan , K. Yang , and A. Sengupta , “Spintronic Neural Systems,” Nature Reviews Electrical Engineering 1, no. 11 (2024): 714–729.

[12] Maryada , S. Soldado‐Magraner , M. Sorbaro , R. Laje , D. V. Buonomano , and G. Indiveri , “Stable Recurrent Dynamics in Heterogeneous Neuromorphic Computing Systems Using Excitatory and Inhibitory Plasticity,” Nature Communications 16, no. 1 (2025): 5522. 10.1038/s41467-025-60697-2 PMC12214931 40595529

[13] E. Neftci and G. Indiveri , “A Device Mismatch Compensation Method for VLSI Neural Networks,” in 2010 Biomedical Circuits and Systems Conference (BioCAS) (IEEE, 2010), 262–265.

[14] O. Yousuf , B. D. Hoskins , K. Ramu , et al., “Layer Ensemble Averaging for Fault Tolerance in Memristive Neural Networks,” Nature Communications 16, no. 1 (2025): 1250. 10.1038/s41467-025-56319-6 PMC11787353 39893160

[15] H. Aziza , et al., “Analysis of Conductance Variability in RRAM for Accurate Neuromorphic Computing,” in 2024 IEEE 25th Latin American Test Symposium (LATS) (2024).

[16] T. Dalgaty , F. Moro , Y. Demirağ , et al., “Mosaic: In‐Memory Computing and Routing for Small‐World Spike‐Based Neuromorphic Systems,” Nature Communications 15, no. 1 (2024): 142. 10.1038/s41467-023-44365-x PMC10761708 38167293

[17] D. Marković , A. Mizrahi , D. Querlioz , and J. Grollier , “Physics for Neuromorphic Computing,” Nature Reviews Physics 2, no. 9 (2020): 499–510.

[18] D.‐H. Lim , S. Wu , R. Zhao , J.‐H. Lee , H. Jeong , and L. Shi , “Spontaneous Sparse Learning for PCM‐Based Memristor Neural Networks,” Nature Communications 12, no. 1 (2021): 319. 10.1038/s41467-020-20519-z PMC7803975 33436611

[19] C. Findling and V. Wyart , “Computation Noise Promotes Zero‐Shot Adaptation to Uncertainty During Decision‐Making in Artificial Neural Networks,” Science Advances 10, no. 44 (2024): eadl3931. 10.1126/sciadv.adl3931 PMC11524185 39475619

[20] L. Hertäg , K. A. Wilmes , and C. Clopath , “Uncertainty Estimation With Prediction‐Error Circuits,” Nature Communications 16, no. 1 (2025): 3036. 10.1038/s41467-025-58311-6 PMC11953419 40155399

[21] C. Findling and V. Wyart , “Computation Noise in Human Learning and Decision‐Making: Origin, Impact, Function,” Current Opinion in Behavioral Sciences 38 (2021): 124–132.

[22] D. R. Bach and R. J. Dolan , “Knowing How Much You Don't Know: A Neural Organization of Uncertainty Estimates,” Nature Reviews Neuroscience 13, no. 8 (2012): 572–586. 10.1038/nrn3289 22781958

[23] W. J. Ma and M. Jazayeri , “Neural Coding of Uncertainty and Probability,” Annual Review of Neuroscience 37, no. 1 (2014): 205–220. 10.1146/annurev-neuro-071013-014017 25032495

[24] D. Festa , A. Aschner , A. Davila , A. Kohn , and R. Coen‐Cagli , “Neuronal Variability Reflects Probabilistic Inference Tuned to Natural Image Statistics,” Nature Communications 12, no. 1 (2021): 3635. 10.1038/s41467-021-23838-x PMC8206154 34131142

[25] A. Farzmahdi , A. Kohn , and R. Coen‐Cagli , “Relating Natural Image Statistics to Patterns of Response Covariability in Macaque Primary Visual Cortex,” Nature Communications 16, no. 1 (2025): 6757. 10.1038/s41467-025-62086-1 PMC12284261 40695825

[26] Y. Qi and P. Gong , “Fractional Neural Sampling as a Theory of Spatiotemporal Probabilistic Computations in Neural Circuits,” Nature Communications 13, no. 1 (2022): 4572. 10.1038/s41467-022-32279-z PMC9356069 35931698

[27] R. Echeveste , L. Aitchison , G. Hennequin , and M. Lengyel , “Cortical‐like Dynamics in Recurrent Circuits Optimized for Sampling‐Based Probabilistic Inference,” Nature Neuroscience 23, no. 9 (2020): 1138–1149. 10.1038/s41593-020-0671-1 PMC7610392 32778794

[28] J. M. Rowland , T. L. van der Plas , M. Loidolt , et al., “Propagation of Activity Through the Cortical Hierarchy and Perception are Determined by Neural Variability,” Nature Neuroscience 26, no. 9 (2023): 1584–1594. 10.1038/s41593-023-01413-5 PMC10471496 37640911

[29] W.‐H. Zhang , S. Wu , K. Josić , and B. Doiron , “Sampling‐Based Bayesian Inference in Recurrent Circuits of Stochastic Spiking Neurons,” Nature Communications 14, no. 1 (2023): 7074. 10.1038/s41467-023-41743-3 PMC10625605 37925497

[30] A. Pouget , J. Drugowitsch , and A. Kepecs , “Confidence and Certainty: Distinct Probabilistic Quantities for Different Goals,” Nature Neuroscience 19, no. 3 (2016): 366–374. 10.1038/nn.4240 PMC5378479 26906503

[31] E. Y. Walker , S. Pohl , R. N. Denison , et al., “Studying the Neural Representations of Uncertainty,” Nature Neuroscience 26, no. 11 (2023): 1857–1867. 10.1038/s41593-023-01444-y 37814025

[32] M. B. Wang , N. Lynch , and M. M. Halassa , “The Neural Basis for Uncertainty Processing in Hierarchical Decision Making,” Nature Communications 16, no. 1 (2025): 9096. 10.1038/s41467-025-63994-y PMC12531367 41102143

[33] Z. M. Boundy‐Singer , C. M. Ziemba , and R. L. Goris , “Confidence Reflects a Noisy Decision Reliability Estimate,” Nature Human Behaviour 7, no. 1 (2023): 142–154. 10.1038/s41562-022-01464-x 36344656

[34] W. Maass , T. Natschläger , and H. Markram , “Real‐Time Computing Without Stable States: A New Framework for Neural Computation Based on Perturbations,” Neural Computation 14, no. 11 (2002): 2531–2560. 10.1162/089976602760407955 12433288

[35] W. Maass , “Noise as a Resource for Computation and Learning in Networks of Spiking Neurons,” Proceedings of the IEEE 102, no. 5 (2014): 860–880.

[36] D. Querlioz and E. Vianello , “Bayesian Electronics for Trustworthy Artificial Intelligence,” Nature Reviews Electrical Engineering 2, no. 12 (2025): 846–855.

[37] S. Misra , L. C. Bland , S. G. Cardwell , et al., “Probabilistic Neural Computing With Stochastic Devices,” Advanced Materials 35, no. 37 (2023): 2204569. 10.1002/adma.202204569 36395387

[38] F. Brückerhoff‐Plückelmann , H. Borras , B. Klein , et al., “Probabilistic Photonic Computing With Chaotic Light,” Nature Communications 15, no. 1 (2024): 10445. 10.1038/s41467-024-54931-6 PMC11609296 39617761

[39] N. S. Singh , K. Kobayashi , Q. Cao , et al., “CMOS Plus Stochastic Nanomagnets Enabling Heterogeneous Computers for Probabilistic Inference and Learning,” Nature Communications 15, no. 1 (2024): 2685. 10.1038/s41467-024-46645-6 PMC10973401 38538599

[40] Y. Zheng , H. Ravichandran , T. F. Schranghamer , N. Trainor , J. M. Redwing , and S. Das , “Hardware Implementation of Bayesian Network Based on Two‐Dimensional Memtransistors,” Nature Communications 13, no. 1 (2022): 5578. 10.1038/s41467-022-33053-x PMC9508127 36151079

[41] Y. Lin , Q. Zhang , B. Gao , et al., “Uncertainty Quantification via a Memristor Bayesian Deep Neural Network for Risk‐Sensitive Reinforcement Learning,” Nature Machine Intelligence 5, no. 7 (2023): 714–723.

[42] M. Song , R.‐H. Koo , J. Kim , et al., “Ferroelectric NAND for Efficient Hardware Bayesian Neural Networks,” Nature Communications 16, no. 1 (2025): 6879. 10.1038/s41467-025-61980-y PMC12297355 40715069

[43] D. Bonnet , T. Hirtzlin , A. Majumdar , et al., “Bringing Uncertainty Quantification to the Extreme‐Edge With Memristor‐Based Bayesian Neural Networks,” Nature Communications 14, no. 1 (2023): 7530. 10.1038/s41467-023-43317-9 PMC10661910 37985669

[44] T. Wang , B. Dai , S. Xu , et al., “Spintronic Bayesian Hardware Driven by Stochastic Magnetic Domain Wall Dynamics,” Advanced Science 13, no. 27 (2026): e20717. 10.1002/advs.202520717 PMC13170188 41841603

[45] H. Ravichandran , T. Knobloch , S. Subbulakshmi Radhakrishnan , et al., “A Stochastic Encoder Using Point Defects in Two‐Dimensional Materials,” Nature Communications 15, no. 1 (2024): 10562. 10.1038/s41467-024-54283-1 PMC11618794 39632808

[46] R. Midya , A. S. Pawar , D. P. Pattnaik , et al., “Artificial Transneurons Emulate Neuronal Activity in Different Areas of Brain Cortex,” Nature Communications 16, no. 1 (2025): 7289. 10.1038/s41467-025-62151-9 PMC12332047 40774953

[47] W. Zhu , Y. Zhu , T. Zhou , et al., “Large and Tunable Magnetoresistance in Van der Waals Ferromagnet/Semiconductor Junctions,” Nature Communications 14, no. 1 (2023): 5371. 10.1038/s41467-023-41077-0 PMC10477182 37666843

[48] E. O. Neftci , H. Mostafa , and F. Zenke , “Surrogate Gradient Learning in Spiking Neural Networks,” IEEE Signal Processing Magazine 36, no. 6 (2019): 51–63.

[49] S. S. Chowdhury , N. Rathi , and K. Roy , “One Timestep is All You Need: Training Spiking Neural Networks With Ultra Low Latency,” arXiv preprint arXiv:2110.05929 (2021).

[50] H. Jiang , S. Anumasa , G. De Masi , H. Xiong , and B. Gu , “A Unified Optimization Framework of ANN‐SNN Conversion: Towards Optimal Mapping From Activation Values to Firing Rates,” in International Conference on Machine Learning (PMLR, 2023), 14945–14974.

[51] Q. Meng , M. Xiao , S. Yan , Y. Wang , Z. Lin , and Z.‐Q. Luo , “Towards Memory‐and Time‐Efficient Backpropagation for Training Spiking Neural Networks,” in Proceedings of the IEEE/CVF international conference on computer vision (IEEE, 2023), 6166–6176.

[52] G. Wang , Y. Sun , S. Cheng , and S. Song , “Evolving Connectivity for Recurrent Spiking Neural Networks,” Advances in Neural Information Processing Systems 36 (2023): 2991–3007.

[53] T. Bu , M. Li , and Z. Yu , “Inference‐Scale Complexity in ANN‐SNN Conversion for High‐Performance and Low‐Power Applications,” in Proceedings of the Computer Vision and Pattern Recognition Conference (2025), 24387–24397.

[54] C. Blundell , J. Cornebise , K. Kavukcuoglu , and D. Wierstra , “Weight Uncertainty in Neural Network,” in International conference on machine learning (2015), 1613–1622.

[55] M. Ezzadeen , A. Majumdar , O. Valorge , et al., “Implementation of Binarized Neural Networks Immune to Device Variation and Voltage Drop Employing Resistive Random Access Memory Bridges and Capacitive Neurons,” Communications Engineering 3, no. 1 (2024): 80.

[56] L. Taylor , A. King , and N. Harper , “Robust and Accelerated Single‐Spike Spiking Neural Network Training With Applicability to Challenging Temporal Tasks,” preprint, arXiv, May 30, 2022, 10.48550/arXiv.2205.15286.

[57] A. Laborieux , M. Ernoult , T. Hirtzlin , and D. Querlioz , “Synaptic Metaplasticity in Binarized Neural Networks,” Nature communications 12, no. 1 (2021): 2549. 10.1038/s41467-021-22768-y PMC8100137 33953183

[58] E. Vargas , C. V. Correa , C. Hinojosa , and H. Arguello , “Biper: Binary Neural Networks Using a Periodic Function,” in Proceedings of the IEEE/CVF conference on computer vision and pattern recognition (IEEE, 2024), 5684–5693.

[59] T. Bu , W. Fang , J. Ding , P. Dai , Z. Yu , and T. Huang , “Optimal ANN‐SNN Conversion for High‐Accuracy and Ultra‐Low‐Latency Spiking Neural Networks,” preprint, arXiv, March 8, 2023, 10.48550/arXiv.2303.04347.

[60] Y. Guo , Y. Chen , L. Zhang , et al., “IM‐Loss: Information Maximization Loss for Spiking Neural Networks,” Advances in Neural Information Processing Systems 35 (2022): 156–166.

[61] W. Fang , Z. Yu , Y. Chen , T. Huang , T. Masquelier , and Y. Tian , “Deep Residual Learning in Spiking Neural Networks,” Advances in Neural Information Processing Systems 34 (2021): 21056–21069.

[62] W. Fang , Z. Yu , Y. Chen , T. Masquelier , T. Huang , and Y. Tian , “Incorporating Learnable Membrane Time Constant to Enhance Learning of Spiking Neural Networks,” in Proceedings of the IEEE/CVF international conference on computer vision (IEEE, 2021), 2661–2671.

[63] Z. Zhou , Y. Zhu , C. He , et al., “Spikformer: When Spiking Neural Network Meets Transformer,” arXiv preprint arXiv:2209.15425 (2022).

[64] C. Zhou , H. Zhang , Z. Zhou , et al., “Qkformer: Hierarchical Spiking Transformer Using QK Attention,” Advances in Neural Information Processing Systems 37 (2024): 13074–13098.

[65] Y. Fang , Z. Wang , L. Zhang , J. Cao , H. Chen , and R. Xu , “Spiking Wavelet Transformer,” in European Conference on Computer Vision (Springer, 2024), 19–37.

[66] Z. Zheng , Y. Huang , Y. Yu , et al., “SpiLiFormer: Enhancing Spiking Transformers With Lateral Inhibition,” in Proceedings of the IEEE/CVF International Conference on Computer Vision (IEEE, 2025), 24539–24548.

[67] R. Li , M. Klasson , A. Solin , and M. Trapp , “Streamlining Prediction in Bayesian Deep Learning,” arXiv preprint arXiv:2411.18425 (2024).

[68] Y. Liu and X. Wang , “Implicit Generative Prior for Bayesian Neural Networks,” The Annals of Applied Statistics 18, no. 4 (2024): 2840–2862.

[69] M. Tomczak , S. Swaroop , A. Foong , and R. Turner , “Collapsed Variational Bounds for Bayesian Neural Networks,” Advances in Neural Information Processing Systems 34 (2021): 25412–25426.

[70] F. Wenzel , J. Snoek , D. Tran , and R. Jenatton , “Hyperparameter Ensembles for Robustness and Uncertainty Quantification,” Advances in Neural Information Processing Systems 33 (2020): 6514–6527.

[71] I. Westby , X. Yang , T. Liu , and H. Xu , “FPGA Acceleration on a Multi‐Layer Perceptron Neural Network for Digit Recognition,” The Journal of Supercomputing 77, no. 12 (2021): 14356–14373.

[72] Q. Dariol , S. Le Nours , S. Pillement , R. Stemmer , D. Helms , and K. Grüttner , “A Hybrid Performance Prediction Approach for Fully‐Connected Artificial Neural Networks on Multi‐core Platforms,” in International Conference on Embedded Computer Systems (Springer, 2022), 250–263.

[73] S.‐Y. Ma , T. Wang , J. Laydevant , L. G. Wright , and P. L. McMahon , “Quantum‐Limited Stochastic Optical Neural Networks Operating at a Few Quanta Per Activation,” Nature Communications 16, no. 1 (2025): 359. 10.1038/s41467-024-55220-y PMC11698857 39753530

[74] S. Yang , A. Grimaldi , Y. Bao , et al., “250 Magnetic Tunnel Junctions‐Based Probabilistic Ising Machine,” Nature Communications 17, no. 1 (Apr 2026): 5368, 10.1038/s41467-026-72020-8. PMC13276125 41997933

[75] S. Yang , Y. Bao , E. Humianto , A. Prabhakar , and H. Yang , “A Parallel Magnetic Tunnel Junction‐Based Probabilistic Ising Processor for Efficient Quadratic Optimization,” Nature Communications 17, no. 1 (Mar 2026): 4616, 10.1038/s41467-026-71128-1. PMC13199408 41912511

[76] Y. Lin , B. Gao , J. Tang , Q. Zhang , H. Qian , and H. Wu , “Deep Bayesian Active Learning Using In‐Memory Computing Hardware,” Nature Computational Science 5, no. 1 (2025): 27–36. 10.1038/s43588-024-00744-y PMC11774754 39715830

[77] X. Wang , R. Wang , S. Sun , et al., “Amorphous Phase‐Change Memory Alloy With no Resistance Drift,” Nature Materials 25, no. 3 (2026): 456–462. 10.1038/s41563-025-02361-0 PMC12960214 41034616

[78] Q. Shao , Z. Wang , Y. Zhou , S. Fukami , D. Querlioz , and L. O. Chua , “Spintronic Memristors for Computing,” npj Spintronics 3, no. 1 (2025): 16.

[79] H. Zheng , J. Liu , and K. Yang , “Signed Spiking Neuron Enabled by an Orthogonal‐Easy‐Axis Magnetic Tunnel Junction,” IEEE Electron Device Letters 47, no. 9 (2026): 1954–1957, 10.1109/LED.2026.3708998.

[80] L. Farcis , B. M. Teixeira , P. Talatchian , et al., “Spiking Dynamics in Dual Free Layer Perpendicular Magnetic Tunnel Junctions,” Nano Letters 23, no. 17 (2023): 7869–7875. 10.1021/acs.nanolett.3c01597 37589447

[81] Z. Chen , D. Zhu , A. Du , et al., “Nanoscale Exchange‐Bias Magnetic Tunnel Junctions Enabled Memristive Synapse and Leaky‐Integrate‐Fire Neuron for Neuromorphic Computing,” Nature Communications 17, no. 1 (2026): 4362. 10.1038/s41467-026-70802-8 PMC13172308 41876517

[82] R. Vinuesa , H. Azizpour , I. Leite , et al., “The Role of Artificial Intelligence in Achieving the Sustainable Development Goals,” Nature Communications 11, no. 1 (2020): 233. 10.1038/s41467-019-14108-y PMC6957485 31932590

[83] A. Ross , N. Leroux , A. De Riz , et al., “Multilayer Spintronic Neural Networks With Radiofrequency Connections,” Nature Nanotechnology 18, no. 11 (2023): 1273–1280. 10.1038/s41565-023-01452-w 37500772

[84] J. Ding , B. Dong , F. Heide , et al., “Biologically Inspired Dynamic Thresholds for Spiking Neural Networks,” Advances in Neural Information Processing Systems 35 (2022): 6090–6103.

