# Supplementary material for: Device Stochasticity Directly Implements Bayesian Sampling in Spiking Neurons
Source: Adv Sci (Weinh). 2026 Nov 3:e78508. Online ahead of print. doi: 10.1002/advs.78508 (PMC13731086; doi:10.1002/advs.78508)
Supplement: Supplementary file 1 [file ADVS-9999-e78508-s001.pdf]

Supporting Information for

Device Stochasticity Directly Implements Bayesian Sampling in Spiking Neurons

Huannan Zheng *et al.*

*Corresponding author. Email: kezhouyang@hkust-gz.edu.cn

This PDF file includes:

Supplementary Text S1 to S3
Fig. S1 to S23
Table S1 to S8

Supplementary Text

Note 1: Experiment Setting of Image Recognition Tasks

We evaluate our spiking Bayesian neural network (SBNN) on MNIST, Fashion-MNIST, and CIFAR-10. Inputs are normalized to ($[0,1]$) and arranged in the format required by each network architecture.

We also evaluate SBNNs on event-based datasets, including CIFAR10-DVS, N-Caltech101, and DVS128 Gesture. Event streams are converted into fixed-length frame sequences by dividing each sample into (T) temporal bins according to event count. Each frame contains two polarity channels and retains its spatial structure. For N-Caltech101, the integrated event frames are resized to (128×128) pixels.

Note 2: Encoding of the Inputs

For all inputs, we used a Poisson encoder to convert normalized intensities $x \in [0,1]$into spike trains of length T. For each element (pixel/feature) and each time step t, we generated a spike by drawing $u_t \sim \mathrm{Uniform}(0,1)$and setting

$$s_t = \mathbb{I}[u_t < x],$$

which is equivalent to $s_t \sim \mathrm{Bernoulli}(x)$. This process is performed independently across time steps and input dimensions, yielding outputs of shape $(B, \dots, T)$.

Note 3: Energy Estimation of the Stochastic Neuron Sampling Circuit

To estimate the energy consumption of the prospective neuron circuit illustrated in Figure 2H, we consider the magnetic tunnel junction (MTJ), the series input-modulation resistor, and one complementary metal–oxide–semiconductor (CMOS) comparator. Let (x_{in}) denote the model input and (μ) the learned threshold mean. We explicitly assume a physical input-voltage scale of ($V_{\mathrm{in}} = a x_{\mathrm{in}}$), with ($a = 1$ V) per model-input unit. At ($x_{\mathrm{in}} = \mu$), the voltage across the MTJ is

calibrated to the switching-probability midpoint, ($V_{50} = 0.4997\,\mathrm{V}$). Approximating the MTJ resistance by its antiparallel-state value, ($R_{\mathrm{AP}} = 260\ \Omega$), gives

$$R_{\mathrm{mod}} = R_{\mathrm{AP}}\left(\frac{a\mu}{V_{50}} - 1\right), \qquad R_{\mathrm{tot}} = R_{\mathrm{AP}} + R_{\mathrm{mod}} = \frac{a\mu}{V_{50}} R_{\mathrm{AP}}.$$

The modulation resistance is calibrated once and remains fixed for each neuron. For $\mu = 0.9\text{-}2.9$, the calculated (R_{mod}) and (R_{tot}) are approximately $208.3\text{-}1248.9\Omega$ and $468.3\text{-}1508.9\Omega$, respectively. During a sampling pulse, the MTJ working voltage (V_{MTJ}) spans $0.38\text{-}0.60V$, whereas the source voltage is ($V_{\mathrm{in}} = (a\mu/V_{50})V_{\mathrm{MTJ}}$), spanning approximately $0.684\text{-}3.482V$ over the specified parameter envelope.

Using a pulse duration of ($t_{\mathrm{w}} = 1\ \mu s$), the sampling-core energy is estimated as

$$E_{\mathrm{core}} \approx E_{\mathrm{MTJ}} + E_{\mathrm{mod}} + E_{\mathrm{comp}} \ = \frac{V_{\mathrm{MTJ}}^2}{R_{\mathrm{AP}}} t_{\mathrm{w}} + \left(\frac{V_{\mathrm{MTJ}}}{R_{\mathrm{AP}}}\right)^2 R_{\mathrm{mod}} t_{\mathrm{w}} + E_{\mathrm{comp}} \ = \frac{a\mu}{V_{50}} \frac{V_{\mathrm{MTJ}}^2}{R_{\mathrm{AP}}} t_{\mathrm{w}} + E_{\mathrm{comp}}.$$

A nominal comparator contribution of ($E_{\mathrm{comp}} = 30\ \mathrm{fJ}$) per decision is adopted from the literature [1], assuming one comparison per time step. The resulting estimate is $1.00\text{-}8.04nJ$ per neuronal spike event. This range represents the envelope of the specified parameter ranges, rather than a dataset-averaged or experimentally measured energy.

The estimate assumes a constant AP resistance throughout the pulse and does not track switching resistance changes. It excludes reset and initialization, additional sensing and reference-voltage circuitry beyond the nominal comparator contribution, pulse generation and drivers, input-voltage generation and level shifting, resistor programming and calibration, timing and control, memory access, and synaptic/network computation. Accordingly, it is a conditional neuron working estimate.

Fig. S1.

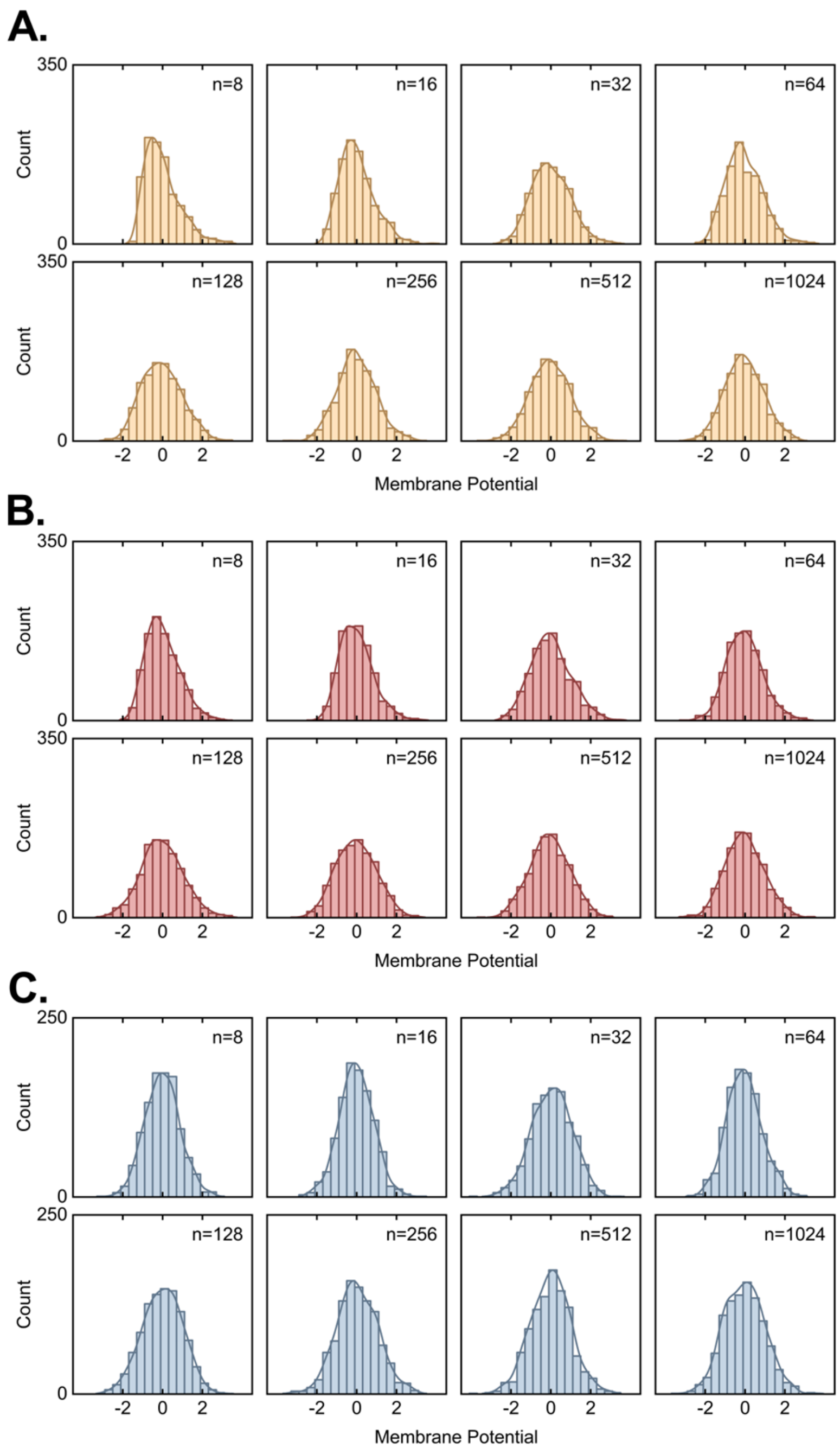

Figure S1: The distribution of membrane potential, where the uncertainty of the weights belongs to the exponential family distribution. (A) Exponential. (B) Gamma. (C) Gaussian.

Fig. S2.

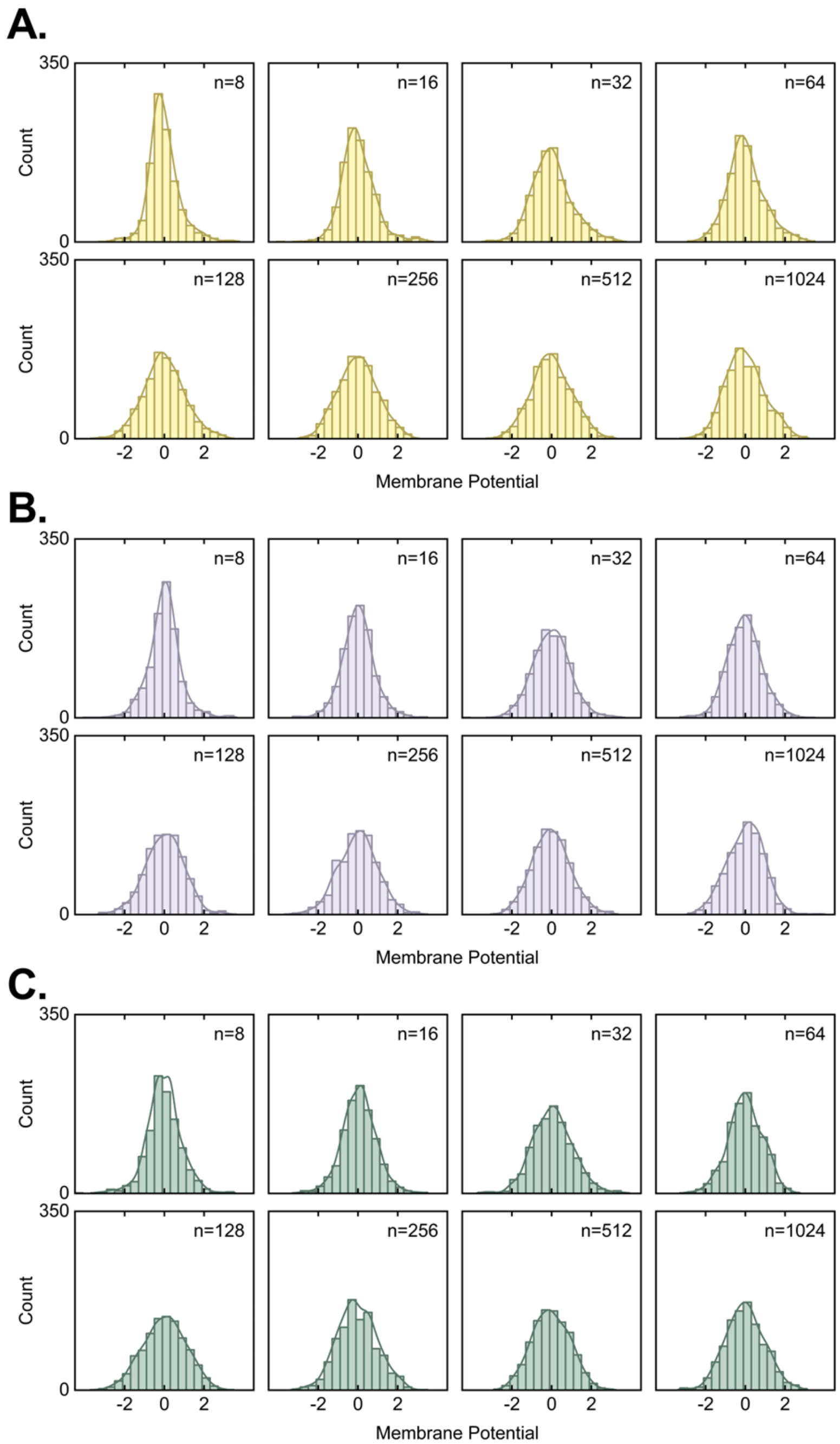

Figure S2: The distribution of membrane potential, where the uncertainty of the weights belongs to the non-exponential family distribution. (A) Lévy Stable. (B) Student-T. (C) Symmetric Pareto.

Fig. S3.

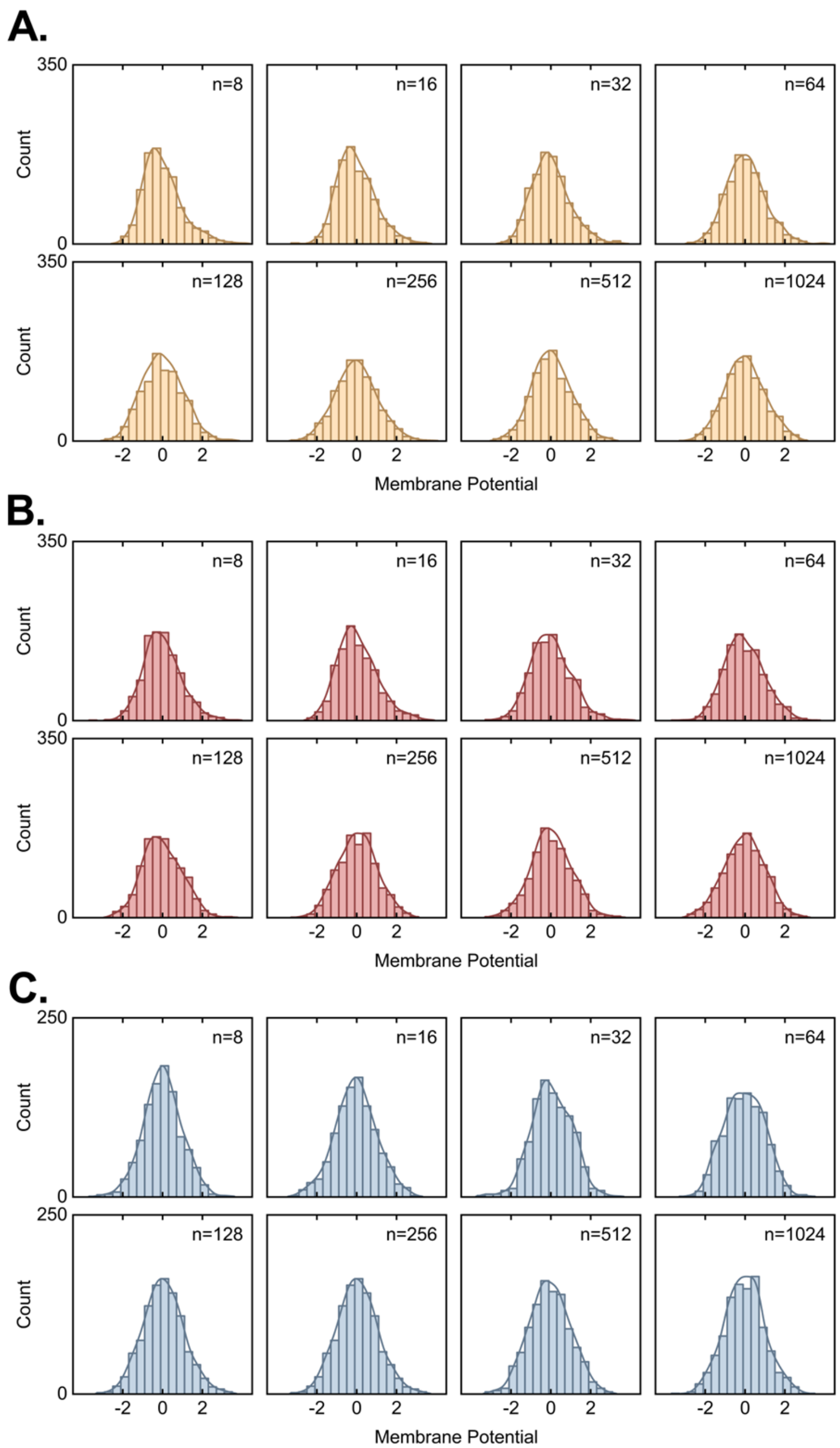

Figure S3: The distribution of membrane potential, where the uncertainty of the weights belongs to the exponential family distribution. In addition, the inputs are random in different samples. (A) Exponential. (B) Gamma. (C) Gaussian.

Fig. S4.

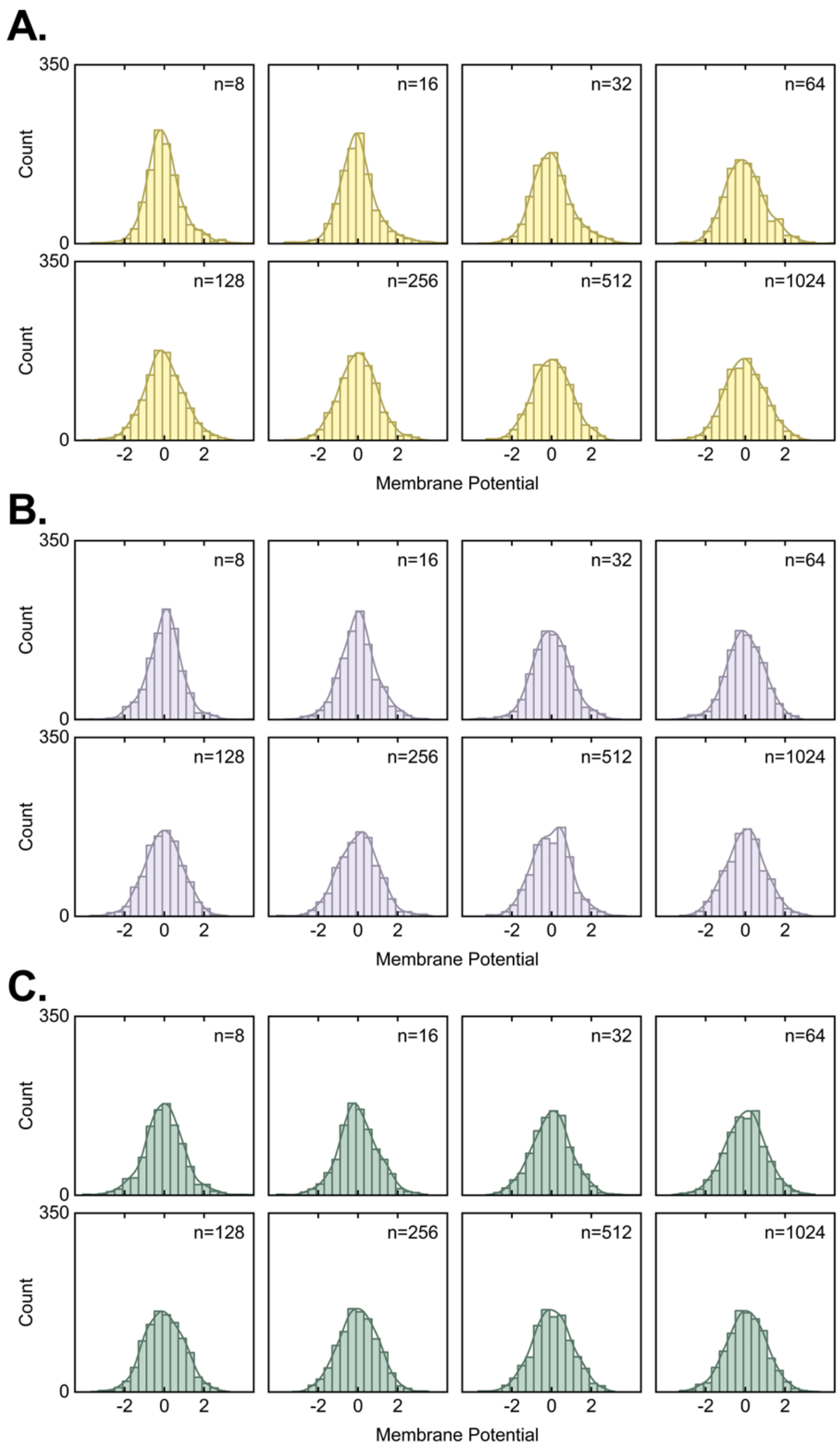

Figure S4: The distribution of membrane potential, where the uncertainty of the weights belongs to the non-exponential family distribution. In addition, the inputs are random in different samples. (A) Lévy Stable. (B) Student-T. (C) Symmetric Pareto.

Fig. S5.

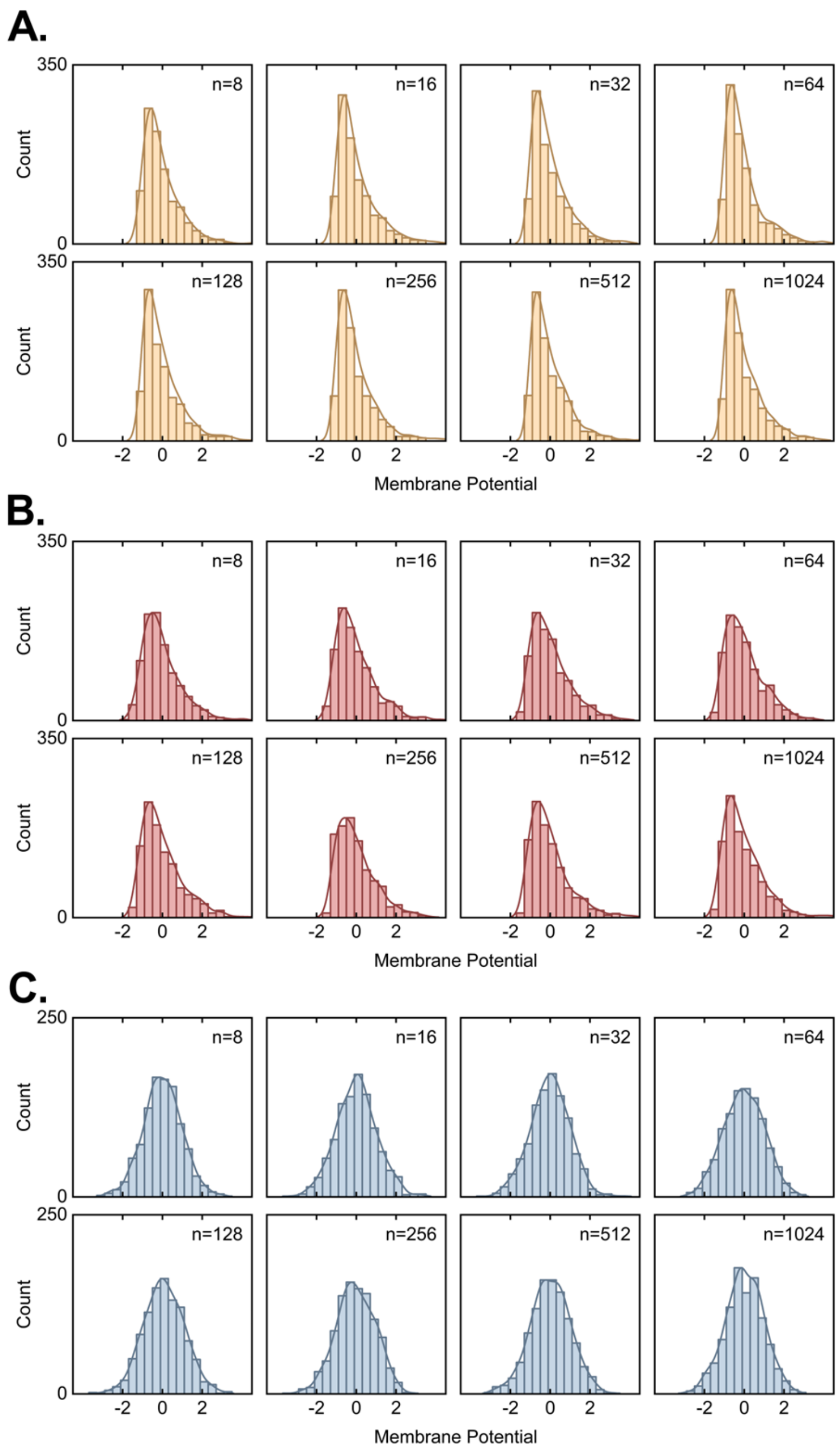

Figure S5: The distribution of membrane potential, where the uncertainty of the neurons belongs to the exponential family distribution. (A) Exponential. (B) Gamma. (C) Gaussian.

Fig. S6.

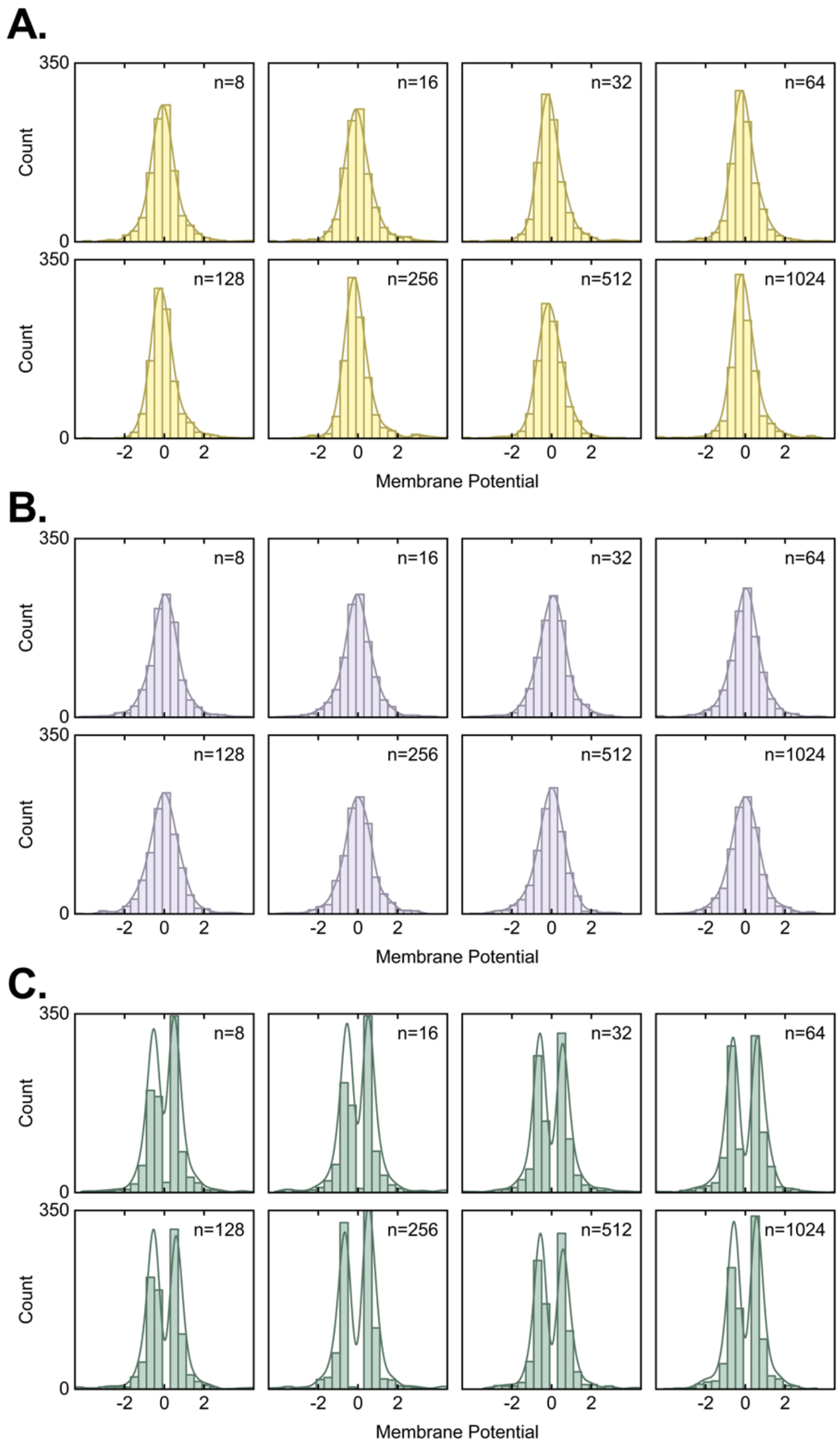

Figure S6: The distribution of membrane potential, where the uncertainty of the neurons belongs to the non-exponential family distribution. (A) Lévy Stable. (B) Student-T. (C) Symmetric Pareto.

Fig. S7.

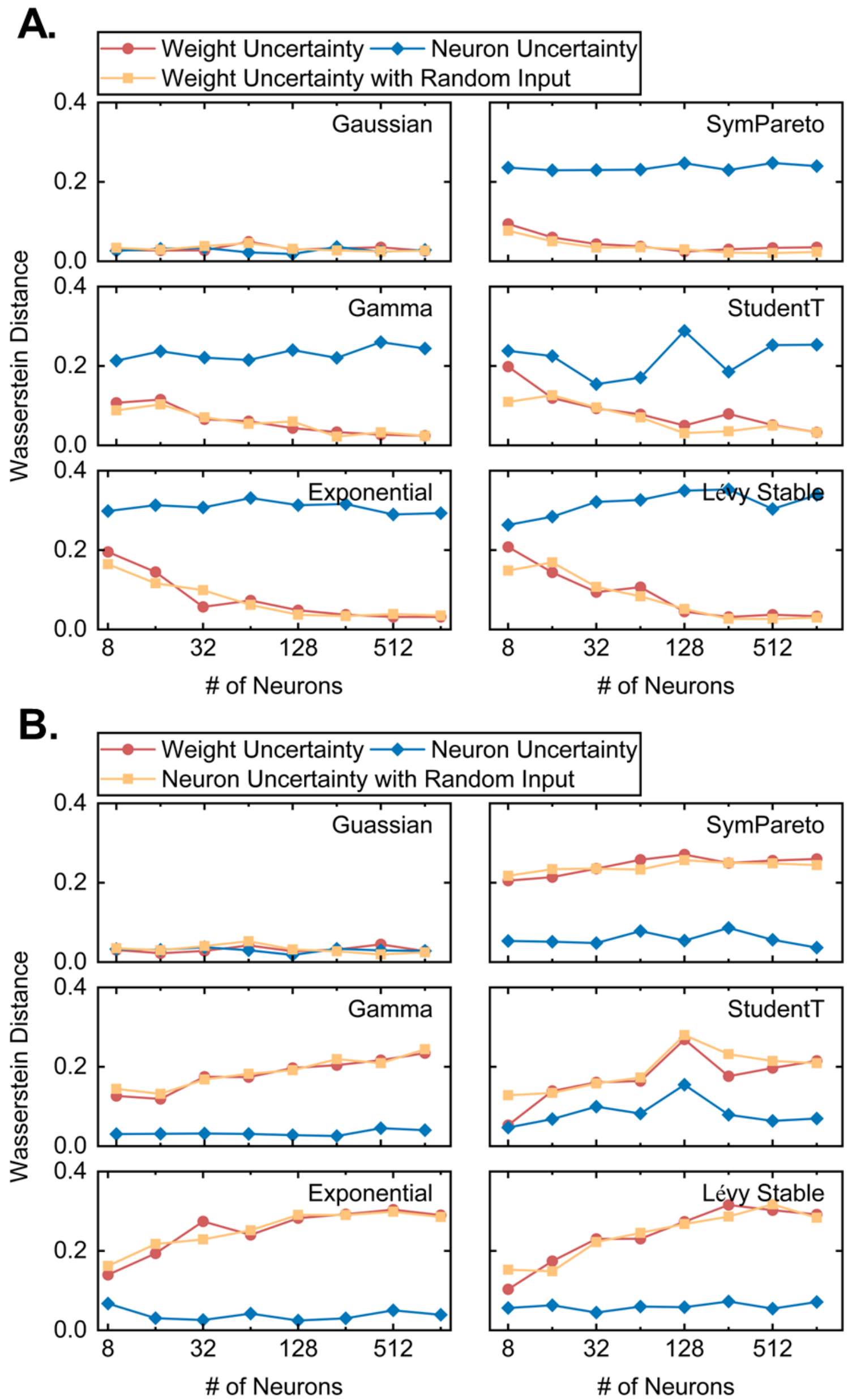

Figure S7: Across various number of neurons, the Wasserstein-1 distance between the distribution of membrane potential and the standard distribution or standard Gaussian distribution. (A) standard Gaussian distribution. (B) standard distribution.

Fig. S8.

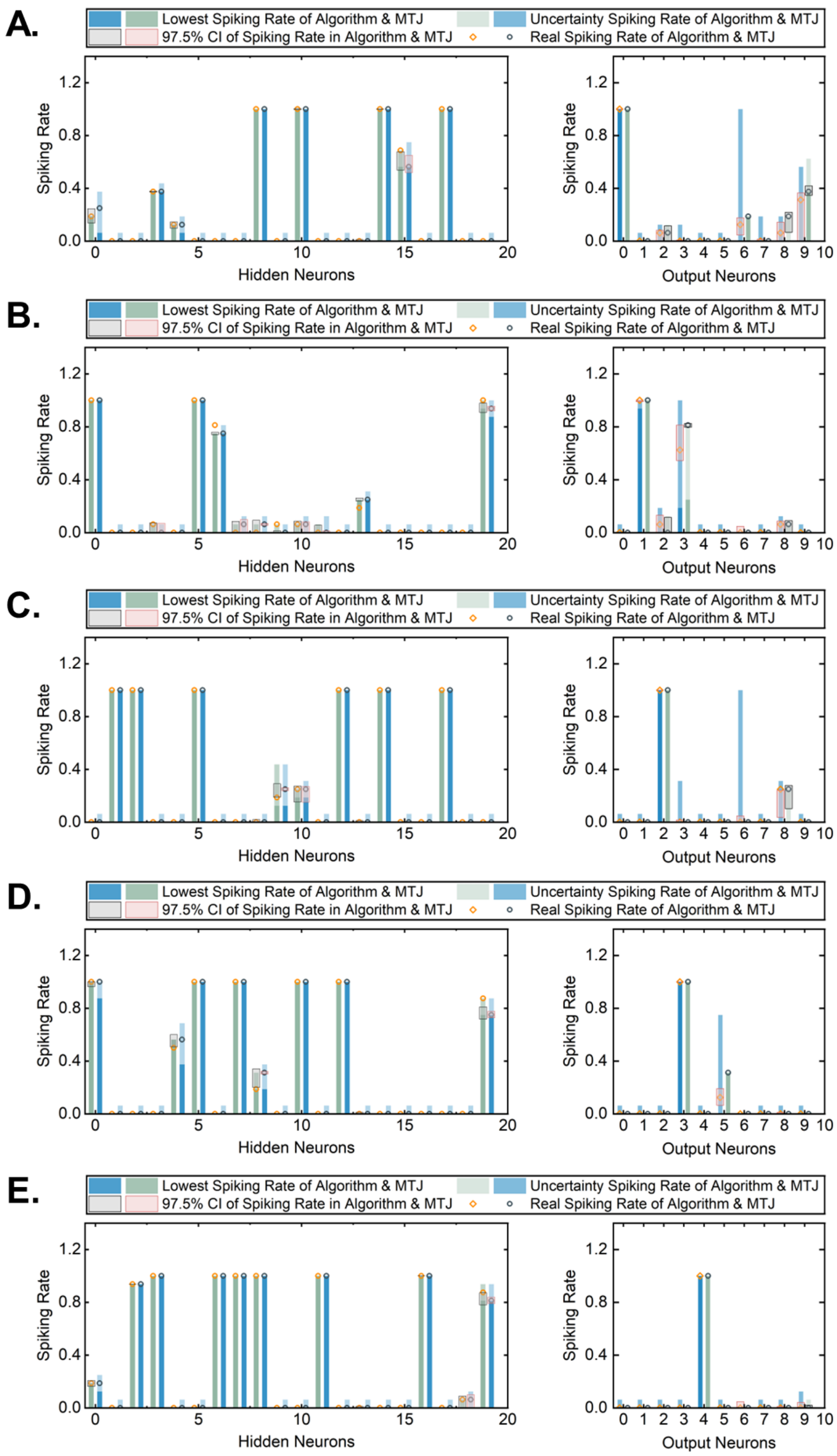

Figure S8: Hidden layer and Output layer spiking rates for MNIST test set predicted by the algorithm and measured on MTJ hardware. (A) Class 0, Sample 1565. (B) Class 1, Sample 191. (C) Class 2, Sample 298. (D) Class 3, Sample 2639. (E) Class 4, Sample 3792.

Fig. S9.

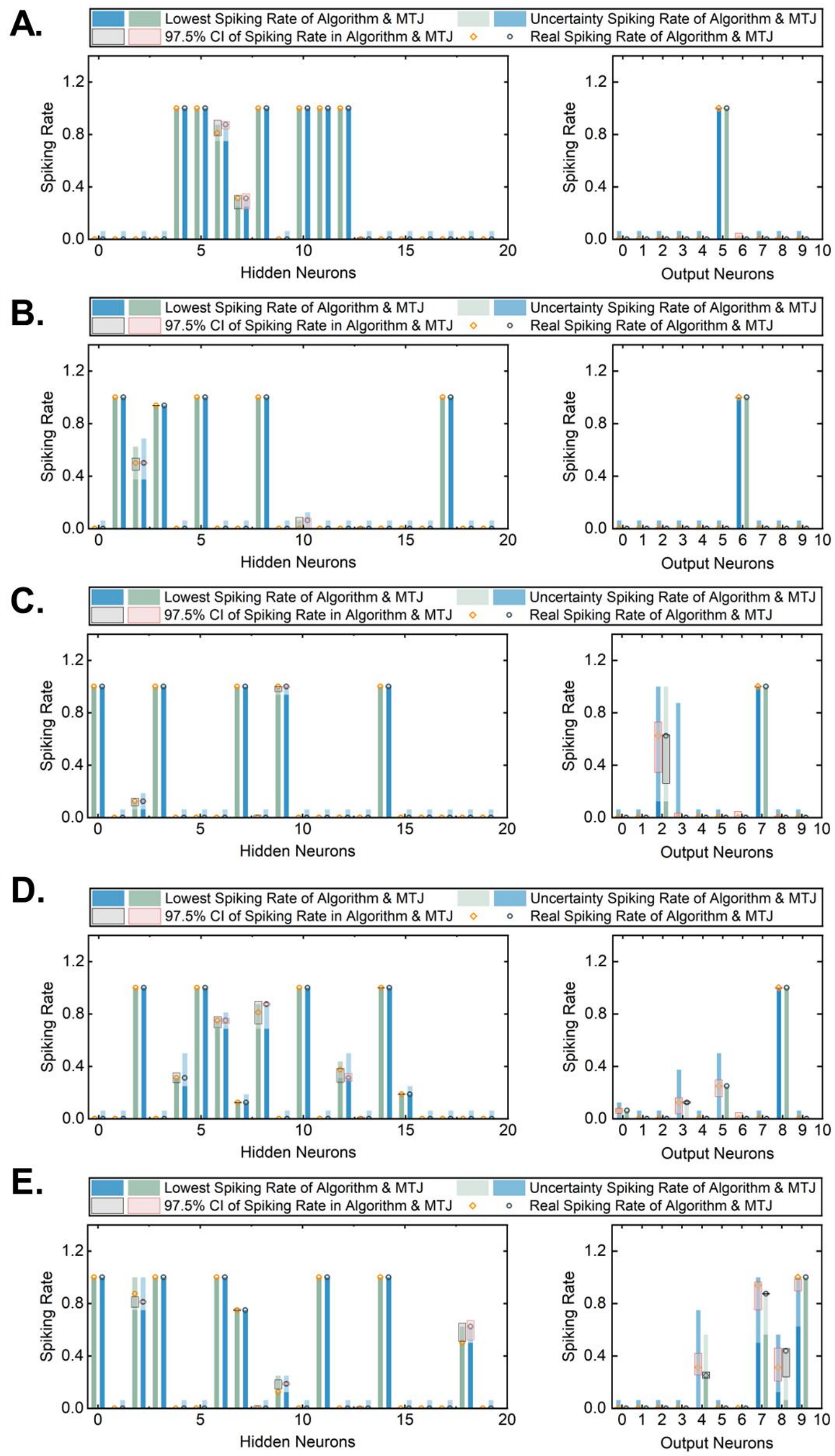

Figure S9: Hidden layer and Output layer spiking rates for MNIST test set predicted by the algorithm and measured on MTJ hardware. (A) Class 5, Sample 356. (B) Class 6, Sample 3331. (C) Class 7, Sample 617. (D) Class 8, Sample 714. (E) Class 9, Sample 193.

Fig. S10.

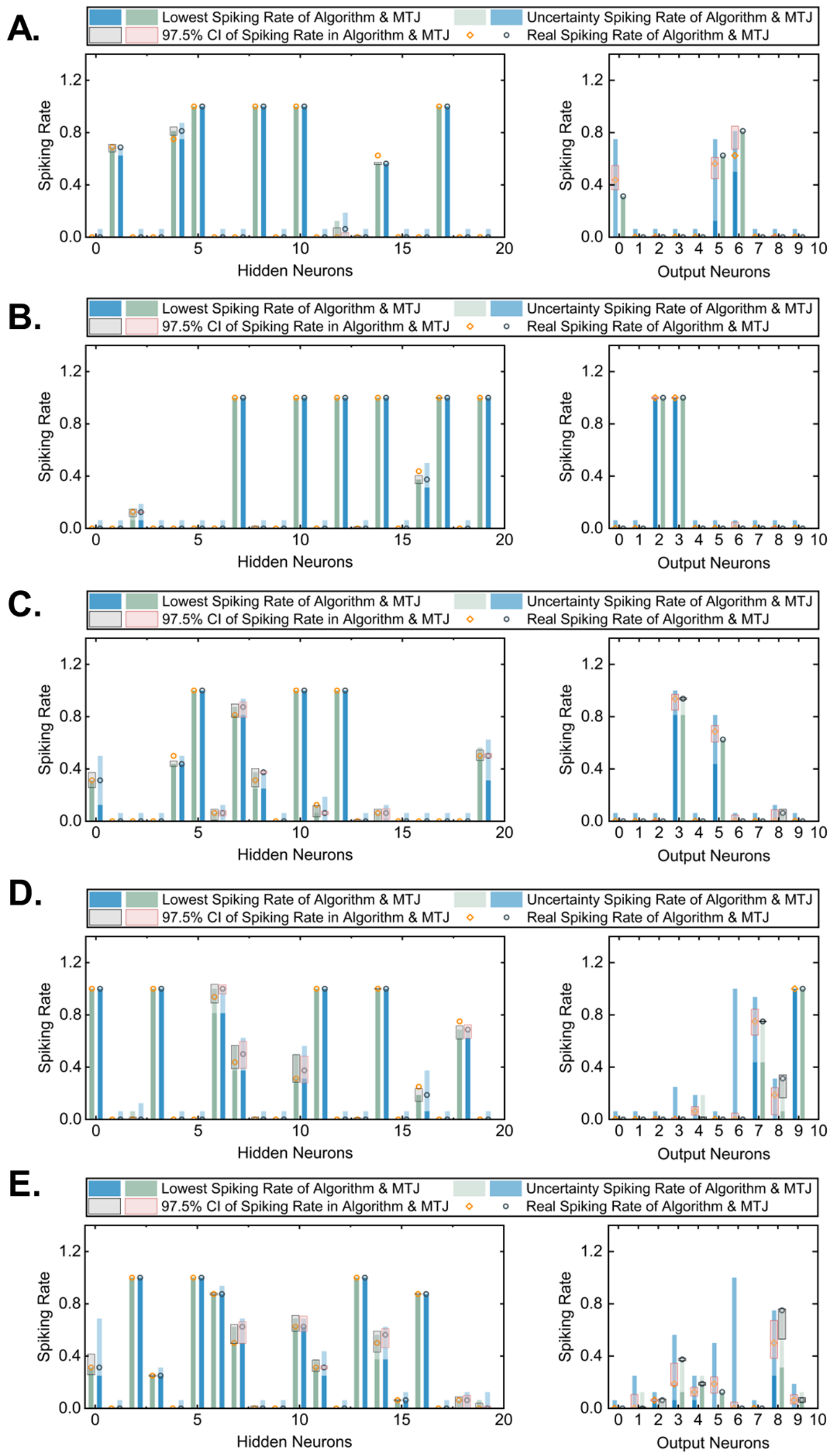

Figure S10: Hidden layer and Output layer spiking rates for MNIST test set predicted by the algorithm and measured on MTJ hardware (Error Samples). (A) Class 0, Sample 5255. (B) Class 3, Sample 4833. (C) Class 5, Sample 2832. (D) Class 7, Sample 5600. (E) Class 9, Sample 4761.

Fig. S11.

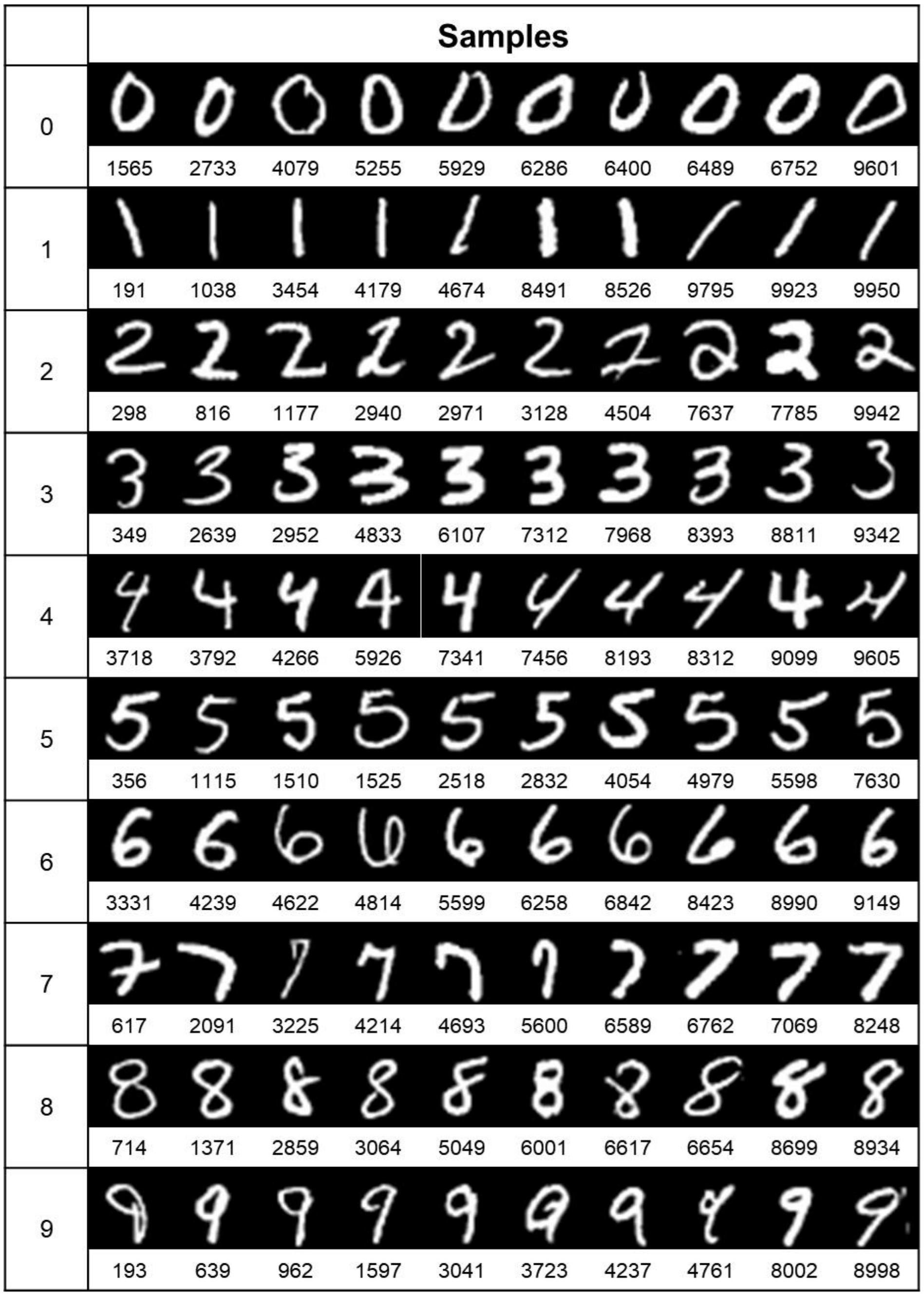

Figure S11: All samples of MNIST dataset in our hardware experiments.

Fig. S12.

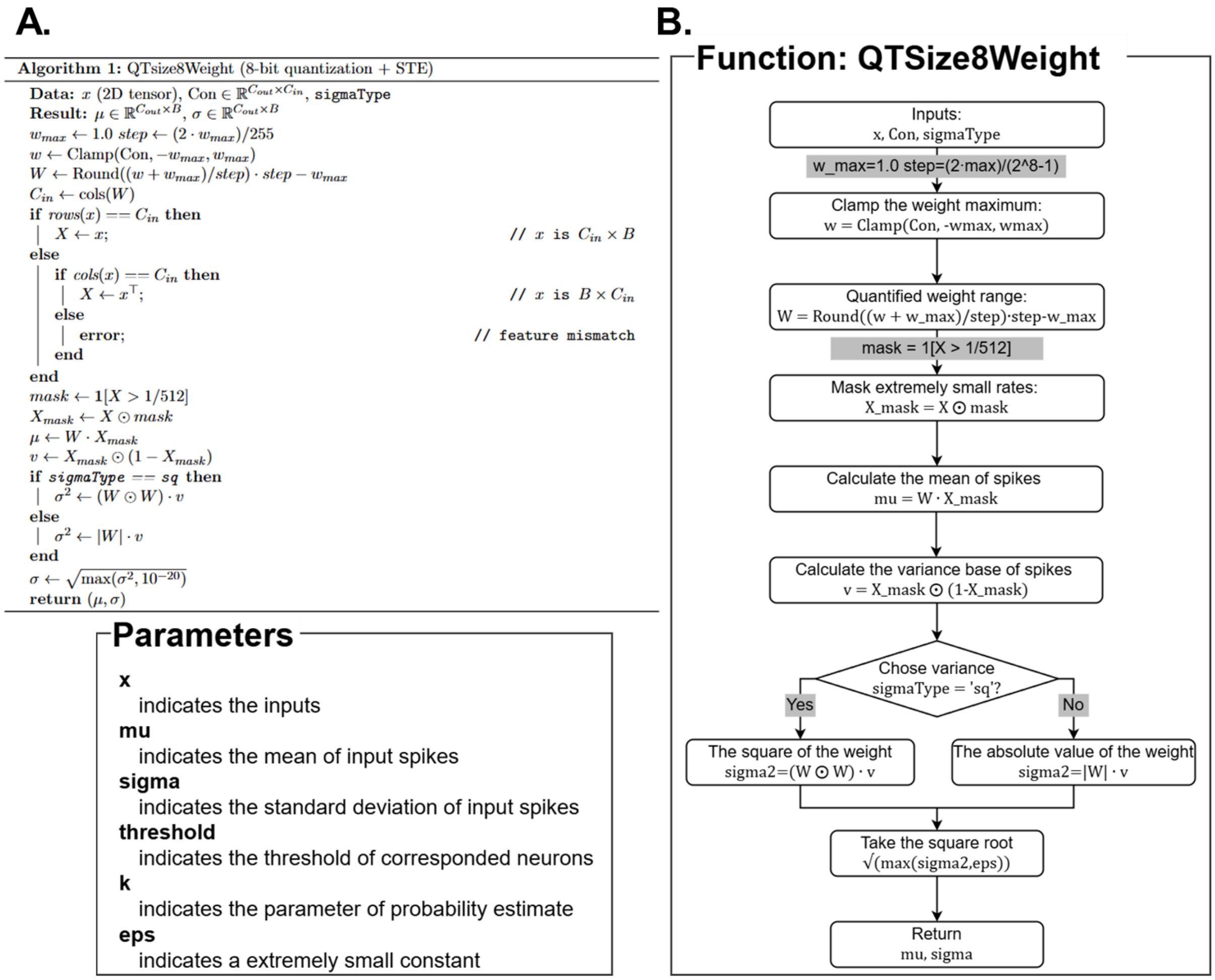

Figure S12: Forward and backward passes for weight quantization. (A) Pseudocode for QTsize8Weight. (B) The encapsulated QTsize8Weight, used for weight quantization and calculate the mean and standard deviation of input spikes for neurons in the linear layer.

Fig. S13.

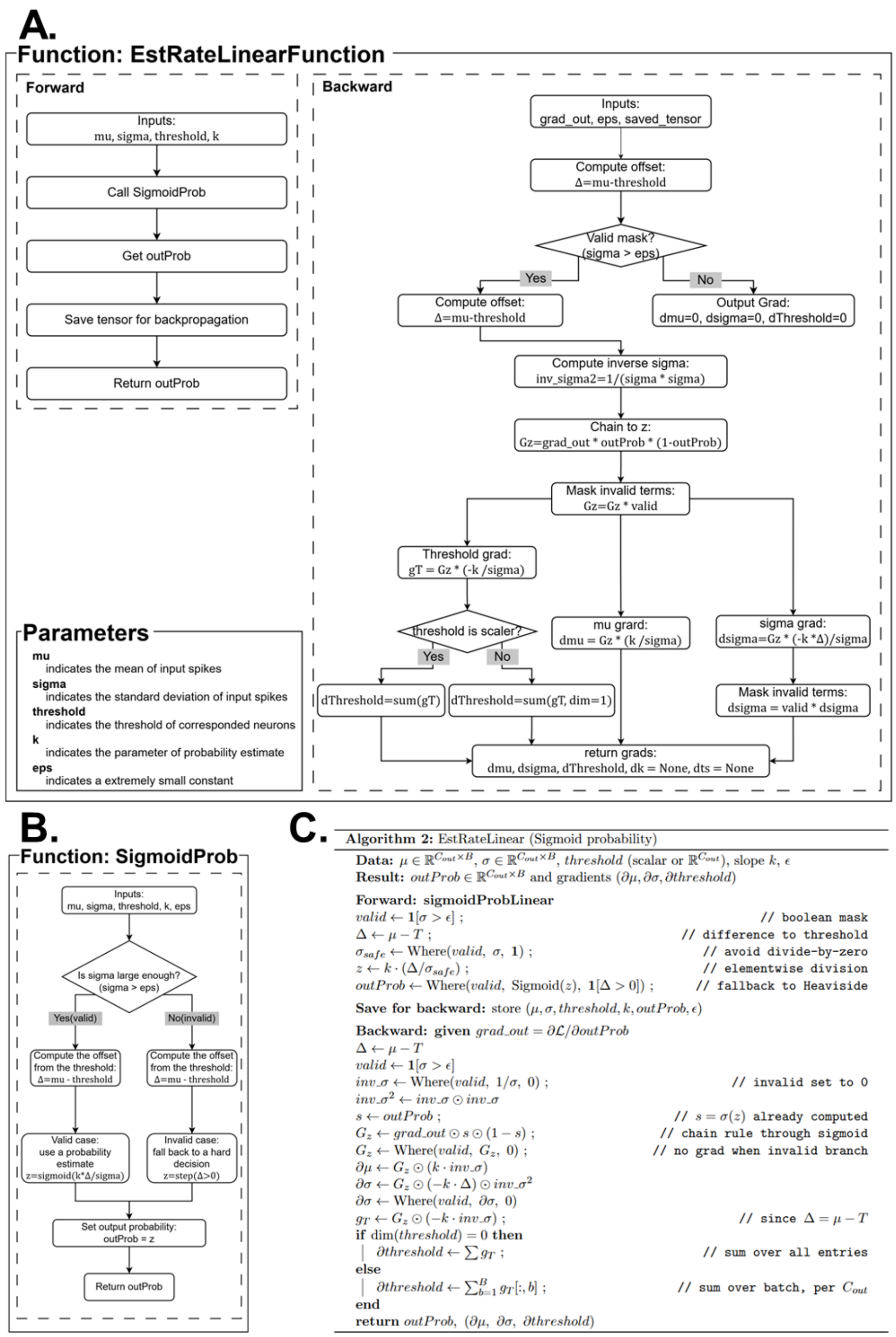

Figure S13: Forward and backward passes for rate estimation. (A) The encapsulated EstRateLinearFunction, used for rate estimation in the linear layer. (B) The main function for rate estimation, SigmoidProb. (C) Pseudocode for rate estimation.

Fig. S14.

A.

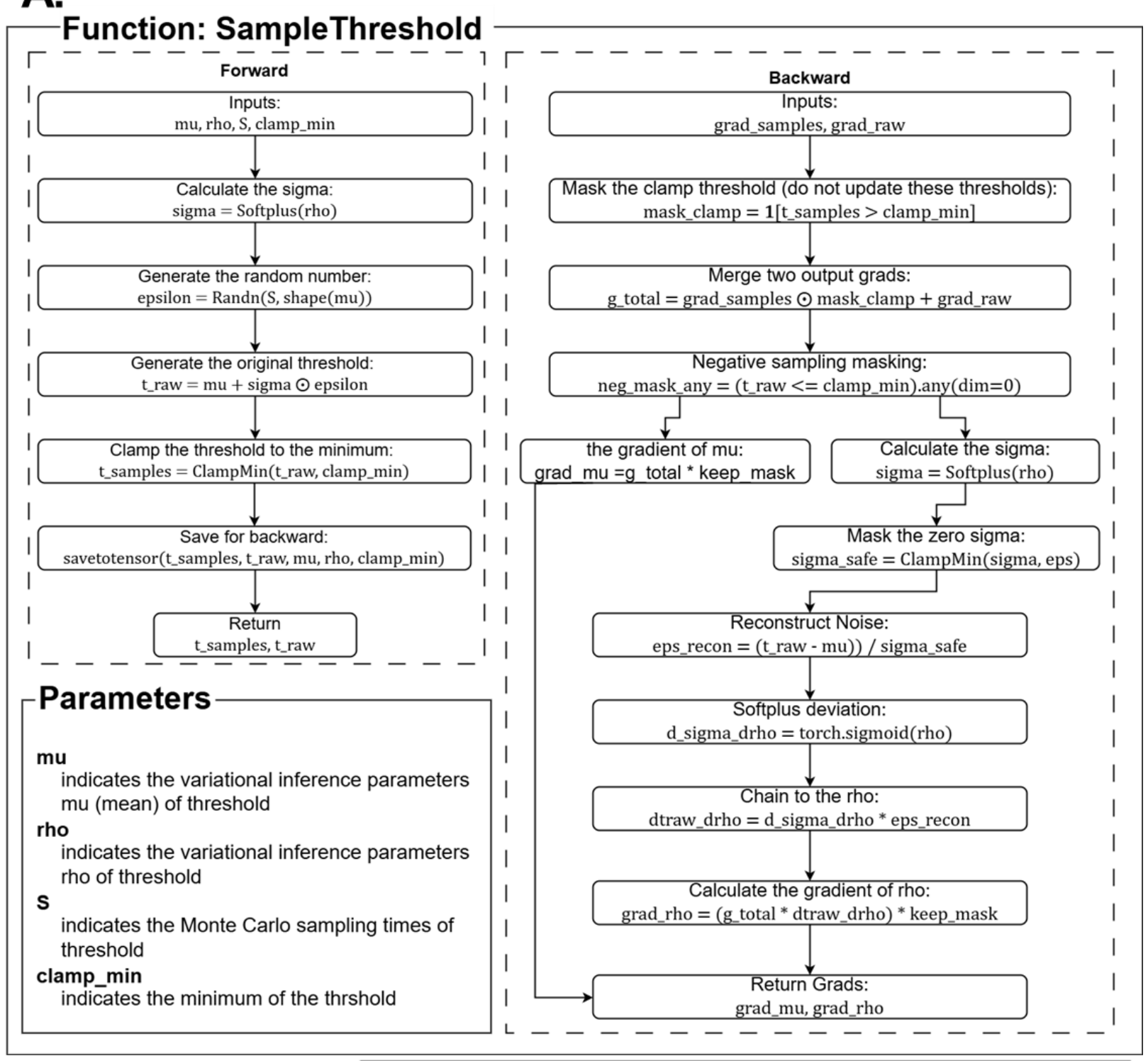

B.

Algorithm 3: SampleThreshold (MC sampling with clamp and any-negative masking)

Data: $\mu \in \mathbb{R}^{\cdots}$, $\rho \in \mathbb{R}^{\cdots}$, S (number of MC samples), $clamp\_min$ (scalar)
Result: $t\_samples \in \mathbb{R}^{S\times\cdots}$, $t\_raw \in \mathbb{R}^{S\times\cdots}$ and gradients $(\partial\mu, \partial\rho)$

Forward: SampleThresholdForward
$\sigma \leftarrow \mathrm{Softplus}(\rho)$; // σ has same shape as μ
$\epsilon \leftarrow \mathrm{Randn}(S,\ \mathrm{shape}(\mu))$; // Gaussian noise
$t\_raw \leftarrow \mu$ (broadcast to S) $+ \sigma$ (broadcast to S) $\odot \epsilon$; // reparameterization
$t\_samples \leftarrow \mathrm{ClampMin}(t\_raw,\ clamp\_min)$; // elementwise clamp

Save for backward: store $(t\_samples,\ t\_raw,\ \mu,\ \rho,\ clamp\_min)$

Backward: given $(grad\_samples,\ grad\_raw) = \left(\frac{\partial\mathcal{L}}{\partial t\_samples}, \frac{\partial\mathcal{L}}{\partial t\_raw}\right)$
if $grad\_samples$ *is None* **then**
| $grad\_samples \leftarrow \mathbf{0}$ (same shape as $t\_samples$)
end
if $grad\_raw$ *is None* **then**
| $grad\_raw \leftarrow \mathbf{0}$ (same shape as t_raw)
end
$mask\_clamp \leftarrow \mathbf{1}[t\_samples > clamp\_min]$; // clamp derivative gate
$g\_total \leftarrow grad\_samples \odot mask\_clamp + grad\_raw$; // merge two output grads
$neg\_any \leftarrow \mathrm{Any}(t\_raw \leq clamp\_min$ over dim $S)$; // shape: ...
$keep \leftarrow \mathbf{1}[\neg neg\_any]$; // mask out if any sample hits clamp
$\partial\mu \leftarrow (\sum\_s = 1^S g\_total[s,\ldots]) \odot keep$; // sum over samples, then mask

Compute $\partial\rho$**:**
$\sigma \leftarrow \mathrm{Softplus}(\rho)$
$\sigma\_safe \leftarrow \mathrm{ClampMin}(\sigma,\ \epsilon\_mach)$; // avoid divide-by-zero
$\epsilon\_recon \leftarrow \frac{t\_raw - \mu \text{ (broadcast to } S)}{\sigma\_safe \text{ (broadcast to } S)}$; // reconstruct noise
$\frac{d\sigma}{d\rho} \leftarrow \mathrm{Sigmoid}(\rho)$
$\frac{\partial t\_raw}{\partial\rho} \leftarrow \left(\frac{d\sigma}{d\rho} \text{ (broadcast to } S)\right) \odot \epsilon\_recon$
$\partial\rho \leftarrow \left(\sum_{s=1}^{S} g\_total[s,\ldots] \odot \frac{\partial t\_raw[s,\ldots]}{\partial\rho}\right) \odot keep$
return $(t\_samples,\ t\_raw),\ (\partial\mu,\ \partial\rho)$

Figure S14: Forward and backward passes for SampleThreshold. (A) The encapsulated SampleThreshold, used for sampling the threshold at each forward. (B) Pseudocode for SampleThreshold.

Fig. S15.

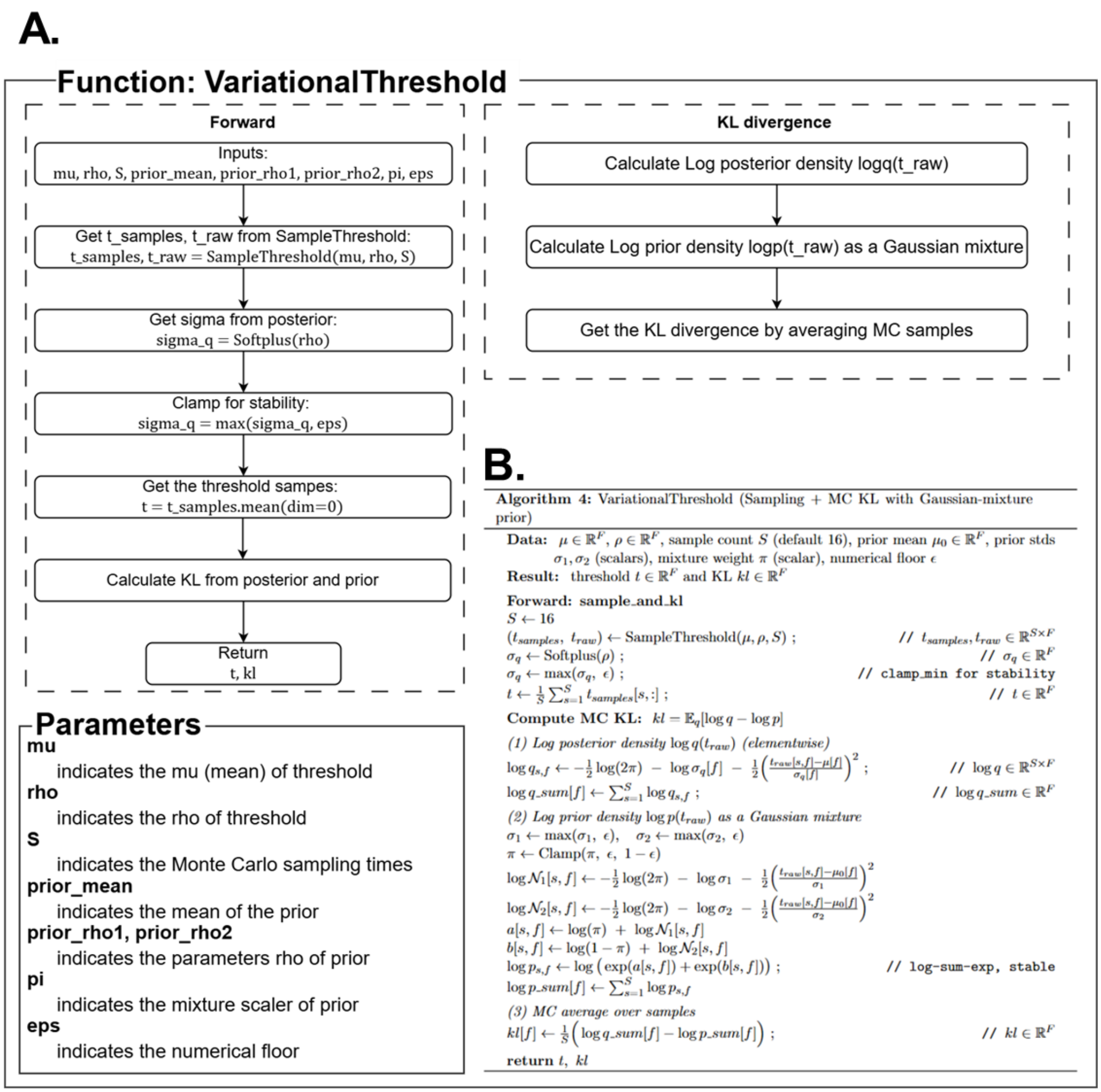

Figure S15: Forward passes for VariationalThreshold. (A) The encapsulated VariationalThreshold, used for sampling the threshold and calculating the KL divergence at each forward. (B) Pseudocode for VariationalThreshold.

Fig. S16.

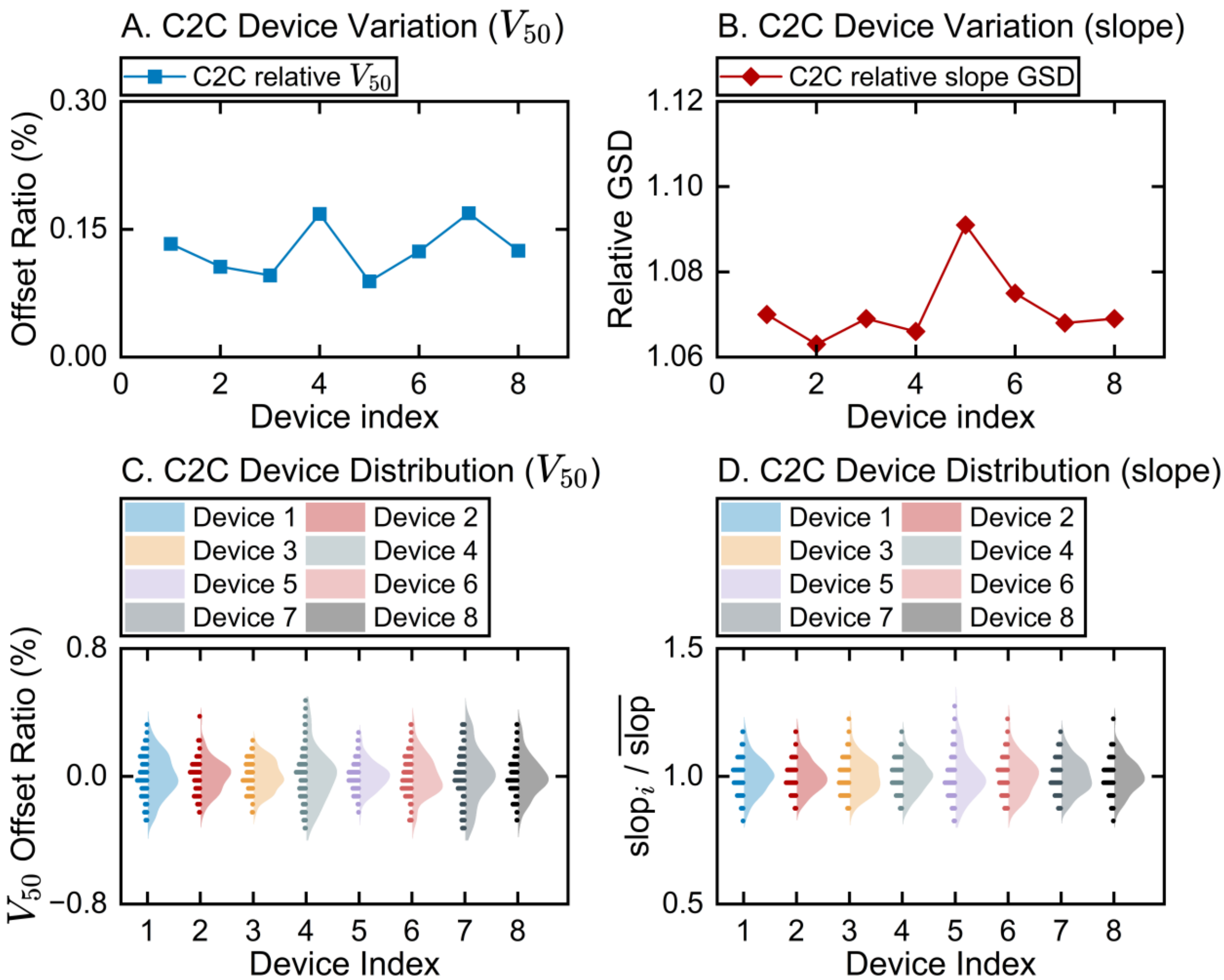

Figure S16: Repeatability of switching-probability characteristics across eight MTJs. (A) Coefficient of variation of the switching center, (V_{50}). (B) Geometric standard deviation of the dimensionless slope factor, (k). (C) Distribution of variation of the switching center, (V_{50}). (D) Distribution of slope factor ($k_i/\bar{k}$).

Fig. S17.

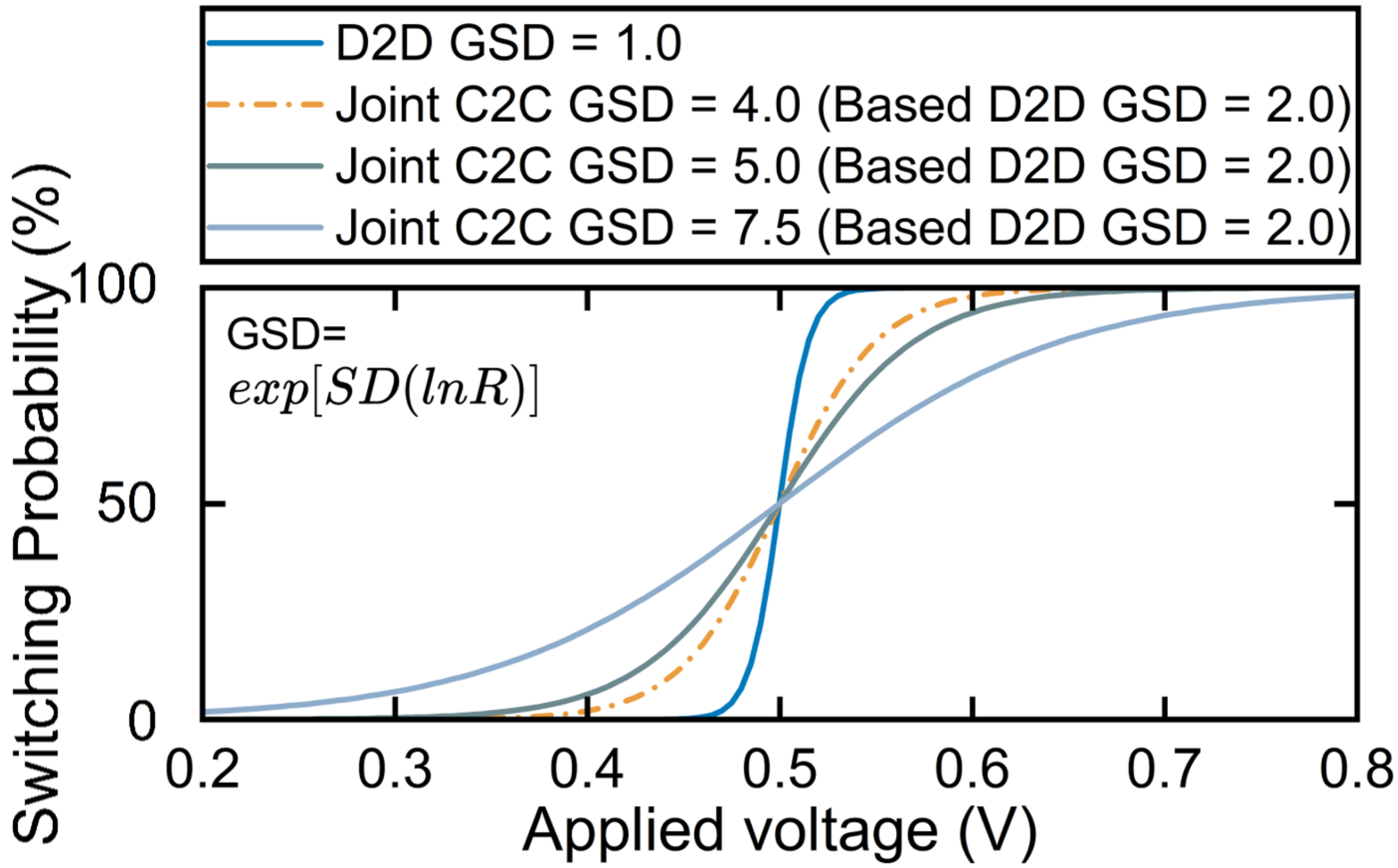

Figure S17: Representative MTJ switching curves under temporal slope variability. The nominal switching curve is compared with illustrative curves for C2C slope GSDs of 4.0, 5.0, and 7.5 on a static D2D background with a nominal slope GSD of 2.0. GSD is defined as exp[SD(ln R)], where R denotes the corresponding positive slope multiplier. The switching model and variability parameterization are described in Section 4.12.2–4.12.3.

Fig. S18.

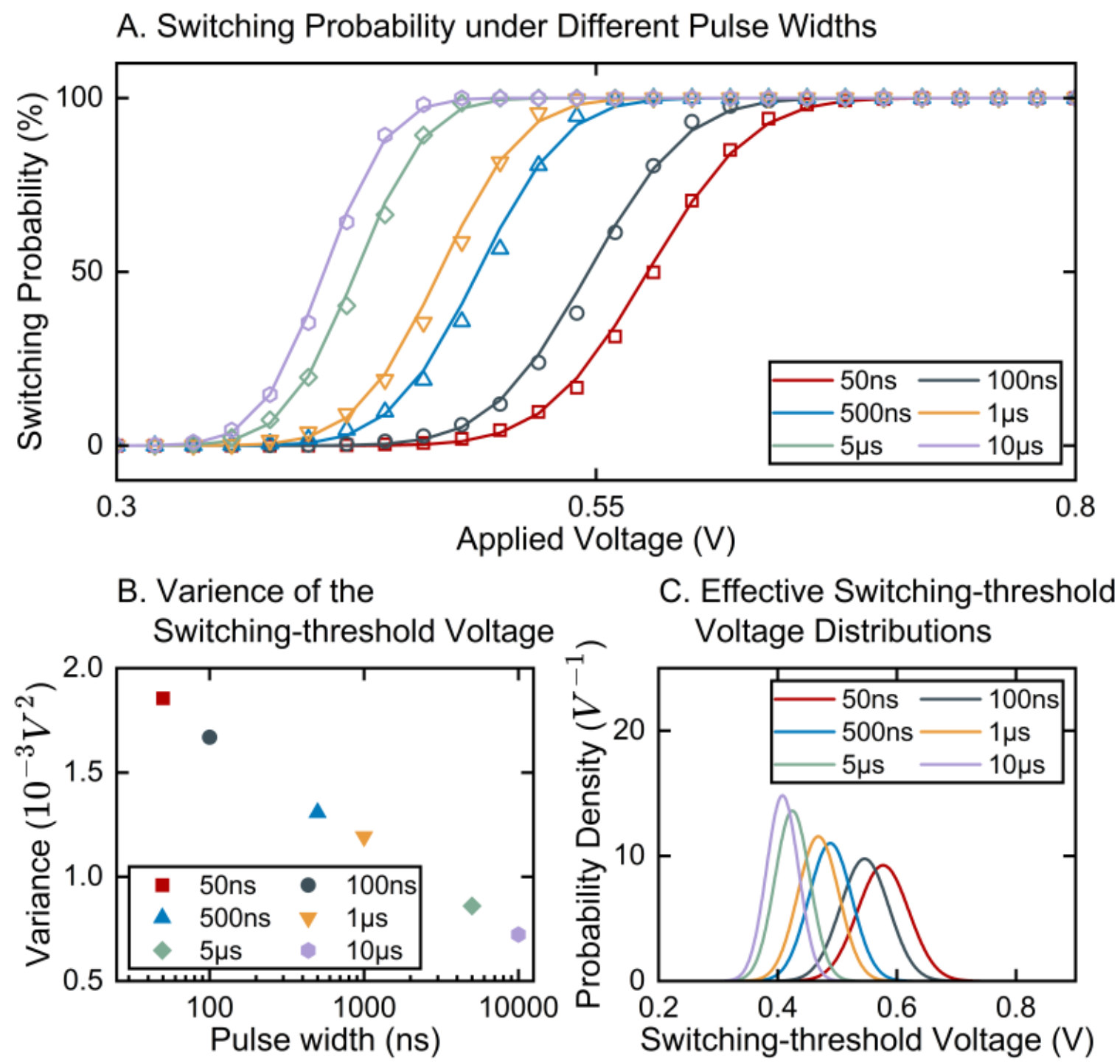

Figure S18: Pulse-width dependence of MTJ switching statistics. (Device width: 200nm) (A) Switching probability as a function of applied voltage at pulse widths of 50 ns, 100 ns, 500 ns, 1 μs, 5 μs, and 10 μs. Symbols represent measured switching probabilities, and solid lines represent fits obtained by binomial maximum likelihood estimation. (B) Variance of the effective switching-threshold voltage distributions shown in (C), plotted against pulse width on a logarithmic horizontal axis. (C) Effective switching-threshold voltage probability densities obtained by differentiating the fitted switching-probability curves in (A), $f(V; t_p) = \partial\hat{P}_{\mathrm{sw}}(V; t_p)/\partial V$, where t_p denotes the pulse width and $\hat{P}_{\mathrm{sw}}$ is the fitted switching probability expressed between 0 and 1. Increasing the pulse width shifts the switching response toward lower voltages and narrows the effective threshold distribution over the investigated range.

Fig. S19.

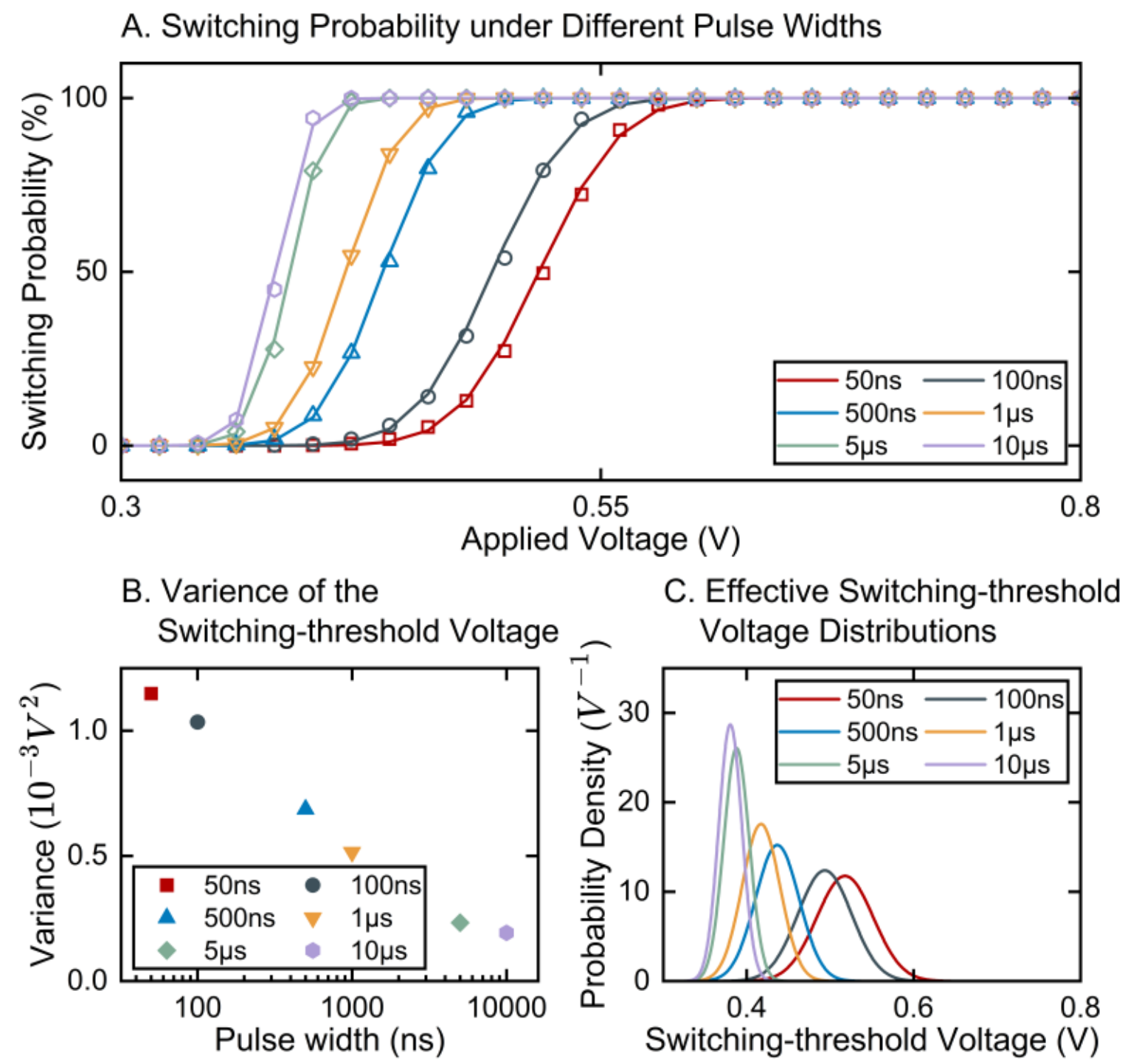

Figure S19: Pulse-width dependence of MTJ switching statistics. (Device width: 150nm) (A) Switching probability as a function of applied voltage at pulse widths of 50 ns, 100 ns, 500 ns, 1 μs, 5 μs, and 10 μs. Symbols represent measured switching probabilities, and solid lines represent fits obtained by binomial maximum likelihood estimation. (B) Variance of the effective switching-threshold voltage distributions shown in (C), plotted against pulse width on a logarithmic horizontal axis. (C) Effective switching-threshold voltage probability densities obtained by differentiating the fitted switching-probability curves in (A), $f(V; t_p) = \partial\hat{P}_{\mathrm{sw}}(V; t_p)/\partial V$, where t_p denotes the pulse width and $\hat{P}_{\mathrm{sw}}$ is the fitted switching probability expressed between 0 and 1. Increasing the pulse width shifts the switching response toward lower voltages and narrows the effective threshold distribution over the investigated range.

Fig. S20.

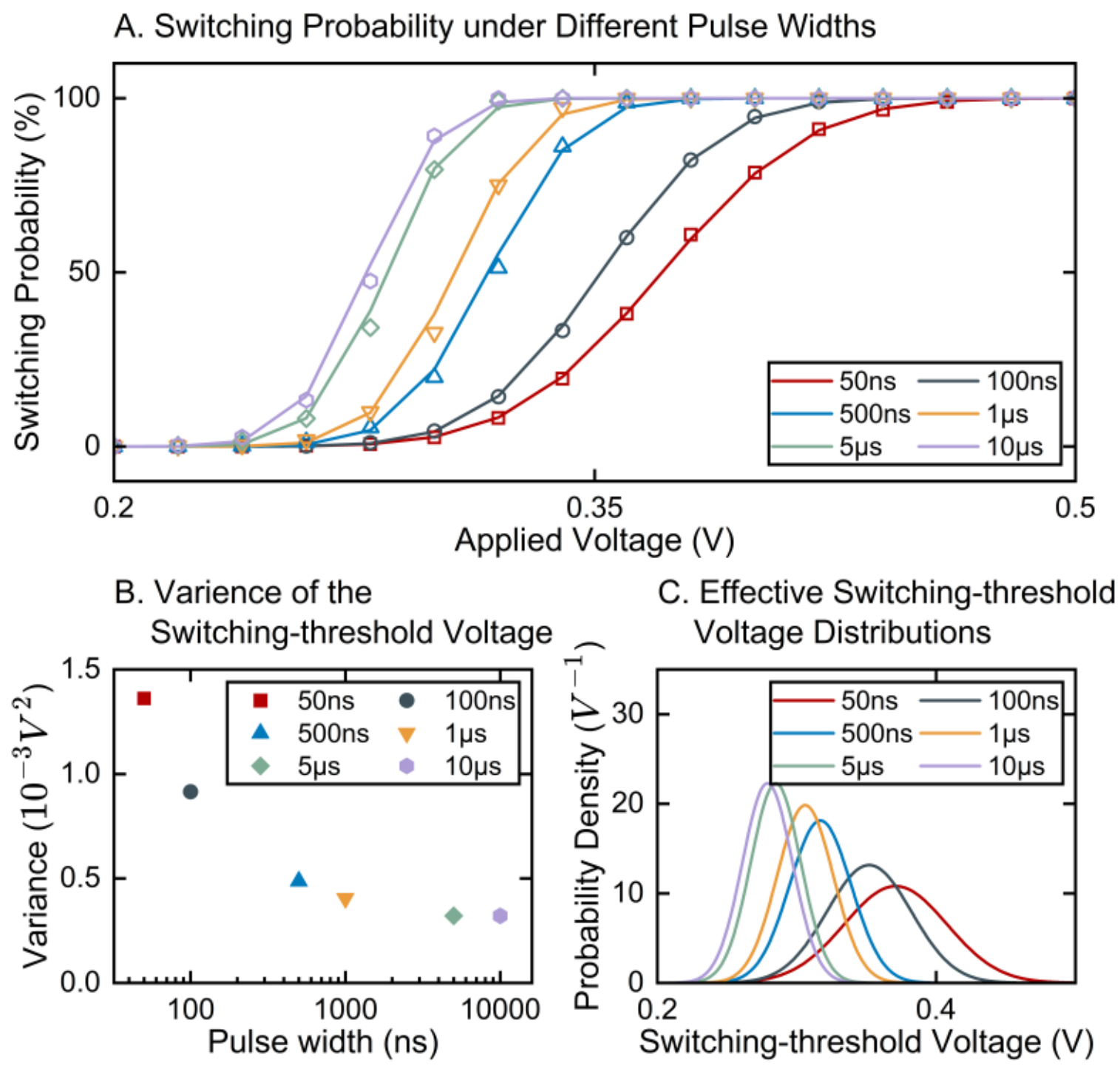

Figure S20: Pulse-width dependence of MTJ switching statistics. (Device width: 100nm) (A) Switching probability as a function of applied voltage at pulse widths of 50 ns, 100 ns, 500 ns, 1 μs, 5 μs, and 10 μs. Symbols represent measured switching probabilities, and solid lines represent fits obtained by binomial maximum likelihood estimation. (B) Variance of the effective switching-threshold voltage distributions shown in (C), plotted against pulse width on a logarithmic horizontal axis. (C) Effective switching-threshold voltage probability densities obtained by differentiating the fitted switching-probability curves in (A), $f(V;t_p) = \partial\hat{P}_{\mathrm{sw}}(V;t_p)/\partial V$, where t_p denotes the pulse width and $\hat{P}_{\mathrm{sw}}$ is the fitted switching probability expressed between 0 and 1. Increasing the pulse width shifts the switching response toward lower voltages and narrows the effective threshold distribution over the investigated range.

Fig. S21.

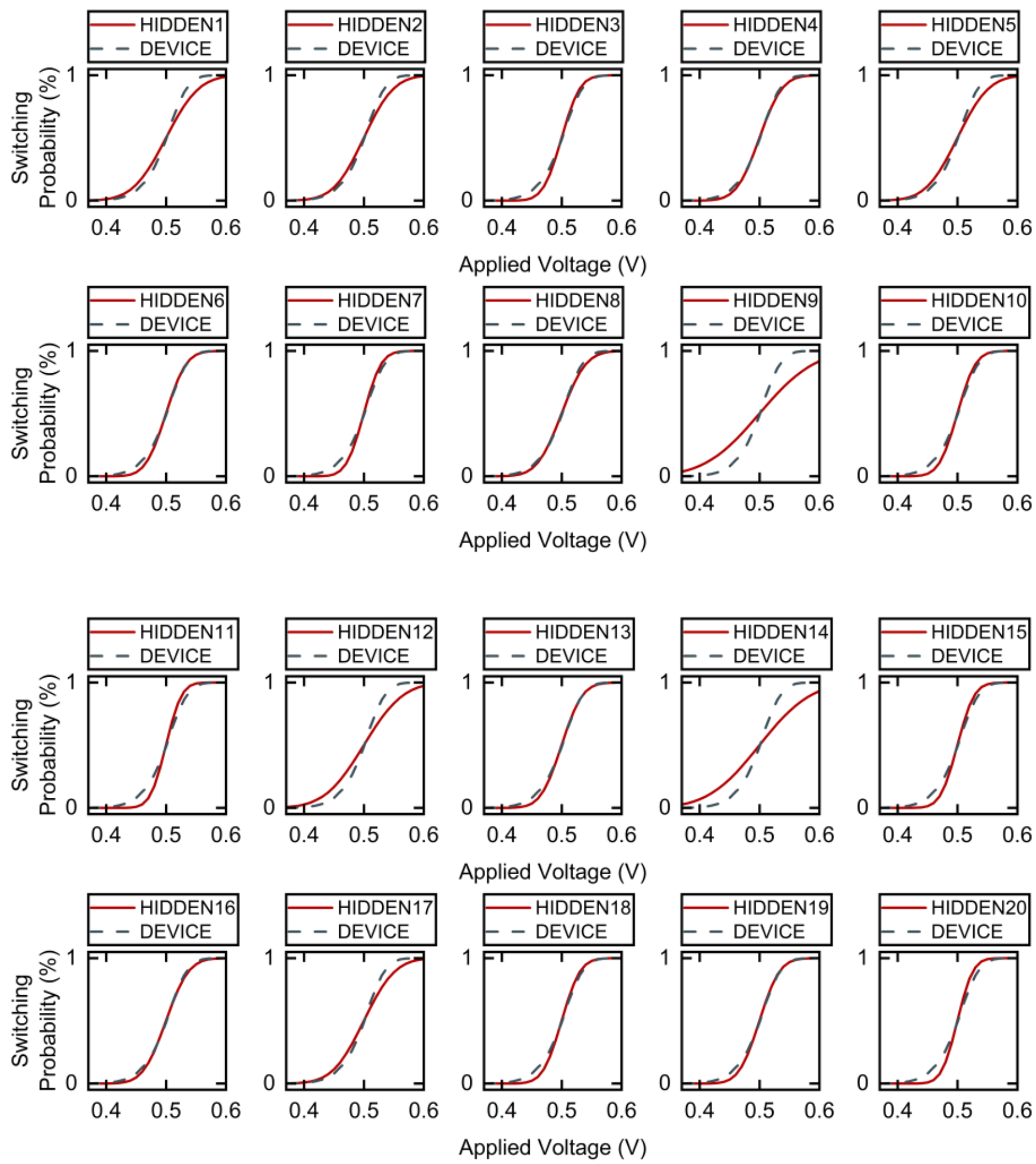

Figure S21: Comparison of algorithmic and measured device switching probabilities for hidden neurons. Algorithmic firing-probability curves for the 20 hidden neurons are calculated from their Gaussian threshold cumulative distribution functions and expressed in the applied-voltage coordinate using the inverse of the input mapping in Eq. (2). Specifically, $\left(P_{\mathrm{alg},i}(V) = \Phi[(\mu_i V/V_{50} - \mu_i)/\sigma_i]\right)$, where ($\Phi$) is the standard Gaussian cumulative distribution function, (μ_i) and (σ_i) are the algorithmic threshold mean and standard deviation, and (V_{50}) is the reference MTJ switching midpoint. The device curves show experimentally measured switching probabilities over 0.37–0.60 V. Probabilities are expressed on a 0–1 scale.

Fig. S22.

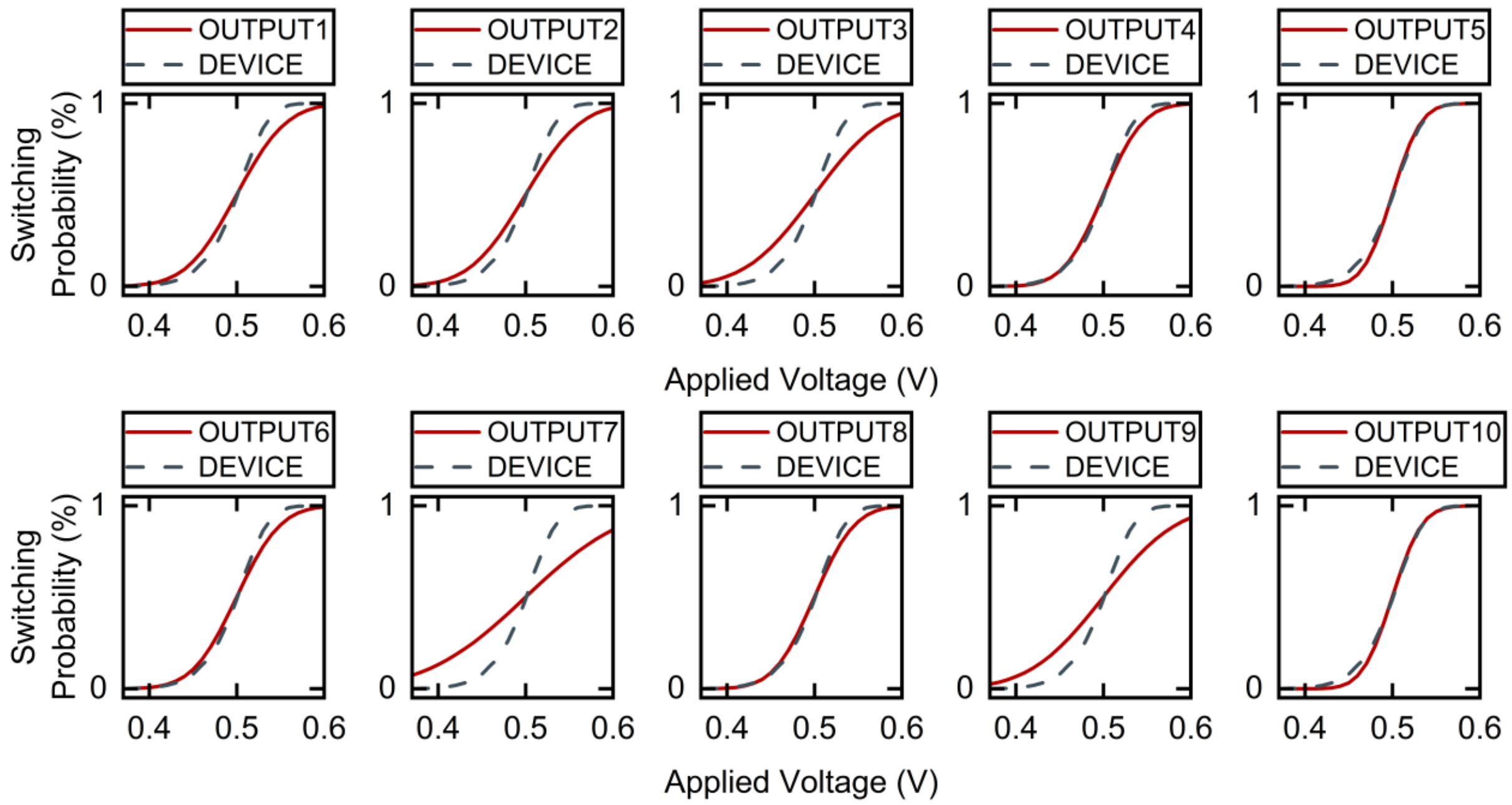

Figure S22: Comparison of algorithmic and measured device switching probabilities for output neurons. Gaussian-CDF firing-probability curves for the 10 output neurons are compared with experimentally measured MTJ switching probabilities over 0.37–0.60 V. Algorithmic curves are expressed in the applied-voltage coordinate using the inverse of Eq. (2), following the same procedure as in Fig. S21. Probabilities are expressed on a 0–1 scale.

Fig. S23.

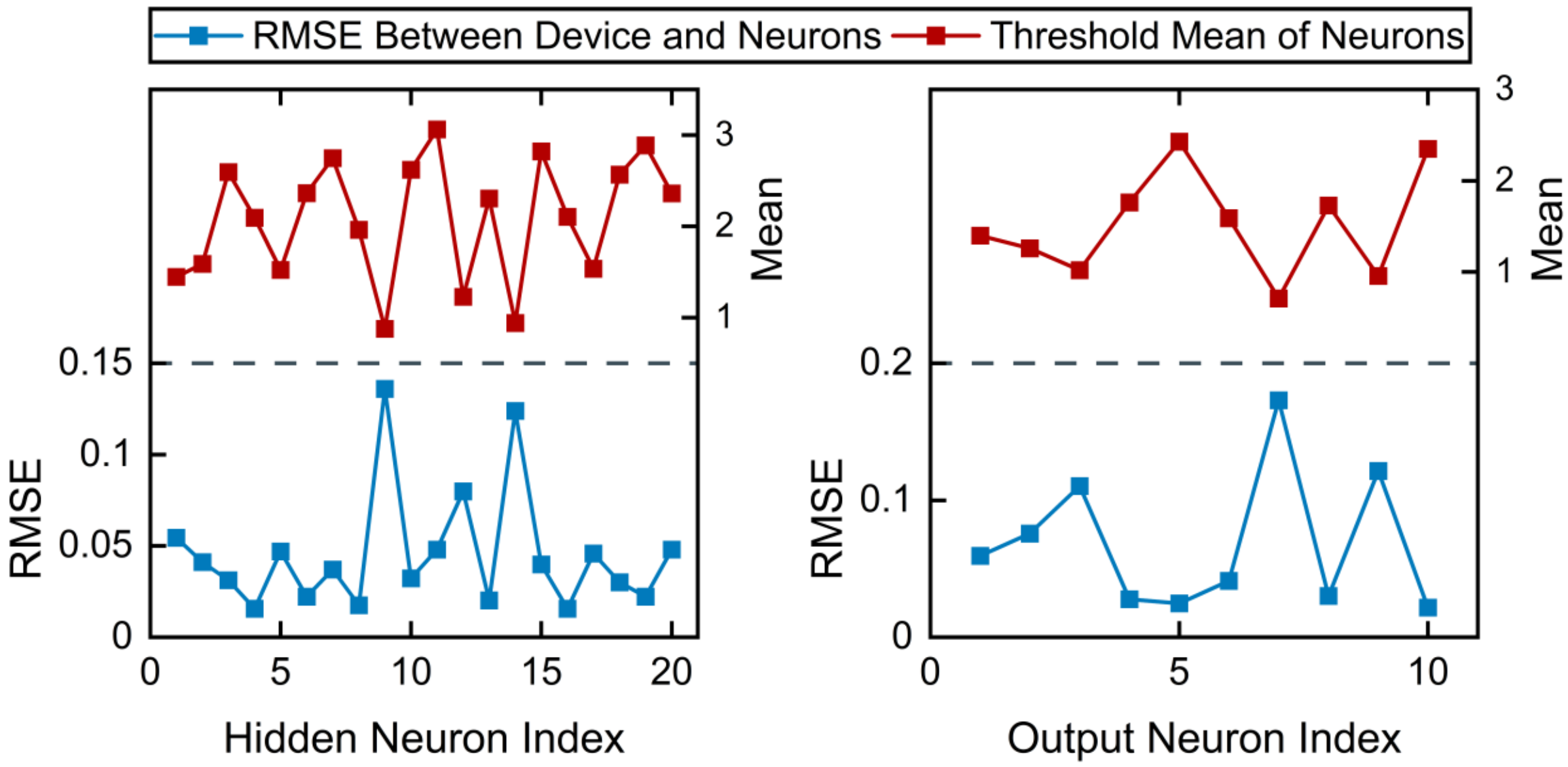

Figure S23: Neuron-wise mismatch between algorithmic and measured device switching probabilities. Blue curves show the probability-curve RMSE for hidden neurons (left panel) and output neurons (right panel): RMSE$i = \sqrt{\frac{1}{M}\sum m = 1^M \left[P_{\mathrm{alg},i}(V_m) - P_{\mathrm{dev}}^{\mathrm{meas}}(V_m)\right]^2}$. Here, (V_m) denotes a measured voltage point within 0.37–0.60 V, and (M) is the number of measured voltage points. $(P_{\mathrm{alg},i}(V_m))$ is calculated from the Gaussian threshold CDF using the inverse input mapping described in Fig. S21, and $(P_{\mathrm{dev}}^{\mathrm{meas}}(V_m))$ is the experimentally measured switching probability rather than a fitted sigmoid value. Both probabilities are expressed on a 0–1 scale and evaluated at the same voltage points. Red curves show the corresponding algorithmic threshold means. RMSE and threshold mean refer to the left and right axes, respectively.

S3. Supplementary Table

Table S1: Accuracy of SBNN in Recognition Tasks (%)

Data Set	Weight bits	ρ	Time Steps					
			2	3	4	6	8	16
MNIST	8	-0.5	98.77	98.90	99.04	99.09	99.16	99.15
		0.0	98.80	98.88	98.99	99.08	99.14	99.16
		0.5	98.79	98.99	99.02	99.09	99.18	99.14
	1	-0.5	97.89	98.12	98.32	98.51	98.56	98.71
		0.0	97.75	98.15	98.35	98.37	98.53	98.57
		0.5	97.72	98.22	98.30	98.43	98.54	98.62
Fashion MNIST	8	-0.5	86.38	87.79	88.20	89.20	89.44	90.05
		0.0	86.79	87.50	88.30	88.98	89.48	89.95
		0.5	85.92	87.70	88.38	89.00	89.45	89.84
	1	-0.5	82.67	84.47	84.79	85.66	86.20	86.71
		0.0	83.48	84.57	85.94	86.39	86.49	87.31
		0.5	83.27	84.43	85.78	86.33	86.53	87.16
Cifar10	Mix	-0.5	94.37	94.41	94.22	94.37	94.29	94.58
		0.0	94.23	94.26	94.16	94.35	94.62	94.39
		0.5	94.64	94.39	94.61	94.65	94.84	94.70

Table S2: Experiment Setting of Image Recognition Tasks

	MNIST	F-MNIST	Cifar10
Optimizer	adamw		
Batch Size	64	64	64
Epochs	500	2000	1000
Sigmoid Scale Factor (K)	1.716	1.716	1.716
Threshold Initialization	1.0	1.0	2.0
Minium of Threshold	1/128		
Learning Rate (Threshold)	1e-5	7.5e-4	1e-5
Weight Quantization Range	[-1, 1]		
Learning Rate (Weight)	5e-4	5e-4	5e-3
KL Beta	1e-3	1e-5	5e-8
Loss Function	Cross-Entropy		
Learning Rate Scheduler	Cosine Annealing LR		
Weight Decay	1e-3		
Scheduler Max Epochs	500	1000	1000
Prior Mu Initialization (Mean)	3.0	3.0	3.0
Prior Mu Initialization (Std)	2.9	1.5	1.5
Std of Prior1	0.5	1.625	0.5
Std of Prior2	0.05	0.05	0.05
Mixture Ratio of Prior	0.5		
Seed	42		
Random Crop	(28, 1)		(32, 4)
Random Horizontal Flip	False		True
Cutout	False		1/16
Warm Up Epochs	0		20

Table S3: Device Switching Probability. For all sweeping voltages, we applied 10,000 independent times to the device and read out the device resistance after applying voltages.

Voltage (V)	Parallel (# of Pulse)	Anti-Parallel (# of Pulse)	Switching Probability
0.6	10000	0	1
0.59	10000	0	1
0.58	9999	1	0.9999
0.57	9979	21	0.9979
0.56	9903	97	0.9903
0.55	9711	289	0.9711
0.54	9329	671	0.9329
0.53	8634	1366	0.8634
0.52	7613	2387	0.7613
0.51	6373	3627	0.6373
0.50	4899	5101	0.4899
0.49	3674	6326	0.3674
0.48	2642	7358	0.2642
0.47	1750	8250	0.175
0.46	1296	8704	0.1296
0.45	805	9195	0.0805
0.44	501	9499	0.0501
0.43	310	9690	0.031
0.42	190	9810	0.019
0.41	106	9894	0.0106
0.4	42	9958	0.0042
0.39	28	9972	0.0028
0.38	9	9991	0.0009
0.37	1	9999	0.0001

Table S4: Spiking Rate of Each Neuron in Algorithmic Model and Device Implementation (Class 0 & Class 1)

Class	Type	Sample	Neuron									
			0	1	2	3	4	5	6	7	8	9
0	Algorithm	1565	**1.00**	0.00	0.06	0.00	0.00	0.00	0.13	0.00	0.06	0.31
		2733	**1.00**	0.00	0.00	0.00	0.00	0.00	0.13	0.00	0.13	0.06
		4079	**0.94**	0.00	0.00	0.00	0.00	0.00	0.00	0.00	0.06	0.00
		5255	0.44	0.00	0.00	0.00	0.00	0.56	**0.63**	0.00	0.00	0.00
		5929	**1.00**	0.00	0.06	0.00	0.00	0.00	0.00	0.00	0.06	0.00
		6286	**1.00**	0.00	0.00	0.00	0.00	0.00	0.00	0.00	0.00	0.00
		6400	**1.00**	0.00	0.00	0.00	0.00	0.00	0.88	0.00	0.00	0.00
		6489	**1.00**	0.00	0.00	0.00	0.00	0.19	0.00	0.00	0.00	0.00
		6752	**1.00**	0.00	0.00	0.00	0.00	0.00	0.00	0.00	0.00	0.00
		9601	**1.00**	0.00	0.00	0.00	0.00	0.00	0.00	0.00	0.00	0.00
	Device	1565	**1.00**	0.00	0.06	0.00	0.00	0.00	0.19	0.00	0.19	0.38
		2733	**1.00**	0.00	0.00	0.00	0.00	0.00	0.13	0.00	0.13	0.13
		4079	**1.00**	0.00	0.00	0.00	0.00	0.00	0.00	0.00	0.00	0.00
		5255	0.31	0.00	0.00	0.00	0.00	0.63	**0.81**	0.00	0.00	0.00
		5929	**1.00**	0.00	0.00	0.00	0.00	0.00	0.00	0.00	0.06	0.00
		6286	**1.00**	0.00	0.00	0.00	0.00	0.00	0.00	0.00	0.00	0.00
		6400	**0.94**	0.00	0.00	0.00	0.00	0.00	1.00	0.00	0.00	0.00
		6489	**1.00**	0.00	0.00	0.00	0.00	0.19	0.00	0.00	0.00	0.00
		6752	**1.00**	0.00	0.00	0.00	0.00	0.00	0.00	0.00	0.00	0.00
		9601	**1.00**	0.00	0.00	0.00	0.00	0.00	0.00	0.00	0.00	0.00
1	Algorithm	0191	0.00	**1.00**	0.06	0.63	0.00	0.00	0.00	0.00	0.06	0.00
		1038	0.00	**1.00**	0.00	0.13	0.00	0.13	0.00	0.00	0.00	0.00
		3454	0.00	**1.00**	0.00	0.00	0.00	0.00	0.00	0.00	0.00	0.00
		4179	0.00	**1.00**	0.00	0.00	0.00	0.00	0.00	0.00	0.00	0.00
		4674	0.00	**1.00**	0.06	0.00	0.00	0.00	0.00	0.00	0.00	0.00
		8491	0.00	**1.00**	0.00	0.00	0.00	0.00	0.00	0.00	0.00	0.00
		8526	0.00	**1.00**	0.00	0.00	0.00	0.00	0.00	0.00	0.00	0.00
		9795	0.00	**1.00**	0.00	0.00	0.00	0.00	0.00	0.00	0.31	0.00
		9926	0.00	**1.00**	0.00	0.00	0.00	0.00	0.00	0.00	0.00	0.00
		9950	0.00	**1.00**	0.00	0.00	0.00	0.00	0.00	0.00	0.19	0.00
	Device	0191	0.00	**1.00**	0.00	0.81	0.00	0.00	0.00	0.00	0.06	0.00
		1038	0.00	**1.00**	0.00	0.13	0.00	0.13	0.00	0.00	0.00	0.00
		3454	0.00	**1.00**	0.00	0.00	0.00	0.00	0.00	0.00	0.00	0.00
		4179	0.00	**1.00**	0.06	0.00	0.00	0.00	0.00	0.00	0.00	0.00
		4674	0.00	**1.00**	0.13	0.00	0.00	0.00	0.00	0.00	0.00	0.00
		8491	0.00	**1.00**	0.06	0.00	0.00	0.00	0.00	0.00	0.06	0.00
		8526	0.00	**1.00**	0.00	0.00	0.00	0.00	0.00	0.00	0.00	0.00
		9795	0.00	**1.00**	0.00	0.00	0.00	0.00	0.00	0.00	0.63	0.00
		9926	0.00	**1.00**	0.00	0.00	0.00	0.00	0.00	0.00	0.00	0.00
		9950	0.00	**1.00**	0.00	0.00	0.00	0.00	0.00	0.00	0.50	0.00

Table S5: Spiking Rate of Each Neuron in Algorithmic Model and Device Implementation (Class 2 & Class 3)

Class	Type	Sample	Neuron									
			0	1	2	3	4	5	6	7	8	9
2	Algorithm	0298	0.00	0.00	**1.00**	0.00	0.00	0.00	0.00	0.00	0.25	0.00
		0816	0.00	0.00	**1.00**	0.25	0.00	0.00	0.00	0.00	0.31	0.00
		1177	0.00	0.00	**1.00**	0.00	0.00	0.00	0.00	0.00	0.00	0.00
		2940	0.00	0.00	**0.94**	0.13	0.00	0.00	0.13	0.00	0.00	0.00
		2971	0.00	0.00	**1.00**	0.00	0.00	0.00	0.00	0.00	0.00	0.00
		3128	0.00	0.00	**1.00**	0.56	0.00	0.00	0.00	0.00	0.06	0.00
		4504	0.13	0.00	0.38	0.00	0.00	0.06	0.00	**0.56**	0.00	0.00
		7637	0.00	0.00	0.06	**1.00**	0.00	0.00	0.00	0.00	0.00	0.00
		7785	0.00	0.00	**1.00**	0.00	0.00	0.00	0.00	0.00	0.00	0.00
		9942	0.00	0.00	**1.00**	0.00	0.00	0.00	0.00	0.00	0.00	0.00
	Device	0298	0.00	0.00	**1.00**	0.00	0.00	0.00	0.00	0.00	0.25	0.00
		0816	0.00	0.00	**1.00**	0.25	0.00	0.00	0.00	0.00	0.44	0.00
		1177	0.00	0.00	**1.00**	0.00	0.00	0.00	0.00	0.00	0.00	0.00
		2940	0.00	0.00	**1.00**	0.19	0.00	0.00	0.31	0.00	0.13	0.00
		2971	0.00	0.00	**1.00**	0.00	0.00	0.00	0.00	0.00	0.00	0.00
		3128	0.00	0.00	**1.00**	0.81	0.00	0.00	0.00	0.00	0.13	0.00
		4504	0.06	0.00	**0.69**	0.00	0.00	0.06	0.00	0.50	0.00	0.00
		7637	0.00	0.00	0.13	**1.00**	0.00	0.00	0.00	0.00	0.00	0.00
		7785	0.00	0.00	**1.00**	0.00	0.00	0.00	0.00	0.00	0.00	0.00
		9942	0.00	0.00	**1.00**	0.00	0.00	0.00	0.00	0.00	0.00	0.00
3	Algorithm	0349	0.00	0.00	0.56	0.19	0.00	0.00	0.00	**0.88**	0.13	0.00
		2639	0.00	0.00	0.00	**1.00**	0.00	0.13	0.00	0.00	0.00	0.00
		2952	0.00	0.00	0.06	**1.00**	0.00	0.94	0.00	0.00	0.06	0.00
		4833	0.00	0.00	**1.00**	1.00	0.00	0.00	0.00	0.00	0.00	0.00
		6107	0.00	0.00	0.00	**1.00**	0.00	0.00	0.00	0.00	0.00	0.00
		7312	0.00	0.00	0.00	**1.00**	0.00	0.00	0.00	0.00	0.06	0.00
		7968	0.00	0.00	0.00	**1.00**	0.00	0.00	0.00	0.00	0.00	0.00
		8393	0.00	0.00	0.06	**1.00**	0.00	0.00	0.00	0.00	0.13	0.00
		8811	0.00	0.00	0.00	**1.00**	0.00	0.06	0.00	0.00	0.00	0.00
		9342	0.00	0.00	**1.00**	0.50	0.00	0.00	0.00	0.00	0.00	0.00
	Device	0349	0.00	0.00	0.69	0.19	0.00	0.00	0.00	**0.88**	0.13	0.00
		2639	0.00	0.00	0.00	**1.00**	0.00	0.31	0.00	0.00	0.00	0.00
		2952	0.00	0.00	0.00	**1.00**	0.00	0.94	0.00	0.00	0.06	0.00
		4833	0.00	0.00	**1.00**	1.00	0.00	0.00	0.00	0.00	0.00	0.00
		6107	0.00	0.00	0.00	**1.00**	0.00	0.00	0.00	0.00	0.00	0.00
		7312	0.00	0.00	0.06	**1.00**	0.00	0.00	0.00	0.00	0.13	0.00
		7968	0.00	0.00	0.06	**1.00**	0.00	0.00	0.00	0.00	0.00	0.00
		8393	0.00	0.00	0.06	**1.00**	0.00	0.00	0.00	0.00	0.19	0.00
		8811	0.00	0.00	0.00	**1.00**	0.00	0.06	0.00	0.00	0.00	0.00
		9342	0.00	0.00	**1.00**	0.56	0.00	0.00	0.00	0.00	0.00	0.00

Table S6: Spiking Rate of Each Neuron in Algorithmic Model and Device Implementation (Class 4 & Class 5)

Class	Type	Sample	Neuron									
			0	1	2	3	4	5	6	7	8	9
4	Algorithm	3718	0.00	0.00	0.00	0.00	0.88	0.00	0.00	0.00	0.00	**1.00**
		3792	0.00	0.00	0.00	0.00	**1.00**	0.00	0.00	0.00	0.00	0.00
		4266	0.00	0.00	0.00	0.00	**1.00**	0.00	0.00	0.00	0.00	1.00
		5926	0.00	0.00	0.00	0.00	**0.94**	0.00	0.00	0.00	0.00	0.88
		7341	0.00	0.00	0.00	0.00	**1.00**	0.00	0.00	0.06	0.00	0.63
		7456	0.00	0.00	0.00	0.00	**1.00**	0.00	0.00	0.00	0.00	0.50
		8193	0.00	0.00	0.00	0.00	**1.00**	0.00	0.00	0.00	0.00	0.63
		8312	0.00	0.00	0.00	0.00	**1.00**	0.00	0.00	0.00	0.00	0.13
		9099	0.00	0.00	0.00	0.00	**1.00**	0.00	0.00	0.00	0.00	0.00
		9605	0.00	0.00	0.00	0.00	**1.00**	0.00	0.00	0.00	0.00	0.06
	Device	3718	0.00	0.00	0.00	0.00	0.88	0.00	0.00	0.06	0.00	**1.00**
		3792	0.00	0.00	0.00	0.00	**1.00**	0.00	0.00	0.00	0.00	0.00
		4266	0.00	0.00	0.00	0.00	**1.00**	0.00	0.00	0.00	0.00	1.00
		5926	0.00	0.00	0.00	0.00	**0.94**	0.00	0.00	0.00	0.00	0.69
		7341	0.00	0.00	0.00	0.00	**1.00**	0.00	0.00	0.06	0.00	0.38
		7456	0.00	0.00	0.00	0.00	**1.00**	0.00	0.00	0.00	0.00	0.44
		8193	0.00	0.00	0.00	0.00	**1.00**	0.00	0.00	0.00	0.00	0.69
		8312	0.00	0.00	0.00	0.00	**1.00**	0.00	0.00	0.00	0.00	0.13
		9099	0.00	0.00	0.00	0.00	**1.00**	0.00	0.00	0.00	0.00	0.00
		9605	0.00	0.00	0.00	0.00	**1.00**	0.00	0.00	0.00	0.00	0.06
5	Algorithm	0356	0.00	0.00	0.00	0.00	0.00	**1.00**	0.00	0.00	0.00	0.00
		1115	0.00	0.00	0.00	0.44	0.00	**0.88**	0.00	0.00	0.00	0.56
		1510	0.00	0.00	0.00	0.00	0.00	**1.00**	0.00	0.00	0.00	0.00
		1525	**0.44**	0.00	0.19	0.00	0.00	0.06	0.00	0.06	0.25	0.00
		2518	0.00	0.00	0.00	0.00	0.00	**1.00**	0.00	0.00	0.00	0.00
		2832	0.00	0.00	0.00	**0.94**	0.00	0.69	0.00	0.00	0.00	0.00
		4054	0.00	0.00	0.00	0.38	0.00	**1.00**	0.00	0.00	0.06	0.00
		4979	0.00	0.00	0.00	0.31	0.00	**1.00**	0.00	0.00	0.00	0.00
		5598	0.00	0.00	0.00	0.00	0.00	**1.00**	0.00	0.00	0.00	0.00
		7630	0.00	0.00	0.00	0.13	0.00	**1.00**	0.00	0.00	0.00	0.00
	Device	0356	0.00	0.00	0.00	0.00	0.00	**1.00**	0.00	0.00	0.00	0.00
		1115	0.00	0.00	0.00	0.56	0.00	**0.88**	0.00	0.00	0.00	0.50
		1510	0.00	0.00	0.00	0.06	0.00	**1.00**	0.00	0.00	0.00	0.00
		1525	**0.44**	0.00	0.13	0.06	0.00	0.13	0.00	0.00	0.31	0.00
		2518	0.00	0.00	0.00	0.00	0.00	**1.00**	0.00	0.00	0.00	0.00
		2832	0.00	0.00	0.00	**0.94**	0.00	0.63	0.00	0.00	0.06	0.00
		4054	0.00	0.00	0.00	0.38	0.00	**1.00**	0.00	0.00	0.06	0.00
		4979	0.00	0.00	0.00	0.50	0.00	**1.00**	0.00	0.00	0.00	0.00
		5598	0.00	0.00	0.00	0.00	0.00	**1.00**	0.00	0.00	0.00	0.00
		7630	0.00	0.00	0.00	0.31	0.00	**1.00**	0.00	0.00	0.00	0.00

Table S7: Spiking Rate of Each Neuron in Algorithmic Model and Device Implementation (Class 6 & Class 7)

Class	Type	Sample	Neuron									
			0	1	2	3	4	5	6	7	8	9
6	Algorithm	3331	0.00	0.00	0.00	0.00	0.00	0.00	**1.00**	0.00	0.00	0.00
		4239	0.00	0.00	0.00	0.00	0.00	0.44	**0.88**	0.00	0.13	0.00
		4622	0.00	0.00	0.00	0.00	0.00	0.00	**1.00**	0.00	0.13	0.00
		4814	**0.94**	0.00	0.00	0.00	0.00	0.00	0.00	0.00	0.00	0.00
		5599	0.00	0.00	0.06	0.00	0.00	0.00	**1.00**	0.00	0.00	0.00
		6258	0.00	0.00	0.00	0.00	0.00	0.00	**1.00**	0.00	0.00	0.00
		6842	0.00	0.00	0.13	0.00	0.00	0.00	**1.00**	0.00	0.00	0.00
		8423	0.00	0.00	0.13	0.00	0.00	0.00	**1.00**	0.00	0.00	0.00
		8990	0.00	0.00	0.00	0.00	0.00	0.00	**1.00**	0.00	0.00	0.00
		9149	0.00	0.00	0.00	0.00	0.00	0.00	**1.00**	0.00	0.00	0.00
	Device	3331	0.00	0.00	0.00	0.00	0.00	0.00	**1.00**	0.00	0.00	0.00
		4239	0.06	0.00	0.00	0.00	0.00	0.44	**0.94**	0.00	0.13	0.00
		4622	0.00	0.00	0.00	0.00	0.00	0.00	**1.00**	0.00	0.13	0.00
		4814	**0.94**	0.00	0.00	0.00	0.00	0.00	0.00	0.00	0.00	0.00
		5599	0.00	0.00	0.06	0.00	0.00	0.00	**1.00**	0.00	0.00	0.00
		6258	0.00	0.00	0.00	0.00	0.00	0.00	**1.00**	0.00	0.00	0.00
		6842	0.00	0.00	0.13	0.00	0.00	0.00	**1.00**	0.00	0.00	0.00
		8423	0.00	0.00	0.13	0.00	0.00	0.00	**1.00**	0.00	0.00	0.06
		8990	0.00	0.00	0.00	0.00	0.00	0.00	**1.00**	0.00	0.00	0.00
		9149	0.00	0.00	0.00	0.00	0.00	0.00	**1.00**	0.00	0.00	0.00
7	Algorithm	0617	0.00	0.00	0.63	0.00	0.00	0.00	0.00	**1.00**	0.00	0.00
		2091	0.00	0.00	0.00	0.00	0.00	0.00	0.00	**1.00**	0.00	0.00
		3225	0.00	0.56	0.19	0.44	0.00	0.06	0.00	**0.63**	0.13	0.00
		4214	0.00	0.00	0.00	0.00	0.13	0.00	0.00	**1.00**	0.00	0.13
		4693	0.00	0.00	0.00	0.00	0.00	0.00	0.00	**1.00**	0.00	0.44
		5600	0.00	0.00	0.00	0.00	0.06	0.00	0.00	0.75	0.19	**1.00**
		6589	0.00	0.00	0.31	0.00	0.00	0.00	0.00	**1.00**	0.00	0.00
		6762	0.00	0.00	0.88	0.00	0.00	0.00	0.00	**1.00**	0.00	0.00
		7069	0.00	0.00	0.00	0.00	0.00	0.00	0.00	**1.00**	0.00	0.00
		8248	0.00	0.00	0.00	0.00	0.00	0.00	0.00	**1.00**	0.00	0.00
	Device	0617	0.00	0.00	0.63	0.00	0.00	0.00	0.00	**1.00**	0.00	0.00
		2091	0.00	0.00	0.00	0.00	0.00	0.00	0.00	**1.00**	0.00	0.00
		3225	0.00	0.50	0.25	0.50	0.00	0.00	0.00	**0.63**	0.13	0.06
		4214	0.00	0.00	0.00	0.00	0.06	0.00	0.00	**1.00**	0.00	0.13
		4693	0.00	0.00	0.00	0.00	0.00	0.00	0.00	**1.00**	0.00	0.56
		5600	0.00	0.00	0.00	0.00	0.00	0.00	0.00	0.75	0.31	**1.00**
		6589	0.00	0.00	0.38	0.00	0.00	0.00	0.00	**1.00**	0.00	0.00
		6762	0.00	0.00	0.81	0.00	0.00	0.00	0.00	**1.00**	0.00	0.00
		7069	0.00	0.00	0.00	0.00	0.00	0.00	0.00	**1.00**	0.00	0.00
		8248	0.00	0.00	0.00	0.00	0.00	0.00	0.00	**1.00**	0.00	0.00

Table S8: Spiking Rate of Each Neuron in Algorithmic Model and Device Implementation (Class 8 & Class 9)

Class	Type	Sample	Neuron									
			0	1	2	3	4	5	6	7	8	9
8	Algorithm	0714	0.06	0.00	0.00	0.13	0.00	0.25	0.00	0.00	**1.00**	0.00
		1371	0.00	0.00	0.00	0.31	0.00	0.00	0.00	0.00	**1.00**	0.00
		2859	0.00	0.06	0.19	0.00	0.00	0.00	0.19	0.00	**1.00**	0.00
		3064	0.00	0.00	0.00	0.31	0.00	0.00	0.00	0.00	**1.00**	0.00
		5049	0.00	0.00	0.00	0.00	0.00	0.00	0.13	0.00	**0.94**	0.00
		6001	0.00	0.00	0.00	0.06	0.00	0.00	0.00	0.00	**1.00**	0.00
		6617	0.00	0.00	0.06	0.06	0.00	0.00	0.00	0.00	**1.00**	0.25
		6654	0.00	0.00	0.00	0.00	0.00	0.00	0.00	0.00	**1.00**	0.00
		8699	0.00	0.00	0.00	0.00	0.00	0.00	0.00	0.00	**1.00**	0.00
		8934	0.00	0.00	0.00	0.00	0.00	0.00	0.00	0.00	**1.00**	0.00
	Device	0714	0.06	0.00	0.00	0.13	0.00	0.25	0.00	0.00	**1.00**	0.00
		1371	0.00	0.00	0.00	0.44	0.00	0.00	0.00	0.00	**1.00**	0.00
		2859	0.00	0.00	0.19	0.00	0.00	0.00	0.31	0.00	**1.00**	0.00
		3064	0.00	0.00	0.00	0.31	0.00	0.00	0.00	0.00	**1.00**	0.00
		5049	0.00	0.00	0.00	0.00	0.00	0.00	0.19	0.00	**1.00**	0.00
		6001	0.00	0.00	0.00	0.06	0.00	0.00	0.00	0.00	**1.00**	0.00
		6617	0.00	0.00	0.13	0.06	0.00	0.00	0.00	0.00	**1.00**	0.25
		6654	0.00	0.00	0.00	0.00	0.00	0.00	0.00	0.00	**1.00**	0.00
		8699	0.00	0.00	0.00	0.00	0.00	0.00	0.00	0.00	**1.00**	0.00
		8934	0.00	0.00	0.00	0.00	0.00	0.00	0.00	0.00	**1.00**	0.00
9	Algorithm	0193	0.00	0.00	0.00	**0.38**	0.25	0.13	0.00	0.13	0.00	0.13
		0639	0.00	0.00	0.00	0.00	0.06	0.00	0.00	0.00	0.00	**1.00**
		0962	0.00	0.00	0.00	0.00	0.31	0.00	0.00	0.94	0.31	**1.00**
		1597	0.00	0.00	0.00	0.00	0.38	0.00	0.00	0.19	0.06	**1.00**
		3041	0.00	0.00	0.00	0.00	0.25	0.00	0.00	0.13	0.06	**1.00**
		3723	0.00	0.00	0.13	0.00	0.06	0.00	0.00	0.00	0.06	**1.00**
		4237	0.00	0.00	0.00	0.00	0.06	0.00	0.00	0.00	0.00	**1.00**
		4761	0.00	0.00	0.06	0.19	0.13	0.19	0.00	0.00	**0.50**	0.06
		8002	0.00	0.00	0.00	0.00	0.00	0.00	0.00	0.56	0.00	**1.00**
		8998	0.00	0.00	0.00	0.00	0.00	0.00	0.00	0.44	0.00	**1.00**
	Device	0193	0.00	0.00	0.06	**0.50**	0.31	0.13	0.00	0.13	0.06	0.13
		0639	0.00	0.00	0.00	0.00	0.06	0.00	0.00	0.00	0.00	**1.00**
		0962	0.00	0.00	0.00	0.00	0.25	0.00	0.00	0.88	0.44	**1.00**
		1597	0.00	0.00	0.00	0.00	0.44	0.00	0.00	0.25	0.06	**1.00**
		3041	0.00	0.00	0.00	0.00	0.19	0.00	0.00	0.25	0.06	**1.00**
		3723	0.00	0.00	0.19	0.00	0.00	0.00	0.00	0.00	0.06	**1.00**
		4237	0.00	0.00	0.00	0.00	0.06	0.00	0.00	0.00	0.00	**1.00**
		4761	0.00	0.00	0.06	0.38	0.19	0.13	0.00	0.00	**0.75**	0.06
		8002	0.00	0.00	0.00	0.00	0.00	0.00	0.00	0.56	0.00	**1.00**
		8998	0.00	0.00	0.00	0.00	0.06	0.00	0.00	0.44	0.00	**1.00**

Reference

[1]. H. S. Bindra, C. E. Lokin, A. -J. Annema and B. Nauta, "A 30fJ/comparison dynamic bias comparator," ESSCIRC 2017 - 43rd IEEE European Solid State Circuits Conference, Leuven, Belgium, 2017, pp. 71-74, doi: 10.1109/ESSCIRC.2017.8094528.
